# Organophotocatalyst Enabled Deoxycyclopropanation of Alcohols

**DOI:** 10.1002/advs.202411788

**Published:** 2024-10-29

**Authors:** Yongsheng Zhang, Jincheng Wang, Xiaoyan He, Shilin Peng, Lei Yuan, Gang Huang, Yongjin Guo, Xiuhong Lu

**Affiliations:** ^1^ Shanghai Key Laboratory of Molecular Imaging Jiading District Central Hospital Affiliated Shanghai University of Medicine and Health Sciences Shanghai 201318 P. R. China; ^2^ School of Pharmacy Shanghai University of Medicine and Health Sciences Shanghai 201318 P. R. China; ^3^ Key Laboratory of Structure‐Based Drug Design & Discovery of Ministry of Education Shenyang Pharmaceutical University Shenyang 110016 P. R. China

**Keywords:** 4CzTPN, cyclopropane, deoxycyclopropanation, NHC alcohol adducts, photocatalysis

## Abstract

Cyclopropane fragments, which widely exist in marketed drugs and natural products, can confer special pharmacological properties to small‐molecule drugs. Therefore, developing methods to construct cyclopropanes is of great significance. Nevertheless, the introduction of cyclopropane primarily relies on already‐formed cyclopropyl groups, which significantly restricts the diversity of cyclopropane skeletons. Late‐stage direct cyclopropanation is still a challenging task. Herein, a photo‐induced intermolecular deoxycyclopropanation reaction that employs alcohols as substrates, and 1 mol.% of 2,3,5,6‐tetrakis(carbazol‐9‐yl)‐1,4‐dicyanobenzene (4CzTPN) as organophotocatalyst is reported. This method proceeds with high transformation efficiency (up to 98% yield) and exhibits broad functional group tolerance, such as primary, secondary, and tertiary alcohols as well as various activated β‐halogenated alkenes. This process is mild, easy to operate, and has low equipment requirements. The power of this technology is demonstrated by the late‐stage functionalization of five marketed drugs and five natural products.

## Graphical abstract

A novel example of alcohol deoxycyclopropanation reaction for cyclopropane introduction with organophotocatalyst is described. This method demonstrates a broad substrate scope and high transformation efficiency. The mechanism involves the activation of alcohols in situ along with a photoredox‐catalyzed deoxygenative radical addition–polar cyclization. The direct introduction of cyclopropane to marketed drugs and natural products is also explored.

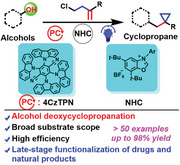

## 1 Introduction

**Figure 1:**
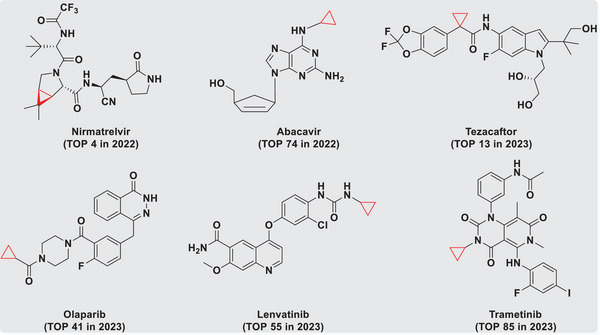
Top‐selling drugs containing cyclopropane fragments in 2022 and 2023.

Cyclopropane, as the smallest cyclic molecule, has unique structural characteristics as well as physical and chemical properties, often appearing in drugs and natural products.^[^ ^1^ ^]^ Evidence suggests that the introduction of cyclopropane can improve the activity, stability, selectivity, and bioavailability of a target molecule.^[^ ^2^ ^]^ Currently, over 60 drug molecules containing cyclopropane have been approved by the US Food and Drug Administration.^[^ ^3^ ^]^ In 2022 and 2023, six of the top‐100 selling drugs contained the cyclopropane fragment, including Nirmatrelvir (trade name Paxlovid) for Corona Virus Disease 2019, Abacavir (trade name Triumeq) for human immunodeficiency virus, Tezacaftor (trade name Trikafta) for cystic fibrosis, Olaparib (trade name Lynparza) for Ovarian Carcinoma, Lenvatinib (trade name Lenvatinib) for thyroid carcinoma, and Trametinib (trade name Mekinist) for melanoma (**Figure** **1**).^[^ ^4^ ^]^ Consequently, cyclopropane, which is ubiquitous in drugs and natural products, has gained increasing attention in chemical and medicinal fields.

Although cyclopropane was discovered in 1882,^[^ ^5^ ^]^ traditional methods for intermolecular cyclopropanation are limited and often exhibit certain disadvantages. The Simmons–Smith reaction, a classic intermolecular cyclopropanation method,^[^ ^6^ ^]^ often requires highly active organometallic reagents and unstable dihalogenated hydrocarbons to generate metal carbene intermediates for the construction of cyclopropane.^[^ ^7^ ^]^ The Johnson–Corey–Chaykovsky and Kulinkovich reactions often employ harsh conditions (e.g., strong bases such as lithium diisopropylamide) and air/moisture‐sensitive alkyl organometallics, which is inconvenient for operation.^[^ ^8^ ^]^ Furthermore, cyclopropanation generated by metal‐catalyzed decomposition of diazoesters will yield highly energetic diazoalkanes, which are dangerous to handle.^[^ ^9^ ^]^ Therefore, methodologies developed to introduce cyclopropane fragments into target molecules remain highly valued but with challenges in terms of substrate scope and green chemistry.

**Scheme 1:**
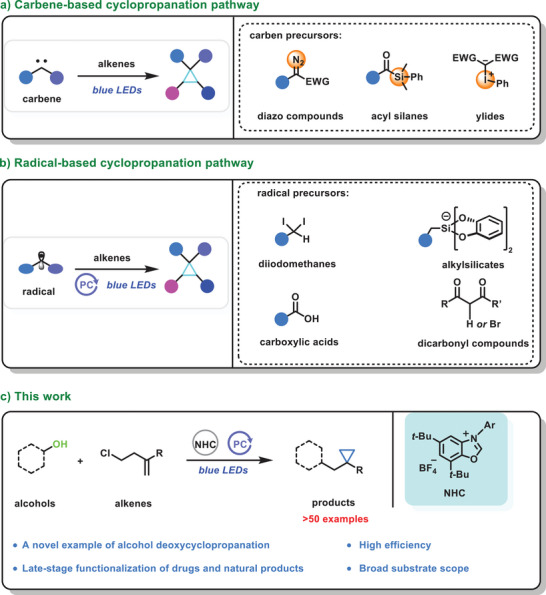
Photo‐induced strategies for the construction of cyclopropane.

In recent years, photocatalysis, with green and mild reaction conditions, high functional‐group tolerance, and diverse molecular conversions, has brought organic synthesis into another period of renaissance.^[^ ^10^ ^]^ Photo‐induced cyclopropanation mainly includes carbene‐based and radical‐involved pathways (**Scheme** **1**).^[^ ^11^ ^]^ The carbene‐based strategy requires the generation of free carbene from carbene precursors,^[^ ^12^ ^]^ whereas the radical‐involved pathway relies on the utilization of photocatalysts to induce radical precursors to produce radicals.^[^ ^13^ ^]^ These two active species can further react with alkenes to form cyclopropane fragments (Scheme 1a,b). Despite all of these advances, precursors generated from these methods for the cyclopropanation of alkenes still encounter the following problems: i) limited commercial availability,^[^ ^13e^ ^]^ ii) low conversion efficiency,^[^ ^13h^ ^]^ and iii) limited variety of active precursor,^[^ ^13a,b,i^ ^]^ which will narrow the substrate scope. Alcohols are a class of compounds widely distributed in nature and commercially abundant compared with carboxylic acids and halohydrocarbon,^[^ ^14^ ^]^ which makes them ideal radical precursors in organic synthesis.^[^ ^15^ ^]^ In 2021, MacMillan's group designed and synthesized *N*‐heterocyclic carbene (NHC)‐derived reagents that can activate alcohols in situ to generate alkyl radicals under photocatalysis conditions, then undergo a Ni‐catalyzed coupling reaction with aryl bromides.^[^ ^16^ ^]^ With this pioneer finding, other unprecedented deoxy‐functionalization has been achieved, with practical application in drug synthesis and modification.^[^ ^17^ ^]^

Based on the above‐mentioned discussion, we predicted that NHC‐alcohol adducts have potential usage in the synthesis of cyclopropane. First, widely distributed and commercially available alcohols can be used as the backbone for constructing cyclopropane, which can further expand the substrate range. Second, to the best of our knowledge, there are no reports on the construction of cyclopropane using alcohols as precursor. As alcohol hydroxyl groups are commonly present in marketed drugs and natural products, methods that are able to introduce cyclopropane groups into such products would be highly valuable. Herein, we report a photo‐enabled alcohol deoxycyclopropanation reaction with high efficiency and broad substrate scope (Scheme 1c).

## 2 Results and Discussion

### 2.1 Reaction Optimization

We initiated our study using benzyl‐4‐hydroxypiperidine‐1‐carboxylate (**1a**, **Table** **1**) and 4‐chloro‐2‐methylene‐N‐phenylbutanamide (**2a**) as model substrates. After a systematic exploration, including optimization of photocatalysts, bases, alcohol and NHC stoichiometry, and solvents (see Tables S1–S6, Supporting Information), we found that product **3a** could be obtained in 93% yield by mixing and stirring 1.3 equiv. of **1a** and NHC‐1, along with 1.6 equiv. of pyridine in tert‐butyl methyl ether (MTBE) for 15 min. The mixture was then filtered and subjected to irradiation with the addition of 1.0 equiv. of **2a**, 1 mol.% of 4CzTPN, and 2.0 equiv. of sodium acetate in MTBE/N,N‐Dimethylacetamide (DMA) (3:5, 0.0313 m) under blue light emitting diodes (LEDs). Screening of different types of photocatalysts suggested that the inexpensive, non‐metallic photocatalyst 4CzTPN was most efficient (Table 1, entries 2 and 3; also see Table S1, Supporting Information). The effect of using different bases was also evaluated, which showed that NaOAc was better able to facilitate the generation of product compared with other bases such as Quinuclidine, 1,4‐diazabicyclo(2,2,2)octane (DABCO), or Tetramethylguanidine (TMG). (Table 1 **, entries 4–6**; also see Table S2, Supporting Information). For the purpose of activating secondary alcohols, NHC‐1 showed greater advantages than NHC‐2 and NHC‐3 (Table 1 **, entries 7 and 8**). However, NHC‐2 and NHC‐3 were more suitable for the activation of primary and tertiary alcohols, respectively, in this deoxycyclopropanation reaction (see Table S6, Supporting Information). Furthermore, the MTBE/DMA solvent combination provided the highest yield of **3a** after screening different solvent systems, which was consistent with previous research (see Table S3, Supporting Information).^[^ ^16^, ^17^ ^]^ Finally, control experiments revealed that photocatalyst, base, N_2_ atmosphere, and light were all essential to maintaining the high yield of this new deoxycyclopropanation protocol (Table 1 **, entries 9−12**).

**Table 1:** Reaction optimization and control experiment.

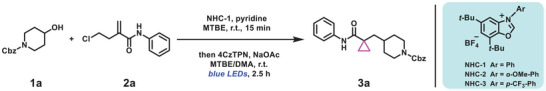		
Entry^a)^	Deviation	Yield^b)^
1	none	93%
2	4CzIPN instead of 4CzTPN	85%
3	Ir(ppy)_2_(dtbbpy)PF_6_ instead of 4CzTPN	80%
4	Quinuclidine instead of NaOAc	56%
5	DABCO instead of NaOAc	45%
6	TMG instead of NaOAc	41%
7	NHC‐2 instead of NHC‐1	41%
8	NHC‐3 instead of NHC‐1	51%
9	No photocatalyst	0%
10	No NaOAc	8.5%
11	No N_2_ sparge	32%
12	No light	0%

^a)^ Conditions: **1a** (1.3 equiv.), **2a** (0.25 mmol, 1 equiv.), NHC‐1 (1.3 equiv.), pyridine (1.6 equiv.), 1 mol.% of 4CzTPN, and sodium acetate (2.0 equiv.) in MTBE/DMA (3:5, 0.0313 m);

^b)^ Isolated yield. See the supporting information for full experimental details.

### 2.2 Substrate Scope

After determining the optimal catalysis conditions, we turned our attention to examining the scope of homoallyl chlorides in the new transformation protocol. As shown in **Table** **2**, the protocol was successful within a range of homoallyl chlorides containing electron‐withdrawing groups, such as amides (**3a‐d**, 70%–95% yield), carboxylate esters (**3e**, 64% yield), benzyl ester (**3f**, 88% yield), borate ester (**3g**, 50% yield), phosphonate esters (**3h**, 67% yield), and carboxylic acid (**3i**, 67% yield), leading to cyclopropanated products in moderate to excellent yields. For example, allyl chlorides with a diverse range of amides (**3a‐d**) were generally competent, and a gram‐scale reaction was also performed to give the target product (**3a**) in 85% yield. Secondary amides (**3a**, **3c‐d**) afforded higher yields compared with primary amides (**3b**), presumably due to the uncovered amine groups being harmful to the reaction. The reaction was not limited to electron‐withdrawing groups bearing homoallyl chlorides; styrene derivatives were also applicable substrates (**3j**, 77% yield). To our surprise, styrene derivatives with 3‐amino groups and electron‐rich heterocycles such as benzofuran were also suitable substrates for this reaction, which gave the products **3k** and **3l** in 60% and 70% yields, respectively. Moreover, the standard conditions could also accommodate disubstituted allyl chloride substrates **4**, including methyl ester and thioester substrates, which afforded the corresponding products **5a** and **5b** in good yields (73% and 78%, respectively). Finally, we further explored the reaction between primary or tertiary alcohols and 1,2‐disubstituted alkenes, through which products **5c** and **5d** were obtained in good yields (78% and 67%, respectively).

**Table 2:** Scope of the chloroalkyl alkenes.(see table footnotes).

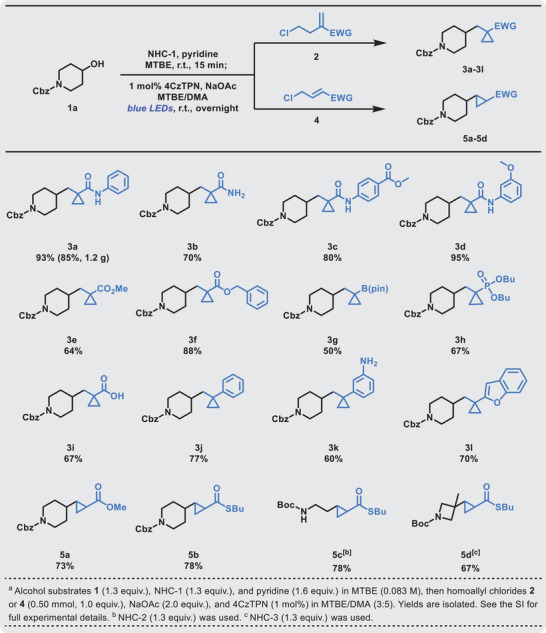

Subsequently, our focus shifted to assessing the generality of this reaction concerning alcohol substrates (refer to **Table** **3**). Initially, we investigated the capability of primary alcohols for cyclopropanation within our system. Primary alcohols bearing electron‐donating groups or electron‐withdrawing groups were both viable substrates (**6a‐f**, 50%−85% yield), and *L*‐serine was obtained in moderate yields (**6c**, 64%). Subsequently, we were delighted to find that a broad range of six‐membered ring carrying secondary alcohols, which are often present in pharmaceutical agents,^[^ ^18^ ^]^ were competent substrates in our protocol. The substrates containing cyclohexane (**6g**, 94% yield), piperidine (**6h**, 85% yield), cyclohexanone (**6i**, 90% yield), cyclohexanecarboxylicacid (**6j**, 87% yield), tetrahydropyran (**6k**, 87% yield), and 1,3‐dioxane (**6l**, 70% yield) motifs all afforded cyclopropanated products with good to excellent yields. Four‐ and five‐membered‐ring alcohols (**6m‐p**, 60%–68% yield) also performed well in this transformation. The scope of the optimized reaction conditions was further extended to secondary alcohols bearing rotationally unconstrained alkyl chains (**6q‐s**) with relatively good to excellent yields (75%–95%). Sterically encumbered polycyclic alcohols, including 2‐adamantanol and exo‐norborneol, were employed to yield cyclopropanated products without an appreciable decrease in reaction performance (**6t** and **6u**, 83% and 63% yields, respectively). Significantly, tertiary alcohols bearing quaternary carbon centers, a longstanding challenge in organic synthesis, were transformed into the corresponding cyclopropanated products in good to excellent yields using our strategy with NHC‐3 activated tertiary alcohols (**6v‐6ac**, 75%–98%).

**Table 3:** Scope of the alcohols.(see table footnotes).

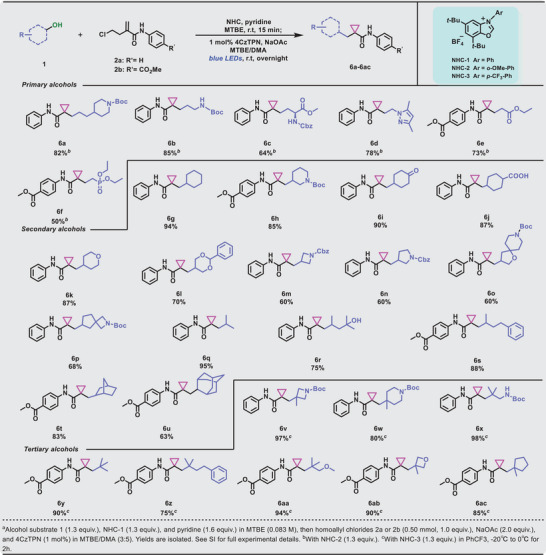

To further demonstrate the utility of this new deoxycyclopropanation strategy, we sought to derivatize a wide variety of structurally complex architectures from pharmaceutical agents and natural products (**Table** **4**). Some commercial drug molecules underwent efficient reactions with **2a** to give the corresponding products in modest to high yields (**7** and **10**, 85% and 40%, respectively). Similarly, we found that manno‐furanose and glucopyranose underwent deoxycyclopropanation at the anomeric position, providing the cyclopropane products **8** and **9** in moderate yields (75% and 70%, respectively). Next, we attempted the modification of nucleoside derivatives through our optimized conditions, which is a long‐standing challenge for organic synthesis. Pleasingly, direct transformation of the 3′‐hydroxyl group in dimethoxytrityl (DMT)‐protected thymidine (**11**) and adenosine (**12**) could be achieved in 74% and 60% yields, respectively. Steroidal substrates successfully reacted with **2a** to yield the desired natural product analogs containing cyclopropane in excellent yields (**13** and **14**, 85% and 90%, respectively), indicating that our methodology could serve as a viable alternative for complex reaction situations. Finally, cyclopropane **2b** was successfully incorporated into drug molecules. We were delighted to find that drugs such as Lenalidomide and Perphenazine could yield cyclopropane derivatives **15** and **16** in acceptable yields (28% and 60% in two steps, respectively).

**Table 4:** General scope with complex molecules.(see table footnotes).

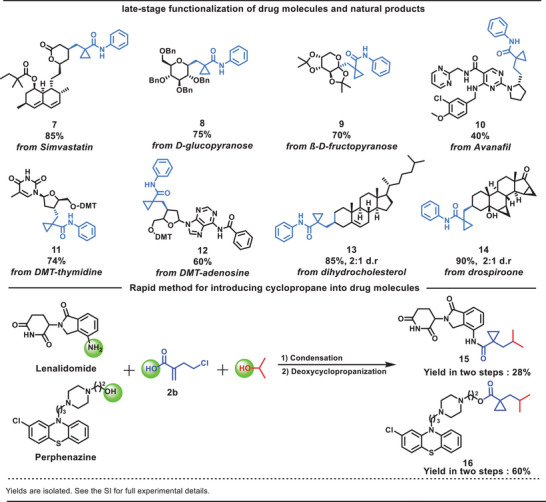

**Figure 2:**
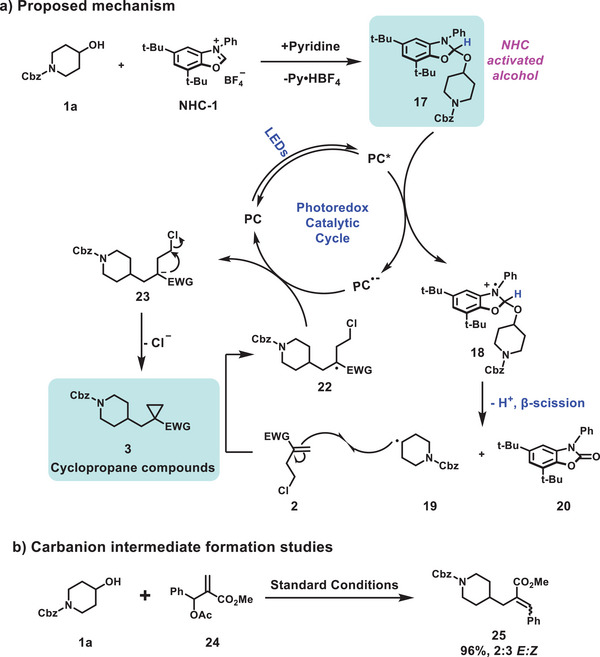
Proposed mechanism and carbanion intermediate formation studies.

The proposed mechanism for the deoxycyclopropanation reaction is outlined in **Figure** **2**a. First, aliphatic alcohol **1a** condenses with **NHC‐1** to form the photo‐active NHC‐alcohol adduct **17**.^[^ ^16^ ^]^ It is known that visible‐light irradiation of photocatalyst 4CzTPN can produce an oxidized excited state denoted as PC*. The NHC‐alcohol adduct **17** can be readily quenched by PC* to form a radical cation **18**, which will dissociate to form alkyl radical **19** and inert byproduct **20** via deprotonation and β‐scission of the alcohol C─O bond; the photocatalyst itself is reduced to the radical anion PC^•–^ form at the same time. Subsequently, the alkyl radical **19** can be captured by the homoallyl chloride **2** to form the stabilized alkyl radical **22**. Reduction of **22** by PC^•–^ will afford a carbanion intermediate **23**, as supported by the carbanion formation studies (Figure 2b). The formation of the carbanion is essential for the elimination of the acetate group in **24** to form an alkene **25**.^[^ ^19^ ^]^ Finally, the intermediate **23** will undergo a polar 3‐exo‐tet cyclization to afford the cyclopropane product **3** and the ground state photocatalyst in order to complete the photoredox catalytic cycle.

## 3 Conclusion

A new method for the synthesis of cyclopropane by photocatalysis employing alcohol substrates and homoallyl chlorides as starting fragments has been successfully developed. The protocol tolerates a broad range of functional groups in both the alcohol and the chloroalkyl alkenes substrates, highlighting the mild nature of this approach. Furthermore, the late‐stage cyclopropanation of complex natural products and pharmaceuticals further demonstrates the applicability of deoxygenative radical addition–polar cyclization cascade in complex functionalized molecular contexts. All of these characteristics suggest that this method could be a useful complementary method to photocatalyzed cyclopropanation.

## Conflict of Interest

The authors declare no conflict of interest.

## Author Contributions

Y.Z. and J.W. contributed equally to this work. G.H., Y.G., and X.L. conceived and supervised the project. Y.Z. and J.W. designed and performed the research. X.H., S.P., and L.Y. performed data analysis. Y.Z., J.W., G.H., Y.G., and X.L. wrote and formatted the manuscript.

## Supporting information



Supporting Information

## Data Availability

The data that support the findings of this study are available in the supplementary material of this article.
