# Supplementary material for: Organophotocatalyst Enabled Deoxycyclopropanation of Alcohols
Source: Adv Sci (Weinh). 2024 Oct 29;12(1):2411788. doi: 10.1002/advs.202411788 (PMC11714196; doi:10.1002/advs.202411788)
Supplement: Supplementary file 1 — Supporting Information [file ADVS-12-2411788-s001.docx]

**Supporting information**

Organophotocatalyst enabled Deoxycyclopropanation of Alcohols

*Yongsheng Zhang, Jincheng Wang, Xiaoyan He, Shilin Peng, Lei Yuan, Gang Huang, * Yongjin Guo, * Xiuhong Lu**

Yongsheng Zhang and Jincheng Wang contributed equally to this work.

**Table of Contents**

[1. General Information 2](#_Toc170842092)

[1.1. Solvents, Reagents and Starting Materials 2](#_Toc170842093)

[1.2. Chromatography and Instrumentation 2](#_Toc170842094)

[1.3. Typical Procedure for Deoxycyclopropanation Reaction, Photochemical Equipment and Setup 3](#_Toc170842095)

[2．Optimisation Studies and Control Reactions 4](#_Toc170842096)

[3. General Procedures 8](#_Toc170842097)

[4. Synthesis of Starting Materials 11](#_Toc170842098)

[4.1. Synthesis of Homoallyl Chlorides 11](#_Toc170842099)

[4.2. Synthesis of Styrene Derivatives 20](#_Toc170842100)

[4.2. Synthesis of Allyl Chlorides 23](#_Toc170842101)

[5. Product Characterization 25](#_Toc170842102)

[6. Mechanistic Studies 67](#_Toc170842103)

[7. References cited 69](#_Toc170842104)

[8. Copies of NMR and HRMS Spectra 71](#_Toc170842105)

# 1. General Information

## 1.1. Solvents, Reagents and Starting Materials

All reagents were used as received unless otherwise stated. All solvents were purified according to the method of Grubbs.^1^ The water used was de-ionized, and brine refers to a saturated aqueous solution of NaCl. Alcohol activation N-heterocyclic carbene reagents NHC-1, NHC-2, and NHC-3 were prepared according to literature procedures.^2^ Organic solvents were redistilled under reduced pressure on a Büchi rotary evaporator.

## 1.2. Chromatography and Instrumentation

Column chromatography was carried out using silica gel (Qing Dao Ocean, 300-400 mesh). Analytical thin-layer chromatography was performed using glass-backed silica plates (Qing Dao Huanghai, silica gel 60 F254). Compounds were visualized under UV light or by staining with aqueous basic potassium permanganate.

^1^H-NMR spectra were recorded on a WNMR-1 400MHz spectrometer (Q. One Instruments Ltd). ^13^C-NMR spectra were recorded on a WNMR-1 101MHz spectrometer (Q. One Instruments Ltd). All NMR spectra were recorded at 25 °C unless otherwise stated. Chemical shifts (δ) are given in parts per million (ppm) and referenced to CDCl_3_ (7.26 ppm and 77.16 ppm) or DMSO-d_6_ (2.50 ppm and 39.52 ppm). Coupling constants (*J*) are given in Hertz (Hz) and refer to apparent multiplicities (e.g., s = singlet, br. s = broad singlet, d = doublet, t = triplet, q = quartet, quin = quintet, sex = sextet, h = heptet, m = multiplet, dd = doublet of doublets). The ^1^H NMR spectra are reported as follows: chemical shift (multiplicity, coupling constants, number of protons). HPLC-MS spectra were obtained on an Aglient 1260-6120 electron spray ionization-quadrupole mass spectrometer. HRMS spectra were acquired with AB SCIEX TripleTOF 4600 mass spectrometers. HPLC analyses were recorded on an Agilent 1260 HPLC (C18, mobile phase A: water; mobile phase B: acetonitrile). Optical rotation was recorded on a WZZ-2S automatic polarimeter (Shanghai INESA Physico- Optical Instrument Co., Ltd.)

## 1.3. Typical Procedure for Deoxycyclopropanation Reaction, Photochemical Equipment and Setup

To an oven-dried 20-mL vial equipped with a stir bar was added NHC-1 salt (0.325 mmol, 1.3 equiv.) and alcohol substrate (0.325 mmol, 1.3 equiv.). The vial was placed under nitrogen atmosphere, and anhydrous MTBE (2 mL) was added via syringe. A pyridine solution (31.64 mg, 0.4mmol, 1.6 equiv. in 1.0 mL methyl tert-butyl ether) was added in one portion, and the suspension was stirred at room temperature under nitrogen atmosphere. 15 minutes later, the resulting mixture was filtered by syringe filter and added into a stock solution of 4CzTPN (2.0 mg, 2.5 µmol, 0.01 equiv.), alkenyl chloride (0.25 mmol, 1.0 equiv.), and NaOAc (41.02 mg, 0.5 mmol, 2.0 equiv.) in DMA (5.0 mL) (0.0313 M final concentration in 3:5 MTBE/DMA). The resulting suspension was sparged with nitrogen for 15 minutes, then the vial was sealed. The reaction was stirred at 1500 rpm and irradiated under 450 nm LED modules with a maximum fan speed of 1500 rpm in an integrated photoreactor (apparatus shown in **Figure S1**) for 2.5 h.

After completed, the reaction mixture was diluted with water (50 mL) and EtOAc (40 mL). The aqueous layer was further extracted with EtOAc three times (30 mL × 3). The combined organic layer was washed with water and brine, and dried over anhydrous magnesium sulfate. The desiccant was filtered out, and the solution was concentrated to yield the crude mixture. The product was purified via silica gel column chromatography.

The blue LED lamps used were three 10-W LED strips (450 nm, Xuzhou Ai Jia electronic technology Co., Ltd.). The reaction vials were positioned 3–5-cm away from the three 10-W LED strips (Figure S1). During the process of the photoredox reactions, heat generated from the LED strips resulted in warming of the reaction mixtures to approximately 50 °C, so fan cooling was used to maintain a temperature of 25–30 °C.

**Reaction set-up:**


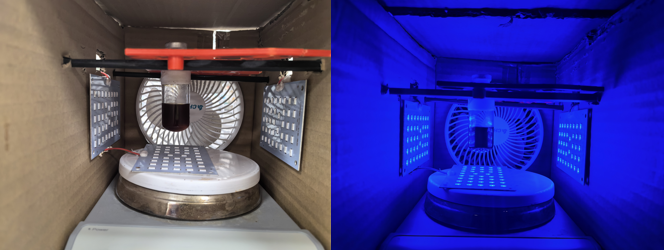


**Figure S1.** Photoredox reaction setup, this device can perform 6 reactions simultaneously.

# 2．Optimization Studies and Control Reactions

General Procedure for Reaction Optimization (0.25 mmol scale):

To an oven-dried 20 mL vial equipped with a stir bar was added **NHC-1** salt (0.325 mmol, 1.3 equiv.) and alcohol substrate **1a** (0.325 mmol, 1.3 equiv.). The vial was placed under nitrogen atmosphere, and anhydrous MTBE (2 mL) was added via syringe. A pyridine solution (31.64 mg, 0.4mmol, 1.6 equiv. in 1.0 mL methyl tert-butyl ether) was added dropwise, and the suspension was stirred at room temperature under nitrogen atmosphere. 15 minutes later, the resulting mixture was filtered by syringe filter and added into a stock solution of **4CzTPN** (2.0 mg, 2.5 µmol, 0.01 equiv.), alkenyl chloride **2a** (0.25 mmol, 1.0 equiv.), and NaOAc (41.02 mg, 0.5 mmol, 2.0 equiv.) in DMA (5.0 mL) (0.0313 M final concentration in 3:5 MTBE/DMA). The resulting suspension was sparged with nitrogen for 15 minutes, and then the vial was sealed. The reaction was stirred at 1500 rpm and irradiated under 450 nm LED strips under maximum fan speed for 2.5 h.

The following work-up procedure was adapted in general procedure for reaction optimization: the reaction mixture was diluted with water (25 mL) and EtOAc (20 mL). The aqueous layer was further extracted with EtOAc three times (15 mL × 3). The combined organic layers were washed with water and brine, and dried over anhydrous magnesium sulfate. The desiccant was filtered out, and the solution was concentrated to yield the crude mixture, which was purified via silica gel column chromatography (20% EtOAc/petroleum ether) to give the pure product **3a** as a white solid.

**Table S1.** Screen of different photocatalysts

| **entry** | **Photocatalyst** | **yield** |
| --- | --- | --- |
| 1 | 4CzTPN | 93% |
| 2 | 4CzIPN | 85% |
| 3 | 4CzPN | 75% |
| 4 | Ir(ppy)_3_ | 0% |
| 5 | Ir(ppy)_2_(dtbbpy)PF_6_ | 80% |
| 6 | Ir(dFCF_3_ppy)_2_(bpy)PF_6_ | 45% |
| 7 | Ir(dFCF_3_ppy)_2_(dtbpy)PF_6_ | 54% |
| 8 | Ru(bpy)_3_Cl_2_ | < 5% |

**Table S2.** Screen of different bases

| **Base** | **yield** | **Base** | **yield** |
| --- | --- | --- | --- |
| NaOAc | 93% | pyridine | 28% |
| Quinuclidine | 56% | NaOH | 20% |
| DABCO | 45% | Cs_2_CO_3_ | 17% |
| TMG | 41% | *t*-BuNa | <10% |
| 2,4,6-trimethylpyridine | 30% | KH_2_PO_4_ | <10% |

**Table S3.** Screen of different solvents

| **Solution A /** **Solution B** | **yield** | **Solution A /** **Solution B** | **yield** |
| --- | --- | --- | --- |
| Cyclopentyl Methyl Ether/DMA | 41% | *t*-BuOMe/DMF | 41% |
| DCM/DMA | 31% | *t*-BuOMe/DMSO | 61% |
| THF/DMA | 70% | *t*-BuOMe/MeCN | 32% |
| *t*-BuOMe/DMA | 93% | *t*-BuOMe/Acetone | 52% |
| DCE/DMA | 32% |  |  |

**Table S4.** Effect of alcohol and NHC stoichiometry

| **entry** | **equiv. of alcohol** | **equiv. of NHC** | **yield** |
| --- | --- | --- | --- |
| 1 | 1.3 | 1.3 | 93% |
| 2 | 1.3 | 1.6 | 85% |
| 3 | 1.3 | 1.4 | 81% |
| 4 | 1.3 | 1.2 | 67% |
| 5 | 1.3 | 1.0 | 45% |

**Table S5.** Control experiments

| **entry** | **deviation** | **yield** |
| --- | --- | --- |
| 1 | none | 93% |
| 2 | no N_2_ sparge | 32% |
| 3 | no light | 0% |
| 4 | no photocatalyst | 0% |
| 5 | no NaOAc | 8.5% |

**Table S6.** Evaluation of the Activation Efficiency of Different Alcohols by Different NHCs

| Alcohol | **NHC** | **yield** |
| --- | --- | --- |
|  | NHC-1 | 26% |
|  | NHC-2 | 85% |
|  | NHC-3 | 38% |
|  | NHC-1 | 93% |
|  | NHC-2 | 41% |
|  | NHC-3 | 51% |
|  | NHC-1 | 70% |
|  | NHC-2 | 50% |
|  | NHC-3 | 97% |

**NOTE:** NaOAc is well-precedented to facilitate generation of α-amino radicals via oxidation deprotonation (see McNally, A.; Prier, C. K.; MacMillan, D. W. C. Science 2011, 334, 1114-1117).

# 3. General Procedures for Deoxycyclopropanation of NHC-Alcohol Adducts

General Procedure A **(for primary and less hindered alcohol only):**

To an oven-dried 20-mL vial was added primary alcohol (0.65 mmol, 1.3 equiv.), **NHC-2** (276.42 mg, 0.65 mmol, 1.3 equiv.), and a magnetic stir bar. After the vial was vacuumed and refilled with nitrogen gas twice, methyl tert-butyl ether (5.0 mL) was added, and the reaction stirred at room temperature for 5 min. Then, a pyridine solution (63.3 mg, 0.8 mmol, 1.6 equiv. in 1.0 ml methyl tert-butyl ether) was added dropwise at room temperature over the course of 2 min. The resulting solution was stirred at room temperature for 10 min. A white solid precipitated out during this time. If the alcohol was a liquid, it was added as a methyl tert-butyl ether solution prior to the addition of the pyridine solution.

To a different oven-dried 20-mL vial was added **4CzTPN** (4.0 mg, 0.5 μmol, 0.01 equiv.), NaOAc (82.03 mg, 1.0 mmol, 2.0 equiv.), alkenyl chloride (0.5 mmol, 1.0 equiv.), and a magnetic stir bar. Dimethylacetamide (10 mL) was added to this vial under an atmosphere of nitrogen. The afore mentioned prepared methyl tert-butyl ether suspension was transferred to a 10-mL syringe under air. Then a syringe filter and new needle were installed on the syringe, before the methyl tert-butyl ether solution was injected through the syringe filter into the dimethylacetamide solution. The reaction mixture was sparged with nitrogen for 15 minutes before sealing. The vial was stirred at 1500 rpm and irradiated under 450-nm LED strips under maximum fan speed for 2.5 h.

The following work-up procedure was adapted in general procedure A: the reaction mixture was diluted with water (50 mL) and EtOAc (40 mL). The aqueous layer was further extracted with EtOAc three times (30 mL × 3). The combined organic layer was washed with water and brine, and dried over anhydrous magnesium sulfate. The desiccant was filtered out, and the solution was concentrated to yield the crude mixture. The product was purified via silica gel column chromatography.

General Procedure B: (for secondary and less hindered alcohol only).

To an oven-dried 20-mL vial was added secondary alcohol (0.65 mmol, 1.3 equiv.), **NHC-1** (256.91 mg, 0.65 mmol, 1.3 equiv.), and a magnetic stir bar. After the vial was vacuumed and refilled with nitrogen gas twice, methyl tert-butyl ether (5.0 mL) was added and the reaction stirred at room temperature for 5 min. Then, a pyridine solution (63.3 mg, 0.8 mmol, 1.6 equiv. in 1.0 ml methyl tert-butyl ether) was added dropwise at room temperature over the course of 2 min. The resulting solution was stirred at room temperature for 10 min. A white solid precipitated out during this time. If the alcohol was liquid, it was added as a methyl tert-butyl ether solution prior to the addition of the pyridine solution.

To a different oven-dried 20-mL vial was added **4CzTPN** (4.0 mg, 0.5 μmol, 0.01 equiv.), NaOAc (82.03 mg, 1.0 mmol, 2.0 equiv.), alkenyl chloride (0.5 mmol, 1.0 equiv), and a magnetic stir bar. Dimethylacetamide (10 mL) was added to this vial under an atmosphere of nitrogen. The afore mentioned prepared methyl tert-butyl ether suspension was transferred to a 10-mL syringe under air. Then a syringe filter and new needle were installed on the syringe, before the methyl tert-butyl ether solution was injected through the syringe filter into the dimethylacetamide solution. The reaction mixture was sparged with nitrogen for 15 minutes before sealing. The vial was stirred at 1500 rpm and irradiated under 450-nm LED strips under maximum fan speed for 2.5 h.

The following work-up procedure was adapted in general procedure B: the reaction mixture was diluted with water (50 mL) and EtOAc (40 mL). The aqueous layer was further extracted with EtOAc three times (30 mL × 3). The combined organic layer was washed with water and brine, and dried over anhydrous magnesium sulfate. The desiccant was filtered out, and the solution was concentrated to yield the crude mixture. The product was purified via silica gel column chromatography.

General Procedure C: (for hindered tertiary alcohols only).

To an oven-dried 20-mL vial was added tertiary alcohol (0.65 mmol, 1.3 equiv.), **NHC-3** (301.1 mg, 0.65 mmol, 1.3 equiv.), and a magnetic stir bar. After the vial was vacuumed and refilled with nitrogen gas twice, trifluorotoluene (5.0 mL) was added, and the reaction stirred at room temperature for 2 min. Then, the crude reaction mixture was cooled to –25 ℃ in an ethanol cold-bath. A pyridine solution (63.3 mg, 0.8 mmol, 1.6 equiv. in 1.0 ml trifluorotoluene) was added dropwise via a microsyringe at –25 ℃. The resulting solution was allowed to slowly warm to 0 ℃ over the course of 2 h, with the reaction sometimes turning red depending on the alcohol employed. If the alcohol was liquid, it was added as a trifluorotoluene solution prior to cooling the reaction to –25℃.

To a different oven-dried 20-mL vial was added 4CzTPN (4.0 mg, 0.5 μmol, 0.01 equiv.), NaOAc (82.03 mg, 1.0 mmol, 2.0 equiv.), alkenyl chloride (0.5 mmol, 1.0 equiv), and a magnetic stir bar. Dimethylacetamide (10 mL) was added to this vial under an atmosphere of nitrogen. The afore mentioned prepared trifluorotoluene suspension was transferred to a 10-mL syringe under air. Then a syringe filter and new needle were installed on the syringe, before the trifluorotoluene solution was injected through the syringe filter into the dimethylacetamide solution. The reaction mixture was sparged with nitrogen for 15 minutes before sealing. The vial was stirred at 1500 rpm and irradiated under 450-nm LED strips under maximum fan speed for 2.5 h.

The following work-up procedure was adapted in general procedure C: the reaction mixture was diluted with water (50 mL) and EtOAc (40 mL). The aqueous layer was further extracted with EtOAc three times (30 mL × 3). The combined organic layer was washed with water and brine, and dried over anhydrous magnesium sulfate. The desiccant was filtered out, and the solution was concentrated to yield the crude mixture. The product was purified via silica gel column chromatography.

# 4. Synthesis of Starting Materials

## 4.1. Synthesis of Homoallyl Chlorides

**4-chloro-2-methylene-*N*-phenylbutanamide** (**2a)**

4-chloro-2-methylenebutanoic acid (**2i**) was prepared following a modified literature procedure:^3^ A tube containing a magnetic stir bar was charged with Ni(acac)_2_ (128 mg, 0.25 mmol, 5.0 mol%) and 1,2-bis(diphenylphosphino)benzene (300 mg, 0.70 mmol, 7.00 mol%). The tube was evacuated and backfilled with N_2_ three times. Toluene (15 mL) was added, followed by 4-chlorobut-1-yne (**S1**) (870 mg, 10.0 mmol, 1.00 equiv.), formic acid (690 mg, 15.0 mmol, 1.50 equiv.), and pivalic anhydride (372 mg, 2.00 mmol, 20.0 mol%). The reaction mixture was heated to 100 °C for 24 h before cooling to room temperature and concentrating *in vacuo*. The residue was purified by flash column chromatography (10% EtOAc/hexane and 0.3% acetic acid) to give the 4-chloro-2-methylenebutanoic acid (**2i**) (980 mg, 7.30 mmol, 73%) as a yellow oil.

**TLC**: R_f_ = 0.20 (10% EtOAc/hexane and 0.3 % acetic acid, KMnO_4_ stain).

**^1^H NMR** (400 MHz, CDCl_3_) *δ* 11.40 (s, 1H), 6.51 (d, *J* = 1.1 Hz, 1H), 5.88 (q, *J* = 1.2 Hz, 1H), 3.73 (t, *J* = 6.8 Hz, 2H), 2.82 (td, *J* = 6.8, 1.1 Hz, 2H).

**LCMS** (ESI^+^) calcd. for C_5_H_7_ClO_2_ [M+H] ^+^ 135.0, found 135.1.

4-chloro-2-methylene-*N*-phenylbutanamide (**2a**) was prepared according to the following procedure: oxalyl chloride (370 mg, 2.9 mmol, 1.3 equiv.) was added to a solution of 4-chloro-2-methylenebutanoic acid (**2i**) (300 mg, 2.23 mmol, 1.0 equiv.) in dichloromethane (5 mL) at 0 °C. To the solution was added a few drops of DMF. The reaction was continued for 2 h at room temperature before dichloromethane was removed *in vacuo*. The prepared acyl chloride in dichloromethane (5 mL) was added dropwise to a solution of aniline (210 mg, 2.23 mmol, 1.0 equiv.) and triethylamine (340 mg, 3.35 mmol, 1.5 equiv.) at 0 °C under N_2_, then the reaction was continued for 2 h at room temperature. The organic solvent was removed *in vacuo*, and the resulting residue was purified by flash column chromatography (5% EtOAc/hexane) to give 4-chloro-2-methylene-*N*-phenylbutanamide (**2a**) (280 mg, 1.3 mmol, 44%) as a white solid, with a melting point of 43.2–44.1 ℃.

**TLC**: R_f_ = 0.50 (10% EtOAc/hexane, KMnO_4_ stain).

**^1^H NMR** (400 MHz, CDCl_3_) *δ* 7.62 (s, 1H), 7.56 – 7.52 (m, 2H), 7.35 (d, *J* = 7.8 Hz, 1H), 7.18 – 7.11 (m, 1H), 5.80 (s, 1H), 5.57 (d, *J* = 1.2 Hz, 1H), 3.73 (t, *J* = 6.5 Hz, 2H), 2.87 (td, *J* = 6.5, 1.2 Hz, 2H).

**^13^C NMR** (101 MHz, CDCl_3_) *δ* 166.15, 142.70, 137.63, 129.11, 124.69, 120.28, 120.11, 42.91, 35.92.

**HRMS** (ESI^+^) calcd for C_11_H_12_ClNO [M+H]^+^ 210.0680, found 210.0682.

**Methyl 4-(4-chloro-2-methylenebutanamido) benzoate (2b)**

Methyl 4-(4-chloro-2-methylenebutanamido) benzoate (**2b**) was obtained according to the preparation of **2a**. Methyl 4-aminobenzoate (224 mg, 1.86 mmol, 1.0 equiv.) was used and the resulting residue was purified by flash column chromatography (10% EtOAc/hexane) to give **2b** (278 mg, 1.04 mmol, 56%) as a yellow sticky oil.

**TLC**: R_f_ = 0.50 (20% EtOAc/hexane, KMnO_4_ stain).

**^1^H NMR** (400 MHz, CDCl_3_) *δ* 8.07 (s, 1H), 8.03 – 7.98 (m, 2H), 7.70 – 7.62 (m, 2H), 5.86 (s, 1H), 5.61 (d, *J* = 1.2 Hz, 1H), 3.90 (s, 3H), 3.71 (t, *J* = 6.5 Hz, 2H), 2.86 (td, *J* = 6.5, 1.1 Hz, 2H).

**^13^C NMR** (101 MHz, CDCl_3_) *δ* 166.66, 166.33, 142.19, 142.00, 130.84, 125.89, 121.19, 119.25, 52.09, 42.86, 35.76.

**HRMS** (ESI^+^) calcd for C_13_H_14_ClNO_3_ [M+H]^+^ 268.0735, found 268.0738.

**4-chloro-*N*-(3-methoxyphenyl)-2-methylenebutanamide (2c)**

4-chloro-*N*-(3-methoxyphenyl)-2-methylenebutanamide (**2c**) was obtained according to the preparation of **2a**. 3-Methoxyaniline (230 mg, 1.86 mmol, 1.0 equiv.) was used and the resulting residue was purified by flash column chromatography (5% EtOAc/hexane) to give **2c** (200 mg, 0.84 mmol, 45%) as a yellow sticky oil.

**TLC**: R_f_ = 0.30 (10% EtOAc/hexane, KMnO_4_ stain).

**^1^H NMR** (400 MHz, CDCl_3_) *δ* 7.96 (s, 1H), 7.30 (t, *J* = 2.3 Hz, 1H), 7.19 (t, *J* = 8.2 Hz, 1H), 7.07 – 6.99 (m, 1H), 6.66 (ddd, *J* = 8.3, 2.5, 0.9 Hz, 1H), 5.79 (s, 1H), 5.52 (d, *J* = 1.3 Hz, 1H), 3.76 (s, 3H), 3.68 (t, *J* = 6.6 Hz, 2H), 2.82 (td, *J* = 6.6, 1.2 Hz, 2H).

**^13^C NMR** (101 MHz, CDCl_3_) δ 166.43, 160.17, 142.31, 138.97, 129.71, 120.66, 112.48, 110.53, 106.04, 55.33, 42.95, 35.83.

**HRMS** (ESI^+^) calcd for C_12_H_14_ClNO_2_ [M+H]^+^ 240.0785, found 240.0786.

**4-Chloro-2-methylenebutanamide (2d)**^4^

4-Chloro-2-methylenebutanamide (**2d**) was prepared following a modified literature procedure:^4^ Oxalyl chloride (1.4 g, 10.97 mmol, 1.51 equiv.) was added to a solution of 4-chloro-2-methylenebutanoic acid (**2i**) (980 mg, 7.31 mmol, 1.00 equiv.) in dichloromethane (30 mL) at 0 °C. To the solution was added a few drops of DMF. The reaction was continued for 1.5 h at r.t. before cooling to 0 °C and adding 25-30% wt. ammonium hydroxide (20 mL). The organic solvent was removed *in vacuo*, and the resulting residue was purified by flash column chromatography (40% EtOAc/hexane) to give 4-chloro-2-methylenebutanamide (**2d**) (810 mg, 6.06 mmol, 82%) as a brown sticky oil.

**TLC**: R_f_ = 0.30 (50% EtOAc/hexane, KMnO_4_ stain).

**^1^H NMR** (400 MHz, CDCl_3_) δ 5.89 (d, *J* = 25.1 Hz, 1H), 5.76 (s, 1H), 5.65 (d, *J* = 47.2 Hz, 1H), 5.55 – 5.52 (m, 1H), 3.69 (t, *J* = 6.6 Hz, 2H), 2.79 (td, *J* = 6.6, 1.3 Hz, 2H).

**LCMS** (ESI^+^) calcd. for C_5_H_8_ClNO [M+H]^+^ 134.1, found 134.1.

**Methyl 4-chloro-2-methylenebutanoate (2e)**^5^

Methyl 4-chloro-2-methylenebutanoate (**2e**) was prepared following a modified literature procedure:^5^ To a flask equipped with a magnetic stir bar was added methyl 2-(bromomethyl) acrylate (**S2**) (1.0 g, 5.6 mmol, 1.00 equiv.). H_2_O (10 mL) and EtOH (10 mL) were then added with vigorous stirring, followed by formaldehyde (0.300 g, 10.1 mmol, 1.80 equiv.) and indium powder (0.71 g, 6.2 mmol, 1.1 equiv.). The reaction mixture was stirred vigorously for 20 h, then partitioned between CH_2_Cl_2_ (50 mL) and H_2_O (50 mL). The phases were separated, and the aqueous phase was extracted into CH_2_Cl_2_ (3 × 25 mL). The combined organic phases were washed with brine (50 mL), dried (MgSO_4_), filtered, and concentrated *in vacuo.* The residue was purified by flash column chromatography (10% EtOAc/hexane) to give methyl 4-hydroxy-2-methylenebutanoate (**S3**) (600 mg, 4.61 mmol, 82%) as a pale-yellow oil.

To a stirred solution of methyl 4-hydroxy-2-methylenebutanoate (**S3**) (510 mg, 3.92 mmol, 1.00 equiv.) in CH_2_Cl_2_ (20 mL) under N_2_ at 0 °C was added dry pyridine (0.4 mL) followed by dropwise addition of SOCl_2_ (0.75 mL, 10 mmol, 2.6 equiv.). The resulting solution was heated to reflux for 10 h, after which the reaction mixture was cooled to r.t. before diluting with Et_2_O (30 mL) and slowly adding H_2_O (20 mL). The phases were separated, and the aqueous phase was extracted into Et_2_O (3 × 30 mL). The organic extracts were combined, washed with H_2_O (30 mL) and brine (30 mL), then dried (MgSO_4_), filtered, and concentrated *in vacuo*. The residue was purified by flash column chromatography (20% EtOAc/hexane) to give methyl 4-chloro-2-methylenebutanoate (**2e**) (348 mg, 2.35 mmol, 60%) as a colorless oil.

**TLC**: R_f_ = 0.50 (20% EtOAc/hexane, KMnO_4_ stain).

**^1^H NMR** (500 MHz, CDCl_3_) *δ* 6.31 (d, *J* = 1.2 Hz, 1H), 5.71 (q, *J* = 1.2 Hz, 1H), 3.77 (s, 3H), 3.67 (t, *J* = 6.8 Hz, 2H), 2.77 (td, *J* = 6.8, 1.2 Hz, 2H).

**LCMS** (ESI^+^) calcd. for C_6_H_9_ClO_2_ [M+H] ^+^ 149.1, found 149.1.

**Benzyl 4-chloro-2-methylenebutanoate (2f)**^6^

4-chloro-*N*-(3-methoxyphenyl)-2-methylenebutanamide (**2f**) was obtained according to the preparation of **2a**. Phenylmethanol (200 mg, 1.85 mmol, 1.0 equiv.) was used and the resulting residue was purified by flash column chromatography (20% EtOAc/hexane) to give **2f** (220 mg, 0.97 mmol, 52%) as a yellow sticky oil.

**TLC**: R_f_ = 0.20 (20% EtOAc/hexane, KMnO_4_ stain).

**^1^H NMR** (400 MHz, CDCl_3_) *δ* 7.45 – 7.41 (m, 5H), 6.42 (d, *J* = 1.1 Hz, 1H), 5.79 (t, *J* = 1.2 Hz, 1H), 5.27 (s, 2H), 3.73 (t, *J* = 6.8 Hz, 2H), 2.85 (td, *J* = 6.8, 1.2 Hz, 2H).

**LCMS** (ESI^+^) calcd. for C_12_H_13_ClO_2_ [M+H] ^+^ 225.0, found 225.1.

**2-(4-Chlorobut-1-en-2-yl)-4,4,5,5-tetramethyl-1,3,2-dioxaborolane (2g)^6^**

2-(4-Chlorobut-1-en-2-yl)-4,4,5,5-tetramethyl-1,3,2-dioxaborolane (**2g**) was prepared following a modified literature procedure:^6^ A vial containing a magnetic stir bar was charged with Pd (PPh_3_)_4_ (0.25 g, 0.21 mmol, 3.0 mol%) and bis(pinacolato)diboron (2.18 g, 8.6 mmol, 1.20 equiv.). The vial was evacuated and backfilled with nitrogen three times. Toluene (15 mL) was added, followed by but-3-yn-1-ol (**S4**) (500 mg, 7.1 mmol, 1.00 equiv.) and acetic acid (426 mg,7.1 mmol, 1.00 equiv.). The reaction mixture was pre-stirred for 5 min before heating to 80 °C overnight. After cooling to r.t., the mixture was concentrated *in vacuo* and the residue was purified by flash column chromatography (20% EtOAc/hexane) to give the 3-(4,4,5,5-tetramethyl-1,3,2-dioxaborolan-2-yl) but-3-en-1-ol (**S5**) (1.3 g, 6.56 mmol, 92%) as a brown oil.

To an oven-dried flask was added 3-(4,4,5,5-tetramethyl-1,3,2-dioxaborolan-2-yl)but-3-en-1-ol (**S5**) (1.03 mL, 5.05 mmol, 1.00 equiv.) and pyridine (40 μL, 0.50 mmol, 0.10 equiv.) and the mixture was cooled to 0 °C. Thionyl chloride (0.370 mL, 5.05 mmol, 1.00 equiv.) was added dropwise before the reaction mixture was heated to 70 °C for 16 h. After cooling to r.t., the reaction mixture was concentrated *in vacuo* and purified by flash column chromatography (2% EtOAc/pentane) to give **2g** (877 mg, 4.05 mmol, 80%) as a pale-yellow liquid.

**TLC**: R_f_ = 0.45 (5% Et_2_O/pentane, KMnO_4_ stain).

**^1^H NMR** (500 MHz, CDCl_3_) *δ* 5.92 (d, *J* = 3.1 Hz, 1H), 5.78 – 5.68 (m, 1H), 3.62 (t, *J* = 7.3 Hz, 2H), 2.61 (t, *J* = 7.3 Hz, 2H), 1.27 (s, 12H).

**LCMS** (ESI^+^) calcd. for C_10_H_18_BClO_2_ [M+H] ^+^ 217.1, found 217.1.

**Dibutyl (4-chlorobut-1-en-2-yl) phosphonate (2h)^7^**

Dibutyl (4-hydroxybut-1-en-2-yl) phosphonate (**S5**) was prepared following a modified literature procedure:^7^ Under argon, Pd_2_(dba)_3_ (200 mg, 0.215 mmol, 3.00 mol%) and Ph_3_P (225 mg, 0.855 mmol, 12.0 mol%) were placed in a septum-sealed tube equipped with a magnetic stir bar. THF (10 mL) was added, and the mixture was stirred for 3 min before dibutyl phosphonate (1.39 g, 7.135 mmol, 1.00 equiv.) and alkyne **S4** (0. 5 g, 7.135 mmol, 1.0 equiv.) were added. TFA (85 mg, 0.7135 mmol, 10 mol%) was added and the tube was capped with a PTFE-sealed screw cap. The mixture was stirred at 50 °C for 8 h, then H_2_O (20 mL) was added carefully, and the mixture was extracted with EtOAc (3 × 20 mL). The combined organic phases were dried (MgSO_4_), filtered, and concentrated *in vacuo*. The residue was purified by flash column chromatography (80% EtOAc/hexane) to afford dibutyl (4-hydroxybut-1-en-2-yl) phosphonate (**S6**) (700 mg, 2.65 mmol, 40%) as a yellow oil.

Homoallyl chloride **2h** was then prepared following the same chlorination procedure as described in the synthesis of **2e**, with alcohol **S6** (700 mg, 2.65 mmol, 1.00 equiv.). The crude product was purified by flash column chromatography (10% EtOAc/hexane) to afford **2h** (600 mg, 2.12 mmol, 80%) as a yellow oil.

**TLC**: R_f_ = 0.50 (50% EtOAc/hexane, KMnO_4_ stain).

**^1^H NMR** (400 MHz, CDCl_3_) *δ* 6.15 (d, *J* = 20.9 Hz, 1H), 5.89 (d, *J* = 47.4 Hz, 1H), 4.10 – 3.95 (m, 4H), 3.69 (t, *J* = 7.0 Hz, 2H), 2.72 (ddd, *J* = 14.1, 7.6, 6.4 Hz, 2H), 1.74 – 1.59 (m, 4H), 1.42 (dt, *J* = 15.0, 7.4 Hz, 4H), 0.94 (t, *J* = 7.4 Hz, 6H).

**LCMS** (ESI^+^) calcd. for C_12_H_24_ClO_3_P [M+H]^+^ 283.1, found 283.1.

**4-Chloro-*N*-(2-(2,6-dioxopiperidin-3-yl)-1-oxoisoindolin-4-yl)-2-methylenebutana**

**-mide** (**2j)**

4-chloro-2-methylene-*N*-phenylbutanamide (**2a**) was obtained according to the preparation of **2a**. Lenalidomide (578.24 mg, 2.23 mmol, 1.0 equiv.) was used and the resulting residue was purified by flash column chromatography (2.5% MeOH/CH_2_Cl_2_) to give **2j** (502 mg, 1.34 mmol, 60%) as a yellow solid, m.p.: 188.5-189.8℃.

**TLC**: R_f_ = 0.50 (5% MeOH/CH_2_Cl_2_, KMnO_4_ stain).

**^1^H NMR** (400 MHz, CDCl_3_) *δ* 8.91 (s, 1H), 8.46 (s, 1H), 7.70 (dd, *J* = 11.3, 7.7 Hz, 2H), 7.50 (t, *J* = 7.7 Hz, 1H), 5.99 (s, 1H), 5.63 (s, 1H), 5.07 (dd, *J* = 13.3, 5.0 Hz, 1H), 4.38 (s, 2H), 3.74 (t, *J* = 6.4 Hz, 2H), 2.87 (t, *J* = 6.5 Hz, 2H), 2.83 – 2.62 (m, 2H), 2.27 (dt, *J* = 13.5, 6.7 Hz, 1H), 2.11 (d, *J* = 12.5 Hz, 1H).

**^13^C NMR** (101 MHz, CDCl_3_) δ 171.55, 170.27, 169.02, 166.39, 141.16, 135.03, 132.76, 132.57, 129.10, 126.95, 122.18, 121.40, 51.81, 46.67, 43.01, 35.64, 31.40, 23.09.

**HRMS** (ESI^+^) calcd for C_18_H_18_ClN_3_O_4_ [M+H]^+^ 376.1058, found 376.1052.

**2-(4-(3-(2-Chloro-10H-phenothiazin-10-yl) propyl) piperazin-1-yl) ethyl 4-chloro-2-methylenebutanoate** (**2k)**

2-(4-(3-(2-Chloro-10H-phenothiazin-10-yl) propyl) piperazin-1-yl) ethyl 4-chloro-2-methylenebutanoate (**2k**) was obtained according to the preparation of **2a**. Perphenazine (900.85 mg, 2.23 mmol, 1.0 equiv.) was used and the resulting residue was purified by flash column chromatography (2.5% MeOH/CH_2_Cl_2_) to give **2k** (810.16 mg, 1.56 mmol, 70%) as a yellow oil.

**TLC**: R_f_ = 0.50 (5% MeOH/CH_2_Cl_2_, KMnO_4_ stain).

**^1^H NMR** (400 MHz, CDCl_3_) *δ* 7.21 – 7.12 (m, 2H), 7.04 (d, *J* = 8.1 Hz, 1H), 6.97 (dd, *J* = 7.5, 1.2 Hz, 1H), 6.94 – 6.90 (m, 2H), 6.90 – 6.87 (m, 1H), 6.32 (s, 1H), 5.73 (s, 1H), 4.31 (t, *J* = 5.9 Hz, 2H), 3.93 (t, *J* = 6.8 Hz, 2H), 3.71 (t, *J* = 6.8 Hz, 2H), 2.79 (td, *J* = 6.8, 1.1 Hz, 2H), 2.70 (t, *J* = 5.9 Hz, 2H), 2.52 (dd, *J* = 18.0, 10.9 Hz, 10H), 1.97 (p, *J* = 7.0 Hz, 2H).

**^13^C NMR** (101 MHz, CDCl_3_) *δ* 166.21, 146.49, 144.51, 136.41, 133.22, 128.24, 127.87, 127.50, 127.41, 124.79, 123.52, 122.89, 122.23, 115.86, 115.82, 62.39, 56.46, 55.45, 53.27, 45.34, 42.81, 35.50, 24.20.

**HRMS** (ESI^+^) calcd for C_26_H_31_Cl_2_N_3_O_2_S [M+H]^+^ 520.1586, found 520.1594.

## 4.2. Synthesis of Styrene Derivatives

**(4-Chlorobut-1-en-2-yl) benzene (2l)**^8^

3-Phenylbut-3-en-1-ol (**S7**) was prepared following a modified literature procedure:^8^ But-3-yn-1-ol (**S4**) (0.70 g, 10 mmol, 1.0 equiv.), Pd (PPh_3_)_4_ (347 mg, 0.300 mmol, 3.00 mol%) and phenylboronic acid (1.34 g, 11.0 mmol, 1.10 equiv.) were placed in a thick-walled glass vessel under an argon atmosphere. Dry 1,4-dioxane (20 mL) and AcOH (120 mg, 2.00 mmol, 20.0 mol%) were added and the solution was stirred at r.t. for 15 min, then at 80 °C for 15 h. The reaction was cooled to r.t. and 1,4- dioxane was removed *in vacuo*. The resulting crude product was purified by flash column chromatography (20% EtOAc/hexane) to afford 3-phenylbut-3-en-1-ol (**S7**) (0.4 g, 2.6 mmol, 30%) as a yellow oil.

Homoallyl chloride **2l** was then prepared following the same chlorination procedure as described in the synthesis of **2e**, with alcohol **S7** (0.4 g, 2.6 mmol, 1.00 equiv.). The crude product was purified by flash column chromatography (pentane) to afford **2l** (200mg, 1.2 mmol, 44%) as a pale-yellow oil.

**TLC**: R_f_ = 0.85 (pentane, KMnO_4_ stain).

**^1^H NMR** (400 MHz, CDCl_3_) δ 7.41 – 7.27 (m, 5H), 5.39 (d, J = 1.1 Hz, 1H), 5.16 (t, J = 1.2 Hz, 1H), 3.58 (t, J = 7.3 Hz, 2H), 2.97 (td, J = 7.4, 1.2 Hz, 2H).

**LCMS** (ESI^+^) calcd. for C_10_H_11_Cl [M+H]^+^ 167.1, found 167.1.

**3-(4-Chlorobut-1-en-2-yl) aniline (2m)**^8^

3-(4-Chlorobut-1-en-2-yl) aniline **2m** was obtained according to the preparation of **2l.** (3-aminophenyl) boronic acid (0.5 g, 3.65 mmol, 1.10 equiv.) was used and the crude product was purified by flash column chromatography (pentane) to afford **2m** (91 mg, 0.50 mmol, 40%) as a pale-yellow oil.

**TLC**: R_f_ = 0.65 (5% EtOAc/hexane, KMnO_4_ stain).

**^1^H NMR** (400 MHz, CDCl_3_) *δ* 7.13 (t, *J* = 7.8 Hz, 1H), 6.78 (ddd, *J* = 7.7, 1.7, 0.9 Hz, 1H), 6.71 (t, *J* = 2.1 Hz, 1H), 6.63 (ddd, *J* = 7.9, 2.4, 1.0 Hz, 1H), 5.35 (d, *J* = 1.3 Hz, 1H), 5.12 (d, *J* = 1.3 Hz, 1H), 3.58 (t, *J* = 7.4 Hz, 2H), 3.24 (s, 2H), 2.93 (td, *J* = 7.4, 1.2 Hz, 2H).

**^13^C NMR** (101 MHz, CDCl_3_) *δ* 146.50, 144.85, 141.22, 129.45, 116.62, 114.73, 114.68, 112.99, 43.01, 38.88.

**HRMS** (ESI^+^) calcd for C_26_H_31_Cl_2_N_3_O_2_S [M+H] ^+^ 182.0731, found 182.0731.

**2-(4-Chlorobut-1-en-2-yl) benzofuran (2n)**^10^

3-(Benzofuran-2-yl) but-3-en-1-ol (**S10**) was prepared following a modified literature procedure:^9^ Under an argon atmosphere, 3-bromobut-3-en-1-ol (**S9)** (0.50 g, 3.31 mmol, 1.0 equiv.), Pd (PPh_3_)_4_ (190 mg, 0.166 mmol, 5.00 mol%), benzofuran-2-ylboronic acid (0.8 g, 4.97 mmol, 1.5 equiv.) were placed in a thick-walled glass vessel. Dry 1,4-dioxane (15 mL) and 3 M aqueous K_2_CO_3_ solution (3.0 mL, 7.95 mmol, 2.4 equiv.) were added, then the solution was stirred at 90 °C for 24 h. The reaction was quenched with H_2_O (10 mL) and extracted with EtOAc (3 × 20 mL). The organic phase was dried (Na_2_SO_4_), filtered, and concentrated *in vacuo*. The resulting crude product was purified by flash column chromatography (20% EtOAc/hexane) to afford 3-phenylbut-3-en-1-ol (**S10**) (0.53 g, 2.8 mmol, 85%) as a yellow oil.

Homoallyl chloride **2n** was prepared following a modified literature procedure:^10^ Et_3_N (0.57 g, 5.64 mmol, 2.0 equiv.) was added to 3-(benzofuran-2-yl) but-3-en-1-ol (**S10**) (0.53 g, 2.82 mmol, 1.00 equiv.) in CH_2_Cl_2_ (20 mL) at r.t., followed by DMAP (70 mg, 0.564 mmol, 20.0 mol%) and *p*-toluenesulfonyl chloride (0.65 g, 3.384 mmol, 1.20 equiv.), then the flask was evacuated and back filled with argon three times. The reaction was stirred for 20 h before quenching with saturated aqueous Na_2_CO_3_ (30 mL) and extracting with CH_2_Cl_2_ (3 × 30 mL). The organic phase was dried (Na_2_SO_4_), filtered, and concentrated *in vacuo*. Purification by flash column chromatography (20% EtOAc/hexane) afforded sulfonate **S11** (0.78 g, 2.3 mmol, 81.6%) as a yellow oil.

To a solution of sulfonate **S11** (0.78 g, 2.3 mmol, 1.00 equiv.) in acetone (15 mL) was added LiCl (0.5 g, 11.5 mmol, 5.00 equiv.) and the reaction mixture was heated to reflux for 20 h. The reaction was allowed to cool to r.t., quenched with H_2_O (30 mL), and extracted with CH_2_Cl_2_ (3 × 30 mL). The organic phase was dried (Na_2_SO_4_), filtered, and concentrated *in vacuo*. Purification by flash column chromatography (2% EtOAc/hexane) afforded **2n** (0.4 g, 3.87 mmol, 86%) as a colourless oil.

**TLC**: R_f_ = 0.5 (5% EtOAc/hexane, KMnO_4_ stain).

**^1^H NMR** (400 MHz, CDCl_3_) *δ* 7.55 (ddd, *J* = 7.7, 1.3, 0.7 Hz, 1H), 7.46 (dq, *J* = 8.4, 1.0 Hz, 1H), 7.29 (ddd, *J* = 8.3, 7.2, 1.4 Hz, 1H), 7.21 (td, *J* = 7.5, 1.0 Hz, 1H), 6.70 (d, *J* = 0.8 Hz, 1H), 5.95 (s, 1H), 5.30 (q, *J* = 1.2 Hz, 1H), 3.76 (t, *J* = 7.4 Hz, 2H), 2.95 (td, *J* = 7.4, 1.1 Hz, 2H).

**LCMS** (ESI^+^) calcd. for C_10_H_11_Cl [M+H] ^+^ 207.1, found 207.1.

## 4.2. Synthesis of Allyl Chlorides

**Methyl (*E*)-4-chlorobut-2-enoate (4a)**^12^

(*E*)-4-chlorobut-2-enoic acid **S13** was synthesized in two steps from but-3-enoic acid (**S12**) following a literature procedure.^11,12^ Under an argon atmosphere but-3-enoic acid (**S11**) (2.0 g, 23.2 mmol, 1.0 equiv.), PhSeCl (440 mg, 2.32 mmol, 1.00 mol%) were placed in a thick-walled glass vessel. A solution of *N*-chlorosuccinimide (3.4 g, 25.52 mmol, 1.1 equiv.) in dry MeCN (20 mL) were added and the solution was stirred at r.t. for 16h. The MeCN was removed *in vacuo*, then H_2_O was added and the mixture was extracted with EtOAc (3 × 20 mL). The combined organic phases were dried (MgSO_4_), filtered, and concentrated *in vacuo*. The resulting crude product was purified by flash column chromatography (30% EtOAc/hexane and 0.5% acetic acid) to afford (*E*)-4-chlorobut-2-enoic acid (**S13**) (2.7 g, 22.6 mmol, 96%) as a white solid.

Methyl (E)-4-chlorobut-2-enoate **4a** was prepared from (*E*)-4-chlorobut-2-enoic acid **S13** following a literature procedure.^12^ (*E*)-4-chlorobut-2-enoic acid (**S13**) (1.35 g, 11.2 mmol, 1.0 equiv.) and DMAP (0.14 g, 1.12 mmol, 0.1 equiv.) were dissolved in anhydrous CH_2_Cl_2_ (5.0 mL/mmol substrate) under N_2_ atmosphere. After 5 min stirring at room temperature, the mixture was cooled to 0 °C and subsequently MeOH (0.43 g, 13.44 mmol, 1.2 equiv.) and DIC (1.48 g, 11.76 mmol, 1.05 equiv.) were added. The reaction was slowly warmed to r.t. and stirring for additional 4 h. Up on completed, the solution was filtered over celite. The filtrate was washed with pentane and then the organic phase was dried and concentrated to a yellow oil. Flash column chromatography (2.5% EtOAc/hexane) yielded the Methyl (E)-4-chlorobut-2-enoate (**4a)** (0.44 g, 3.2 mmol, 29%) as a colorless oil.

**TLC:** R_f_ = 0.85 (10% EtOAc/hexane, KMnO_4_ stain).

**^1^H NMR** (400 MHz, CDCl_3_) *δ* 6.99 (dt, *J* = 15.4, 6.1 Hz, 1H), 6.11 (dt, *J* = 15.4, 1.6 Hz, 1H), 4.17 (dd, *J* = 6.1, 1.6 Hz, 2H), 3.76 (s, 3H).

**^13^C NMR** (101 MHz, Chloroform-d) *δ* 166.05, 141.97, 123.71, 51.79, 42.45.

**LCMS** (ESI^+^) calcd. for C_5_H_7_ClO_2_ [M+H] ^+^ 135.0, found 135.1.

**S-Butyl (*E*)-4-chlorobut-2-enethioate (4b)**

S-Butyl (*E*)-4-chlorobut-2-enethioate **4b** was obtained according to the preparation of **4a**. Butyl mercaptan (1.22 g, 13.44 mmol, 1.2 equiv.) was used. Purified by flash column chromatography (2.5% EtOAc/hexane) yielded the **4b** (0.44 g, 3.2 mmol, 29%) as a colorless oil.

**TLC:** R_f_ = 0.85 (10% EtOAc/hexane, KMnO_4_ stain).

**^1^H NMR** (400 MHz, CDCl_3_) *δ* 6.88 (dt, *J* = 15.2, 6.1 Hz, 1H), 6.36 (dt, *J* = 15.3, 1.6 Hz, 1H), 4.16 (dd, *J* = 6.0, 1.6 Hz, 2H), 2.97 (t, *J* = 7.3 Hz, 2H), 1.64 – 1.53 (m, 2H), 1.42 (dt, *J* = 14.8, 7.4 Hz, 2H), 0.93 (t, *J* = 7.3 Hz, 3H).

**^13^C NMR** (101 MHz, CDCl_3_) *δ* 189.42, 137.16, 130.61, 42.54, 31.50, 28.72, 21.97, 13.57.

**HRMS** (ESI^+^) calcd for C_8_H_13_ClOS [M+H] ^+^ 193.0448, found 193.0453.

# 5. Product Characterization

**Benzyl 4-((1-(phenylcarbamoyl) cyclopropyl) methyl) piperidine-1-carboxylate (3a)**

The title compound was prepared according to General Procedure B with 4CzTPN (4.0 mg, 5.0 µmol, 0.01 equiv.), NaOAc (82.03 mg, 1.0 mmol, 2.0 equiv.), 4-chloro-2-methylene-N-phenylbutanamide (104.83 mg, 0.5 mmol, 1.0 equiv.), NHC-1 (256.91 mg, 0.65 mmol, 1.3 equiv.), pyridine (63.28 mg, 0.8 mmol, 1.6 equiv.), benzyl 4-hydroxypiperidine-1-carboxylate (152.93 mg, 0.65 mmol, 1.3 equiv.).

Purification by flash column chromatography (20% EtOAc/pentane) gave the title compound (182.5 mg, 0.465 mmol, 93%) as a white solid, m.p.: 127.1-128.2℃.

**TLC:** R_f_ = 0.50 (30% EtOAc/pentane, KMnO_4_ stain).

**^1^H NMR** (400 MHz, CDCl_3_) δ 7.51 – 7.47 (m, 2H), 7.42 (s, 1H), 7.35 – 7.32 (m, 4H), 7.30 (d, *J* = 8.1 Hz, 2H), 7.12 – 7.07 (m, 1H), 5.10 (s, 2H), 4.14 (d, *J* = 14.2 Hz, 2H), 2.73 (s, 2H), 1.86 – 1.76 (m, 3H), 1.60 (d, *J* = 6.7 Hz, 2H), 1.20 (q, *J* = 4.4 Hz, 2H), 1.17 – 1.07 (m, 2H), 0.66 (q, *J* = 4.4 Hz, 2H).

**^13^C NMR** (126 MHz, CDCl_3_) *δ* 171.97, 155.25, 137.92, 136.88, 129.03, 128.50, 127.97, 127.84, 124.29, 119.96, 67.01, 44.14, 42.34, 35.45, 32.44, 24.26, 14.02.

**HRMS** (ESI^+^) calcd. for C_24_H_28_N_2_O_3_ [M+H] ^+^ 393.2172, found 393.2176.

**Benzyl 4-((1-carbamoylcyclopropyl) methyl) piperidine-1-carboxylate (3b)**

The title compound was prepared according to General Procedure B with 4CzTPN (4.0 mg, 5.0 µmol, 0.01 equiv.), NaOAc (82.03 mg, 1.0 mmol, 2.0 equiv.), 4-Chloro-2-methylenebutanamide (66.79 mg, 0.5 mmol, 1.0 equiv.), NHC-1 (256.91 mg, 0.65 mmol, 1.3 equiv.), pyridine (63.28 mg, 0.8 mmol, 1.6 equiv.), benzyl 4-hydroxypiperidine-1-carboxylate (152.93 mg, 0.65 mmol, 1.3 equiv.).

Purification by flash column chromatography (60% EtOAc/pentane) gave the title compound (110 mg, 0.35 mmol, 70%) as a colourless oil.

**TLC:** R_f_ = 0.50 (70% EtOAc/pentane, KMnO_4_ stain).

**^1^H NMR** (500 MHz, CDCl_3_) *δ* 7.35 (d, *J* = 5.6 Hz, 4H), 7.32 – 7.27 (m, 1H), 5.94 (d, *J* = 188.1 Hz, 2H), 5.11 (s, 2H), 4.16 (dd, *J* = 19.2, 10.1 Hz, 2H), 2.73 (s, 2H), 1.77 (ddd, *J* = 14.1, 6.6, 3.6 Hz, 3H), 1.49 (d, *J* = 6.8 Hz, 2H), 1.12 (q, *J* = 4.3 Hz, 4H), 0.62 (q, *J* = 4.1 Hz, 2H).

**^13^C NMR** (101 MHz, CDCl_3_) *δ* 177.11, 155.29, 136.92, 128.53, 128.00, 127.86, 67.03, 44.21, 41.84, 35.29, 32.48, 22.64, 14.57.

**HRMS** (ESI^+^) calcd for C_18_H_24_N_2_O_3_ [M+H] ^+^ 317.1860, found 317.1862.

**Benzyl 4-((1-((4-(methoxycarbonyl) phenyl) carbamoyl) cyclopropyl) methyl) piperidine-1-carboxylate (3c)**

The title compound was prepared according to General Procedure B with 4CzTPN (4.0 mg, 5.0 µmol, 0.01 equiv.), NaOAc (82.03 mg, 1.0 mmol, 2.0 equiv.), Methyl 4-(4-chloro-2-methylenebutanamido) benzoate (133.86 mg, 0.5 mmol, 1.0 equiv.), NHC-1 (256.91 mg, 0.65 mmol, 1.3 equiv.), pyridine (63.28 mg, 0.8 mmol, 1.6 equiv.), benzyl 4-hydroxypiperidine-1-carboxylate (152.93 mg, 0.65 mmol, 1.3 equiv.).

Purification by flash column chromatography (25% EtOAc/pentane) gave the title compound (180.2 mg, 0.40 mmol, 80%) as a yellow sticky oil.

**TLC:** R_f_ = 0.50 (40% EtOAc/pentane, KMnO_4_ stain).

**^1^H NMR** (400 MHz, CDCl_3_) *δ* 8.00 – 7.94 (m, 2H), 7.81 (s, 1H), 7.62 – 7.58 (m, 2H), 7.36 – 7.27 (m, 5H), 5.10 (s, 2H), 4.15 (d, *J* = 12.8 Hz, 2H), 3.88 (s, 3H), 2.73 (s, 2H), 1.77 (ddt, *J* = 14.4, 10.7, 3.3 Hz, 3H), 1.61 (d, *J* = 6.6 Hz, 2H), 1.22 (q, *J* = 4.4 Hz, 2H), 1.18 – 1.03 (m, 2H), 0.73 – 0.64 (m, 2H).

**^13^C NMR** (101 MHz, CDCl_3_) *δ* 172.41, 166.67, 155.27, 142.35, 136.85, 130.80, 128.52, 128.01, 127.81, 125.48, 119.09, 67.06, 52.07, 44.16, 42.15, 35.46, 32.41, 24.53, 14.17.

**HRMS** (ESI^+^) calcd for C_26_H_30_N_2_O_5_ [M+H] ^+^ 451.2228, found 451.2230.

**Benzyl 4-((1-((3-methoxyphenyl) carbamoyl) cyclopropyl) methyl) piperidine-1-carboxylate (3d)**

The title compound was prepared according to General Procedure B with 4CzTPN (4.0 mg, 5.0 µmol, 0.01 equiv.), NaOAc (82.03 mg, 1.0 mmol, 2.0 equiv.), 4-chloro-N-(3-methoxyphenyl)-2-methylenebutanamide (119.85 mg, 0.5 mmol, 1.0 equiv.), NHC-1 (256.91 mg, 0.65 mmol, 1.3 equiv.), pyridine (63.28 mg, 0.8 mmol, 1.6 equiv.), benzyl 4-hydroxypiperidine-1-carboxylate (152.93 mg, 0.65 mmol, 1.3 equiv.).

Purification by flash column chromatography (20% EtOAc/pentane) gave the title compound (200 mg, 0.475 mmol, 95%) as a yellow sticky oil.

**TLC:** R_f_ = 0.50 (30% EtOAc/pentane, KMnO_4_ stain).

**^1^H NMR** (400 MHz, CDCl_3_) *δ* 7.41 – 7.29 (m, 7H), 7.24 (t, *J* = 8.1 Hz, 1H), 6.96 (dd, *J* = 8.1, 2.0 Hz, 1H), 6.70 (dd, *J* = 8.3, 2.5 Hz, 1H), 5.14 (s, 2H), 4.20 (s, 2H), 3.83 (s, 3H), 2.77 (s, 2H), 1.84 (d, *J* = 13.0 Hz, 2H), 1.79 (ddd, *J* = 11.0, 7.0, 3.5 Hz, 2H), 1.64 (d, *J* = 6.7 Hz, 2H), 1.24 (q, *J* = 4.4 Hz, 2H), 1.19 (s, 1H), 0.71 (q, *J* = 4.4 Hz, 2H).

**^13^C NMR** (101 MHz, CDCl_3_) *δ* 171.93, 160.22, 155.23, 139.14, 136.87, 129.69, 128.49, 127.97, 127.86, 111.74, 110.22, 105.44, 67.01, 55.34, 44.12, 42.35, 35.44, 32.43, 29.72, 24.30, 14.05.

**HRMS** (ESI^+^) calcd for C_25_H_30_N_2_O_4_ [M+H] ^+^ 423.2278, found 423.2280.

**Benzyl 4-((1-(methoxycarbonyl) cyclopropyl) methyl) piperidine-1-carboxylate (3e)**

The title compound was prepared according to General Procedure B with 4CzTPN (4.0 mg, 5.0 µmol, 0.01 equiv.), NaOAc (82.03 mg, 1.0 mmol, 2.0 equiv.), Methyl 4-chloro-2-methylenebutanoate (74.25 mg, 0.5 mmol, 1.0 equiv.), NHC-1 (256.91 mg, 0.65 mmol, 1.3 equiv.), pyridine (63.28 mg, 0.8 mmol, 1.6 equiv.), benzyl 4-hydroxypiperidine-1-carboxylate (152.93 mg, 0.65 mmol, 1.3 equiv.).

Purification by flash column chromatography (10% EtOAc/pentane) gave the title compound (106.1 mg, 0.32 mmol, 64%) as a brown oil.

**TLC:** R_f_ = 0.50 (20% EtOAc/pentane, KMnO_4_ stain).

**^1^H NMR** (500 MHz, CDCl_3_) *δ* 7.35 (d, *J* = 4.4 Hz, 4H), 7.30 (q, *J* = 4.0 Hz, 1H), 5.11 (s, 2H), 4.14 (d, *J* = 21.5 Hz, 2H), 3.64 (s, 3H), 2.77 (d, *J* = 22.5 Hz, 2H), 1.82 (ddh, *J* = 11.0, 7.2, 3.4 Hz, 1H), 1.72 (d, *J* = 12.3 Hz, 2H), 1.49 (t, *J* = 6.5 Hz, 2H), 1.23 – 1.20 (m, 2H), 1.14 – 1.03 (m, 2H), 0.67 (q, *J* = 4.2 Hz, 2H).

**^13^C NMR** (101 MHz, CDCl_3_) *δ* 175.63, 155.31, 137.00, 128.51, 127.95, 127.86, 66.98, 51.81, 44.29, 40.76, 35.15, 31.49, 21.44, 15.70.

**HRMS** (ESI^+^) calcd for C_19_H_25_NO_4_ [M+H] ^+^ 332.1856, found 332.1860.

**Benzyl 4-((1-((benzyloxy) carbonyl) cyclopropyl) methyl) piperidine-1-carboxylate (3f)**

The title compound was prepared according to General Procedure B with 4CzTPN (4.0 mg, 5.0 µmol, 0.01 equiv.), NaOAc (82.03 mg, 1.0 mmol, 2.0 equiv.), benzyl 4-chloro-2-methylenebutanoate (112.34 mg, 0.5 mmol, 1.0 equiv.), NHC-1 (256.91 mg, 0.65 mmol, 1.3 equiv.), pyridine (63.28 mg, 0.8 mmol, 1.6 equiv.), benzyl 4-hydroxypiperidine-1-carboxylate (152.93 mg, 0.65 mmol, 1.3 equiv.).

Purification by flash column chromatography (5% EtOAc/pentane) gave the title compound (179.3 mg, 0.44 mmol, 88%) as a yellow sticky oil.

**TLC:** R_f_ = 0.50 (15% EtOAc/pentane, KMnO_4_ stain).

**^1^H NMR** (400 MHz, CDCl_3_) *δ* 7.41 – 7.26 (m, 10H), 5.10 (d, *J* = 6.8 Hz, 4H), 4.12 (s, 2H), 2.68 (s, 2H), 1.92 – 1.76 (m, 1H), 1.68 (d, *J* = 12.9 Hz, 2H), 1.56 – 1.45 (m, 2H), 1.25 (q, *J* = 3.9 Hz, 2H), 1.06 (d, *J* = 13.3 Hz, 2H), 0.68 (t, *J* = 3.5 Hz, 2H).

**^13^C NMR** (126 MHz, CDCl_3_) *δ* 174.87, 155.26, 137.01, 136.17, 128.58, 128.50, 128.20, 128.02, 127.94, 127.85, 66.95, 66.25, 44.26, 40.72, 35.16, 21.61, 15.74.

**HRMS** (ESI^+^) calcd for C_25_H_29_NO_4_ [M+H] ^+^ 408.2169, found 408.2163.

**Benzyl 4-((1-(4,4,5,5-tetramethyl-1,3,2-dioxaborolan-2-yl) cyclopropyl) methyl) piperidine-1-carboxylate (3g)**

The title compound was prepared according to General Procedure B with 4CzTPN (4.0 mg, 5.0 µmol, 0.01 equiv.), NaOAc (82.03 mg, 1.0 mmol, 2.0 equiv.), 2-(4-Chlorobut-1-en-2-yl)-4,4,5,5-tetramethyl-1,3,2-dioxaborolane (108.25 mg, 0.5 mmol, 1.0 equiv.), NHC-1 (256.91 mg, 0.65 mmol, 1.3 equiv.), pyridine (63.28 mg, 0.8 mmol, 1.6 equiv.), benzyl 4-hydroxypiperidine-1-carboxylate (152.93 mg, 0.65 mmol, 1.3 equiv.).

Purification by flash column chromatography (5% EtOAc/pentane) gave the title compound (99.8 mg, 0.25 mmol, 50%) as a yellow sticky oil.

**TLC:** R_f_ = 0.50 (15% EtOAc/pentane, KMnO_4_ stain).

**^1^H NMR** (500 MHz, CDCl_3_) *δ* 7.39 – 7.25 (m, 5H), 5.12 (s, 2H), 4.12 (d, *J* = 23.3 Hz, 2H), 2.74 (s, 2H), 1.73 (s, 1H), 1.71 – 1.62 (m, 2H), 1.27 – 1.21 (m, 2H), 1.19 (s, 12H), 1.14 – 1.03 (m, 2H), 0.67 (q, *J* = 3.4 Hz, 2H), 0.27 (q, *J* = 3.4 Hz, 2H).

**^13^C NMR** (101 MHz, CDCl_3_) *δ* 155.39, 137.12, 128.50, 127.91, 127.83, 82.98, 66.91, 44.45, 43.10, 36.50, 31.50, 30.25, 24.61, 11.80.

**HRMS** (ESI^+^) calcd for C_23_H_34_BNO_4_ [M+H] ^+^ 400.2654, found 400.2656.

**Benzyl 4-((1-(dibutoxyphosphoryl) cyclopropyl) methyl) piperidine-1-carboxylate (3h)**

The title compound was prepared according to General Procedure B with 4CzTPN (4.0 mg, 5.0 µmol, 0.01 equiv.), NaOAc (82.03 mg, 1.0 mmol, 2.0 equiv.), Dibutyl (4-chlorobut-1-en-2-yl) phosphonate (141.3 mg, 0.5 mmol, 1.0 equiv.), NHC-1 (256.91 mg, 0.65 mmol, 1.3 equiv.), pyridine (63.28 mg, 0.8 mmol, 1.6 equiv.), benzyl 4-hydroxypiperidine-1-carboxylate (152.93 mg, 0.65 mmol, 1.3 equiv.).

Purification by flash column chromatography (15% EtOAc/pentane) gave the title compound (155.9 mg, 0.335 mmol, 67%) as a yellow oil.

**TLC:** R_f_ = 0.50 (30% EtOAc/pentane, KMnO_4_ stain).

**^1^H NMR** (400 MHz, CDCl_3_) *δ* 7.39 – 7.28 (m, 5H), 5.12 (s, 2H), 4.17 (s, 2H), 4.02 (q, *J* = 6.6 Hz, 4H), 3.17 (s, 1H), 2.73 (d, *J* = 15.9 Hz, 2H), 1.93 (ddp, *J* = 11.4, 7.6, 3.9 Hz, 1H), 1.84 (s, 2H), 1.64 (dq, *J* = 8.6, 6.6 Hz, 4H), 1.44 – 1.34 (m, 6H), 1.09 (dt, *J* = 15.6, 3.1 Hz, 2H), 1.04 – 0.98 (m, 1H), 0.94 (t, *J* = 7.4 Hz, 7H), 0.52 (dt, *J* = 10.5, 3.3 Hz, 2H).

**^13^C NMR** (101 MHz, CDCl_3_) *δ* 155.33, 137.00, 128.48, 127.94, 127.85, 66.98, 65.78, 65.71, 44.24, 42.13, 42.07, 35.06, 32.70, 32.64, 18.84, 13.64, 13.18, 11.28, 10.85.

**^31^P NMR** (162 MHz, DMSO-d6) *δ* 31.67.

**HRMS** (ESI^+^) calcd for C_25_H_40_NO_5_P [M+H] ^+^ 466.2717, found 466.2719.

**1-((1-((benzyloxy) carbonyl) piperidin-4-yl) methyl) cyclopropane-1-carboxylic acid (3i)**

The title compound was prepared according to General Procedure B with 4CzTPN (4.0 mg, 5.0 µmol, 0.01 equiv.), NaOAc (82.03 mg, 1.0 mmol, 2.0 equiv.), 4-chloro-2-methylenebutanoic acid (67.28 mg, 0.5 mmol, 1.0 equiv.), NHC-1 (256.91 mg, 0.65 mmol, 1.3 equiv.), pyridine (63.28 mg, 0.8 mmol, 1.6 equiv.), benzyl 4-hydroxypiperidine-1-carboxylate (152.93 mg, 0.65 mmol, 1.3 equiv.).

Purification by flash column chromatography (20% EtOAc/pentane + 0.5% AcOH) gave the title compound (106.33 mg, 0.335 mmol, 67%) as a yellow oil.

**TLC:** R_f_ = 0.50 (40% EtOAc/pentane + 0.5% AcOH, KMnO_4_ stain).

**^1^H NMR** (400 MHz, CDCl_3_) *δ* 7.39 – 7.29 (m, 5H), 5.12 (s, 2H), 4.34 (td, *J* = 8.8, 2.8 Hz, 1H), 4.18 (td, *J* = 9.4, 6.6 Hz, 3H), 2.78 (s, 2H), 2.58 (qd, *J* = 9.3, 5.4 Hz, 1H), 2.40 (dddd, *J* = 11.9, 9.0, 6.6, 2.8 Hz, 1H), 1.95 – 1.79 (m, 2H), 1.70 (d, *J* = 13.7 Hz, 2H), 1.37 (ddd, *J* = 13.8, 9.3, 6.0 Hz, 1H), 1.17 (pd, *J* = 16.3, 14.3, 8.1 Hz, 2H).

**^13^C NMR** (101 MHz, CDCl_3_) *δ* 179.43, 155.29, 136.94, 128.51, 127.99, 127.86, 67.05, 66.40, 44.06, 44.04, 37.33, 36.60, 33.91, 32.31, 31.45, 29.43.

**HRMS** (ESI^+^) calcd for C_18_H_23_NO_4_ [M+H] ^+^ 318.1700, found 318.1702.

**Benzyl 4-((1-phenylcyclopropyl) methyl) piperidine-1-carboxylate (3j)**

The title compound was prepared according to General Procedure B with 4CzTPN (4.0 mg, 5.0 µmol, 0.01 equiv.), NaOAc (82.03 mg, 1.0 mmol, 2.0 equiv.), (4-Chlorobut-1-en-2-yl) benzene (83.33 mg, 0.5 mmol, 1.0 equiv.), NHC-1 (256.91 mg, 0.65 mmol, 1.3 equiv.), pyridine (63.28 mg, 0.8 mmol, 1.6 equiv.), benzyl 4-hydroxypiperidine-1-carboxylate (152.93 mg, 0.65 mmol, 1.3 equiv.).

Purification by flash column chromatography (2% EtOAc/pentane) gave the title compound (134.55 mg, 0.385 mmol, 77%) as a yellow oil.

**TLC:** R_f_ = 0.50 (10% EtOAc/pentane, KMnO_4_ stain).

**^1^H NMR** (400 MHz, CDCl_3_) *δ* 7.38 – 7.33 (m, 4H), 7.33 – 7.23 (m, 5H), 7.20 – 7.15 (m, 1H), 5.12 (d, *J* = 11.3 Hz, 2H), 4.09 (s, 2H), 2.63 (t, *J* = 13.0 Hz, 2H), 1.70 (s, 2H), 1.61 – 1.47 (m, 3H), 1.36 (dtq, *J* = 10.7, 7.0, 3.4 Hz, 1H), 1.07 (d, *J* = 12.7 Hz, 1H), 0.82 (t, *J* = 2.9 Hz, 2H), 0.63 (t, *J* = 2.9 Hz, 2H).

**^13^C NMR** (101 MHz, CDCl_3_) *δ* 155.31, 145.09, 137.10, 128.85, 128.51, 128.30, 127.93, 127.85, 126.03, 66.94, 47.19, 44.95, 44.19, 34.50, 32.54, 24.45, 23.52, 12.93.

**HRMS** (ESI^+^) calcd for C_23_H_27_NO_2_ [M+H] ^+^ 350.2115, found 350.2116.

**Benzyl 4-((1-(3-aminophenyl) cyclopropyl) methyl) piperidine-1-carboxylate (3k)**

The title compound was prepared according to General Procedure B with 4CzTPN (4.0 mg, 5.0 µmol, 0.01 equiv.), NaOAc (82.03 mg, 1.0 mmol, 2.0 equiv.), 3-(4-chlorobut-1-en-2-yl) aniline (90.5 mg, 0.5 mmol, 1.0 equiv.), NHC-1 (256.91 mg, 0.65 mmol, 1.3 equiv.), pyridine (63.28 mg, 0.8 mmol, 1.6 equiv.), benzyl 4-hydroxypiperidine-1-carboxylate (152.93 mg, 0.65 mmol, 1.3 equiv.).

Purification by flash column chromatography (15% EtOAc/pentane) gave the title compound (109.2 mg, 0.30 mmol, 60%) as a yellow oil.

**TLC:** R_f_ = 0.50 (40% EtOAc/pentane, KMnO_4_ stain).

**^1^H NMR** (400 MHz, CDCl_3_) *δ* 7.38 – 7.28 (m, 5H), 7.06 (t, *J* = 7.8 Hz, 1H), 6.70 (d, *J* = 7.7 Hz, 1H), 6.64 (t, *J* = 2.0 Hz, 1H), 6.52 (d, *J* = 10.2 Hz, 1H), 5.10 (s, 2H), 4.08 (s, 2H), 2.64 (s, 2H), 1.71 (s, 2H), 1.50 (d, *J* = 5.7 Hz, 2H), 1.27 (d, *J* = 11.7 Hz, 3H), 1.06 (s, 2H), 0.77 (t, *J* = 3.0 Hz, 2H), 0.58 (t, *J* = 2.8 Hz, 2H).

**^13^C NMR** (101 MHz, CDCl_3_) *δ* 155.31, 146.34, 146.18, 137.06, 129.13, 128.48, 127.91, 127.82, 119.28, 115.65, 112.99, 66.92, 47.10, 44.18, 34.43, 32.53, 29.74, 23.39, 12.87.

**HRMS** (ESI^+^) calcd. for C_23_H_27_NO_2_ [M+H] ^+^ 365.2234, found 365.2243.

**Benzyl 4-((1-phenylcyclopropyl) methyl) piperidine-1-carboxylate (3l)**

The title compound was prepared according to General Procedure B with 4CzTPN (4.0 mg, 5.0 µmol, 0.01 equiv.), NaOAc (82.03 mg, 1.0 mmol, 2.0 equiv.), 2-(4-Chlorobut-1-en-2-yl) benzofuran (103.1 mg, 0.5 mmol, 1.0 equiv.), NHC-1 (256.91 mg, 0.65 mmol, 1.3 equiv.), pyridine (63.28 mg, 0.8 mmol, 1.6 equiv.), benzyl 4-hydroxypiperidine-1-carboxylate (152.93 mg, 0.65 mmol, 1.3 equiv.).

Purification by flash column chromatography (10% EtOAc/pentane) gave the title compound (136.15 mg, 0.35 mmol, 70%) as a yellow oil.

**TLC:** R_f_ = 0.50 (20% EtOAc/pentane, KMnO_4_ stain).

**^1^H NMR** (400 MHz, CDCl_3_) *δ* 7.49 – 7.45 (m, 1H), 7.43 – 7.39 (m, 1H), 7.35 (s, 4H), 7.33 – 7.29 (m, 1H), 7.21 (qd, *J* = 7.0, 1.5 Hz, 2H), 6.35 (d, *J* = 0.9 Hz, 1H), 5.11 (s, 2H), 4.13 (s, 2H), 2.68 (s, 2H), 1.78 (s, 2H), 1.47 (d, *J* = 17.2 Hz, 1H), 1.30 (d, *J* = 22.3 Hz, 2H), 1.18 (s, 2H), 1.08 (q, *J* = 4.2 Hz, 2H), 0.78 (t, *J* = 3.2 Hz, 2H).

**^13^C NMR** (101 MHz, CDCl_3_) *δ* 162.08, 155.30, 154.46, 137.03, 128.90, 128.51, 127.95, 127.86, 123.27, 122.58, 120.22, 110.82, 101.18, 66.98, 44.22, 43.16, 35.00, 32.42, 31.42, 29.98, 29.76, 17.71, 13.98.

**HRMS** (ESI^+^) calcd. for C_23_H_27_NO_2_ [M+H] ^+^ 390.2064, found 390.2064.

**Benzyl 4-(1-(methoxycarbonyl) cyclopropyl) piperidine-1-carboxylate (5a)**

The title compound was prepared according to General Procedure B with 4CzTPN (4.0 mg, 5.0 µmol, 0.01 equiv.), NaOAc (82.03 mg, 1.0 mmol, 2.0 equiv.), Methyl-4-chlorobut-2-enoate (67.28 mg, 0.5 mmol, 1.0 equiv.), NHC-1 (256.91 mg, 0.65 mmol, 1.3 equiv.), pyridine (63.28 mg, 0.8 mmol, 1.6 equiv.), benzyl 4-hydroxypiperidine-1-carboxylate (152.93 mg, 0.65 mmol, 1.3 equiv.).

Purification by flash column chromatography (5% EtOAc/pentane) gave the title compound (115.85 mg, 0.365 mmol, 73%) as a yellow oil.

**TLC:** R_f_ = 0.50 (15% EtOAc/pentane, KMnO_4_ stain).

**^1^H NMR** (400 MHz, CDCl_3_) *δ* 7.39 – 7.28 (m, 5H), 5.12 (s, 2H), 4.16 (s, 2H), 3.66 (s, 3H), 2.73 (t, *J* = 12.8 Hz, 2H), 1.67 (s, 2H), 1.45 (dq, *J* = 8.4, 4.5 Hz, 1H), 1.38 – 1.32 (m, 1H), 1.29 – 1.22 (m, 2H), 1.16 (dt, *J* = 8.9, 4.4 Hz, 1H), 0.88 (dtt, *J* = 11.5, 8.2, 4.0 Hz, 1H), 0.76 (ddd, *J* = 8.3, 6.3, 4.2 Hz, 1H)..

**^13^C NMR** (101 MHz, CDCl_3_) *δ* 174.59, 155.32, 136.97, 128.52, 127.99, 127.89, 67.08, 51.75, 44.00, 43.98, 39.79, 28.02, 18.70, 13.99.

**HRMS** (ESI^+^) calcd for C_18_H_23_NO_4_ [M+H] ^+^ 318.1700, found 318.1702.

**Benzyl 4-(1-((butylthio) carbonyl) cyclopropyl) piperidine-1-carboxylate (5b)**

The title compound was prepared according to General Procedure B with 4CzTPN (4.0 mg, 5.0 µmol, 0.01 equiv.), NaOAc (82.03 mg, 1.0 mmol, 2.0 equiv.), S-Butyl-4-chlorobut-2-enethioate (96.35 mg, 0.5 mmol, 1.0 equiv.), NHC-1 (256.91 mg, 0.65 mmol, 1.3 equiv.), pyridine (63.28 mg, 0.8 mmol, 1.6 equiv.), benzyl 4-hydroxypiperidine-1-carboxylate (152.93 mg, 0.65 mmol, 1.3 equiv.).

Purification by flash column chromatography (15% EtOAc/pentane) gave the title compound (142.63 mg, 0.39 mmol, 78%) as a yellow oil.

**TLC:** R_f_ = 0.50 (30% EtOAc/pentane, KMnO_4_ stain).

**^1^H NMR** (400 MHz, CDCl_3_) *δ* 7.39 – 7.27 (m, 5H), 5.12 (s, 2H), 4.16 (s, 2H), 2.93 – 2.81 (m, 2H), 2.79 – 2.65 (m, 2H), 1.83 (dt, *J* = 8.4, 4.4 Hz, 1H), 1.72 (d, *J* = 8.7 Hz, 2H), 1.59 – 1.50 (m, 2H), 1.45 – 1.38 (m, 2H), 1.38 – 1.35 (m, 2H), 1.35 – 1.30 (m, 2H), 1.27 (d, *J* = 11.4 Hz, 2H), 0.95 – 0.90 (m, 3H), 0.90 – 0.80 (m, 2H).

**^13^C NMR** (101 MHz, CDCl_3_) *δ* 198.41, 155.30, 136.97, 128.52, 127.99, 127.88, 67.08, 43.97, 39.86, 31.72, 30.28, 28.79, 28.69, 22.01, 16.05, 13.61.

**HRMS** (ESI^+^) calcd for C_21_H_29_NO_3_S [M+H] ^+^ 376.1941, found 376.1944.

**Benzyl 4-(1-((butylthio) carbonyl) cyclopropyl) piperidine-1-carboxylate (5c)**

The title compound was prepared according to General Procedure A with 4CzTPN (4.0 mg, 5.0 µmol, 0.01 equiv.), NaOAc (82.03 mg, 1.0 mmol, 2.0 equiv.), S-Butyl-4-chlorobut-2-enethioate (96.35 mg, 0.5 mmol, 1.0 equiv.), NHC-2 (276.42 mg, 0.65 mmol, 1.3 equiv.), pyridine (63.28 mg, 0.8 mmol, 1.6 equiv.), tert-butyl (2-hydroxyethyl) carbamate (104.78 mg, 0.65 mmol, 1.3 equiv.).

Purification by flash column chromatography (2.5% EtOAc/pentane) gave the title compound (117.39 mg, 0.39 mmol, 78%) as a yellow oil.

**TLC:** R_f_ = 0.50 (5% EtOAc/pentane, KMnO_4_ stain).

**^1^H NMR** (400 MHz, CDCl_3_) *δ* δ 4.61 (s, 1H), 3.61 (d, *J* = 4.6 Hz, 1H), 3.18 (d, *J* = 5.7 Hz, 2H), 2.88 (td, *J* = 7.3, 2.1 Hz, 2H), 1.80 (dt, *J* = 8.1, 3.8 Hz, 1H), 1.59 – 1.48 (m, 4H), 1.44 (s, 9H), 1.41 – 1.36 (m, 2H), 1.36 – 1.33 (m, 1H), 0.91 (td, *J* = 7.3, 2.3 Hz, 3H), 0.81 (ddd, *J* = 8.0, 5.6, 4.0 Hz, 1H).

**^13^C NMR** (101 MHz, CDCl_3_) *δ* 198.47, 155.85, 47.78, 45.84, 34.82, 31.67, 31.52, 29.73, 28.75, 28.65, 28.40, 22.51, 21.96, 21.93, 17.12, 13.56, 13.54.

**HRMS** (ESI^+^) calcd. for C_21_H_29_NO_3_S [M+H] ^+^ 302.1785, found 302.1798

**Benzyl 4-(1-((butylthio) carbonyl) cyclopropyl) piperidine-1-carboxylate (5d)**

The title compound was prepared according to General Procedure C with 4CzTPN (4.0 mg, 5.0 µmol, 0.01 equiv.), NaOAc (82.03 mg, 1.0 mmol, 2.0 equiv.), S-Butyl-4-chlorobut-2-enethioate (96.35 mg, 0.5 mmol, 1.0 equiv.), NHC-3 (301.1 mg, 0.65 mmol, 1.3 equiv.), pyridine (63.28 mg, 0.8 mmol, 1.6 equiv.), tert-butyl 3-hydroxy-3-methylazetidine-1-carboxylate (121.71 mg, 0.65 mmol, 1.3 equiv.).

Purification by flash column chromatography (1.5% EtOAc/pentane) gave the title compound (111.18 mg, 0.34 mmol, 67%) as a yellow oil.

**TLC:** R_f_ = 0.50 (3% EtOAc/pentane, KMnO_4_ stain).

**^1^H NMR** (400 MHz, CDCl_3_) *δ* 3.53 (t, *J* = 7.4 Hz, 2H), 2.91 – 2.87 (m, 1H), 1.91 (dt, *J* = 8.6, 4.6 Hz, 1H), 1.72 (ddd, *J* = 9.1, 6.8, 4.4 Hz, 1H), 1.57 (d, *J* = 7.8 Hz, 4H), 1.53 (d, *J* = 7.5 Hz, 1H), 1.44 (s, 9H), 1.40 (dd, *J* = 7.1, 5.1 Hz, 2H), 1.38 – 1.32 (m, 2H), 1.26 (s, 3H), 0.92 (t, *J* = 7.3 Hz, 3H).

**^13^C NMR** (101 MHz, CDCl_3_) *δ* 198.31, 143.92, 79.60, 34.47, 33.60, 31.66, 31.45, 31.34, 29.81, 28.79, 28.43, 26.67, 24.80, 22.01, 13.86, 13.63.

**HRMS** (ESI^+^) calcd. for C_21_H_29_NO_3_S [M+H] ^+^ 328.1470, found 328.1463.

***tert*-Butyl 4-(3-(1-(phenylcarbamoyl) cyclopropyl) propyl) piperidine-1-carboxylate (6a)**

The title compound was prepared according to General Procedure A with 4CzTPN (4.0 mg, 5.0 µmol, 0.01 equiv.), NaOAc (82.03 mg, 1.0 mmol, 2.0 equiv.), 4-chloro-2-methylene-N-phenylbutanamide (104.83 mg, 0.5 mmol, 1.0 equiv.), NHC-2 (276.42 mg, 0.65 mmol, 1.3 equiv.), pyridine (63.28 mg, 0.8 mmol, 1.6 equiv.), tert-butyl 4-(2-hydroxyethyl) piperidine-1-carboxylate (149.06 mg, 0.65 mmol, 1.3 equiv.).

Purification by flash column chromatography (5% EtOAc/pentane) gave the title compound (158.48 mg, 0.41 mmol, 82%) as a brown oil.

**TLC:** R_f_ = 0.50 (15% EtOAc/pentane, KMnO_4_ stain).

**^1^H NMR** (400 MHz, CDCl_3_) *δ* 7.66 – 7.59 (m, 1H), 7.55 – 7.47 (m, 2H), 7.33 – 7.26 (m, 2H), 7.08 (t, *J* = 7.4 Hz, 1H), 4.08 (d, *J* = 13.8 Hz, 2H), 2.63 (d, *J* = 17.4 Hz, 2H), 1.68 – 1.59 (m, 4H), 1.53 (dt, *J* = 11.0, 6.7 Hz, 2H), 1.46 (s, 9H), 1.41 – 1.33 (m, 1H), 1.32 – 1.24 (m, 2H), 1.20 (q, *J* = 4.2 Hz, 2H), 1.05 (qd, *J* = 12.5, 4.5 Hz, 2H), 0.67 (q, *J* = 4.3 Hz, 1H).

**^13^C NMR** (101 MHz, CDCl_3_) *δ* 172.43, 154.91, 138.15, 128.91, 124.13, 120.13, 79.25, 36.65, 35.96, 35.22, 32.18, 28.51, 25.88, 24.82, 24.77, 14.32.

**HRMS** (ESI^+^) calcd for C_23_H_34_N_2_O_3_ [M+H] ^+^ 387.2642, found 387.2645.

***tert*-Butyl (3-(1-(phenylcarbamoyl) cyclopropyl) propyl) carbamate (6b)**

The title compound was prepared according to General Procedure A with 4CzTPN (4.0 mg, 5.0 µmol, 0.01 equiv.), NaOAc (82.03 mg, 1.0 mmol, 2.0 equiv.), 4-chloro-2-methylene-N-phenylbutanamide (104.83 mg, 0.5 mmol, 1.0 equiv.), NHC-2 (276.42 mg, 0.65 mmol, 1.3 equiv.), pyridine (63.28 mg, 0.8 mmol, 1.6 equiv.), tert-butyl (2-hydroxyethyl) carbamate (104.78 mg, 0.65 mmol, 1.3 equiv.).

Purification by flash column chromatography (15% EtOAc/pentane) gave the title compound (135.33 mg, 0.425 mmol, 85%) as a yellow oil.

**TLC:** R_f_ = 0.50 (40% EtOAc/pentane, KMnO_4_ stain).

**^1^H NMR** (400 MHz, CDCl_3_) *δ* 7.70 (s, 1H), 7.54 – 7.49 (m, 2H), 7.32 – 7.26 (m, 2H), 7.11 – 7.05 (m, 1H), 4.84 – 4.71 (m, 1H), 3.14 (q, *J* = 6.0 Hz, 2H), 1.74 – 1.63 (m, 4H), 1.43 (s, 10H), 1.24 – 1.18 (m, 2H), 0.70 – 0.64 (m, 2H).

**^13^C NMR** (101 MHz, CDCl_3_) *δ* 172.32, 156.33, 138.11, 128.90, 124.22, 120.29, 40.41, 31.85, 28.46, 28.21, 25.43, 14.66.

**HRMS** (ESI^+^) calcd for C_18_H_26_N_2_O_3_ [M+H] ^+^ 319.2016, found 319.2018.

**Methyl (S)-2-(((benzyloxy) carbonyl) amino)-4-(1-(phenylcarbamoyl) cyclopropyl) butanoate (6c)**

The title compound was prepared according to General Procedure A with 4CzTPN (4.0 mg, 5.0 µmol, 0.01 equiv.), NaOAc (82.03 mg, 1.0 mmol, 2.0 equiv.), 4-chloro-2-methylene-N-phenylbutanamide (104.83 mg, 0.5 mmol, 1.0 equiv.), NHC-2 (276.42 mg, 0.65 mmol, 1.3 equiv.), pyridine (63.28 mg, 0.8 mmol, 1.6 equiv.), methyl ((benzyloxy)carbonyl)-L-serinate (164.61 mg, 0.65 mmol, 1.3 equiv.).

Purification by flash column chromatography (20% EtOAc/pentane) gave the title compound (131.35 mg, 0.32 mmol, 64%) as a colorless oil.

**TLC:** R_f_ = 0.50 (40% EtOAc/pentane, KMnO_4_ stain).

**^1^H NMR** (400 MHz, CDCl_3_) *δ* 7.84 (s, 1H), 7.57 (d, *J* = 8.0 Hz, 2H), 7.43 – 7.26 (m, 8H), 7.11 (t, *J* = 7.4 Hz, 1H), 5.14 (s, 2H), 4.40 (qd, *J* = 7.2, 3.0 Hz, 1H), 3.77 (s, 3H), 2.03 – 1.85 (m, 2H), 1.72 (t, *J* = 8.3 Hz, 2H), 1.27 (q, *J* = 4.3 Hz, 2H), 0.69 (q, *J* = 4.0 Hz, 2H).

**^13^C NMR** (101 MHz, CDCl3) *δ* 172.75, 172.02, 156.16, 138.16, 136.10, 128.93, 128.63, 128.36, 128.18, 124.26, 120.29, 67.26, 66.54, 53.72, 53.55, 52.80, 52.66, 38.71, 30.51, 24.93, 15.19, 15.06.

**HRMS** (ESI^+^) calcd for C_23_H_26_N_2_O_5_ [M+H] ^+^ 411.1915, found 411.1917.

**1-(2-(3,5-Dimethyl-1*H*-pyrazol-1-yl)ethyl)-*N*-phenylcyclopropane-1-carboxamide (6d)**

The title compound was prepared according to General Procedure A with 4CzTPN (4.0 mg, 5.0 µmol, 0.01 equiv.), NaOAc (82.03 mg, 1.0 mmol, 2.0 equiv.), 4-chloro-2-methylene-N-phenylbutanamide (104.83 mg, 0.5 mmol, 1.0 equiv.), NHC-2 (276.42 mg, 0.65 mmol, 1.3 equiv.), pyridine (63.28 mg, 0.8 mmol, 1.6 equiv.), (3,5-dimethyl-1H-pyrazol-1-yl) methanol (82.0 mg, 0.65 mmol, 1.3 equiv.).

Purification by flash column chromatography (10% EtOAc/pentane) gave the title compound (110.52 mg, 0.39 mmol, 78%) as a yellow oil.

**TLC:** R_f_ = 0.50 (30% EtOAc/pentane, KMnO_4_ stain).

**^1^H NMR** (400 MHz, CDCl_3_) *δ* 9.13 (s, 1H), 7.61 – 7.55 (m, 2H), 7.28 (dd, *J* = 8.6, 7.4 Hz, 2H), 7.06 (td, *J* = 7.4, 1.2 Hz, 1H), 5.74 (s, 1H), 4.15 (t, *J* = 6.4 Hz, 2H), 2.20 (d, *J* = 4.0 Hz, 6H), 2.10 (t, *J* = 6.4 Hz, 2H), 1.20 – 1.14 (m, 2H), 0.52 – 0.46 (m, 2H).

**^13^C NMR** (101 MHz, CDCl3) *δ* 171.68, 147.76, 139.42, 138.72, 128.78, 123.92, 120.13, 105.38, 46.39, 35.56, 24.21, 14.24, 13.45, 10.94.

**HRMS** (ESI^+^) calcd for C_17_H_21_N_3_O [M+H] ^+^ 284.1757, found 284.1755.

**Methyl 4-(1-(3-ethoxy-3-oxopropyl) cyclopropane-1-carboxamido) benzoate (6e)**

The title compound was prepared according to General Procedure A with 4CzTPN (4.0 mg, 5.0 µmol, 0.01 equiv.), NaOAc (82.03 mg, 1.0 mmol, 2.0 equiv.), Methyl 4-(4-chloro-2-methylenebutanamido) benzoate (133.86 mg, 0.5 mmol, 1.0 equiv.), NHC-2 (276.42 mg, 0.65 mmol, 1.3 equiv.), pyridine (63.28 mg, 0.8 mmol, 1.6 equiv.), ethyl 2-hydroxyacetate (67.67 mg, 0.65 mmol, 1.3 equiv.).

Purification by flash column chromatography (10% EtOAc/pentane) gave the title compound (116.53 mg, 0.365 mmol, 73%) as a colorless oil.

**TLC:** R_f_ = 0.50 (30% EtOAc/pentane, KMnO_4_ stain).

**^1^H NMR** (400 MHz, CDCl_3_) *δ* 8.72 (s, 1H), 8.04 – 7.99 (m, 2H), 7.74 – 7.69 (m, 2H), 4.20 (q, *J* = 7.2 Hz, 2H), 3.92 (s, 3H), 2.60 (t, *J* = 6.8 Hz, 2H), 2.07 (t, *J* = 6.9 Hz, 2H), 1.37 – 1.32 (m, 2H), 1.30 – 1.26 (m, 3H), 0.76 (q, *J* = 4.2 Hz, 2H).

**^13^C NMR** (101 MHz, CDCl_3_) *δ* 174.77, 172.18, 166.78, 142.69, 130.77, 125.29, 118.93, 61.33, 52.04, 32.56, 28.91, 26.07, 16.06, 14.21.

**HRMS** (ESI^+^) calcd for C_17_H_21_NO_5_ [M+H] ^+^ 320.1492, found 320.1491.

**Methyl 4-(1-(2-(diethoxyphosphoryl) ethyl) cyclopropane-1-carboxamido) benzo- ate (6f)**

The title compound was prepared according to General Procedure A with 4CzTPN (4.0 mg, 5.0 µmol, 0.01 equiv.), NaOAc (82.03 mg, 1.0 mmol, 2.0 equiv.), Methyl 4-(4-chloro-2-methylenebutanamido) benzoat (133.86 mg, 0.5 mmol, 1.0 equiv.), NHC-2 (276.42 mg, 0.65 mmol, 1.3 equiv.), pyridine (63.28 mg, 0.8 mmol, 1.6 equiv.), diethyl (hydroxymethyl) phosphonate (109.28 mg, 0.65 mmol, 1.3 equiv.).

Purification by flash column chromatography (50% EtOAc/pentane) gave the title compound (95.85 mg, 0.25 mmol, 50%) as a yellow oil.

**TLC:** R_f_ = 0.50 (80% EtOAc/pentane, KMnO_4_ stain).

**^1^H NMR** (400 MHz, CDCl_3_) *δ* 8.82 (s, 1H), 8.01 – 7.96 (m, 2H), 7.78 – 7.72 (m, 2H), 4.17 – 4.06 (m, 4H), 3.89 (s, 3H), 2.04 – 1.93 (m, 4H), 1.34 – 1.31 (m, 6H), 1.28 (s, 2H), 0.77 – 0.72 (m, 2H).

**^13^C NMR** (101 MHz, CDCl_3_) *δ* 171.96, 166.76, 142.85, 130.67, 125.31, 119.19, 62.13, 62.07, 51.94, 30.23, 24.15, 16.48, 16.42, 15.63.

**HRMS** (ESI^+^) calcd for C_18_H_26_NO_6_P [M+H] ^+^ 384.1570, found 384.1566.

**1-(Cyclohexylmethyl)-*N*-phenylcyclopropane-1-carboxamide (6g)**

The title compound was prepared according to General Procedure A with 4CzTPN (4.0 mg, 5.0 µmol, 0.01 equiv.), NaOAc (82.03 mg, 1.0 mmol, 2.0 equiv.), 4-chloro-2-methylene-N-phenylbutanamide (104.83 mg, 0.5 mmol, 1.0 equiv.), NHC-1 (256.91 mg, 0.65 mmol, 1.3 equiv.), pyridine (63.28 mg, 0.8 mmol, 1.6 equiv.), Cyclohexanol (65.10 mg, 0.65 mmol, 1.3 equiv.).

Purification by flash column chromatography (2.5% EtOAc/pentane) gave the title compound (120 mg, 0.467 mmol, 93.3%) as a colorless oil.

**TLC:** R_f_ = 0.50 (10% EtOAc/pentane, KMnO_4_ stain).

**^1^H NMR** (400 MHz, CDCl_3_) *δ* 7.53 – 7.48 (m, 2H), 7.43 (s, 1H), 7.34 – 7.28 (m, 2H), 7.09 (t, *J* = 7.4 Hz, 1H), 1.85 (d, *J* = 11.4 Hz, 2H), 1.74 – 1.59 (m, 4H), 1.59 – 1.53 (m, 3H), 1.23 – 1.21 (m, 2H), 1.20 – 1.05 (m, 2H), 0.94 (qd, *J* = 10.8, 9.6, 5.2 Hz, 2H), 0.72 – 0.57 (m, 2H).

**^13^C NMR** (101 MHz, CDCl_3_) *δ* 172.43, 138.12, 128.98, 124.08, 119.89, 43.03, 37.25, 33.88, 26.39, 26.26, 24.24, 14.50.

**HRMS** (ESI^+^) calcd for C_17_H_23_NO [M+H] ^+^ 258.1852, found 258.1855.

***tert*-Butyl 3-((1-((4-(methoxycarbonyl) phenyl) carbamoyl) cyclopropyl) methyl) piperidine-1-carboxylate (6h)**

The title compound was prepared according to General Procedure B with 4CzTPN (4.0 mg, 5.0 µmol, 0.01 equiv.), NaOAc (82.03 mg, 1.0 mmol, 2.0 equiv.), Methyl 4-(4-chloro-2-methylenebutanamido) benzoate (133.86 mg, 0.5 mmol, 1.0 equiv.), NHC-1 (256.91 mg, 0.65 mmol, 1.3 equiv.), pyridine (63.28 mg, 0.8 mmol, 1.6 equiv.), tert-butyl 3-hydroxypiperidine-1-carboxylate (130.83 mg, 0.65 mmol, 1.3 equiv.).

Purification by flash column chromatography (10% EtOAc/pentane) gave the title compound (177.02 mg, 0.425 mmol, 85%) as a colorless oil.

**TLC:** R_f_ = 0.50 (30% EtOAc/pentane, KMnO_4_ stain).

**^1^H NMR** (400 MHz, CDCl_3_) *δ* 8.00 – 7.94 (m, 2H), 7.65 (d, *J* = 8.4 Hz, 2H), 4.06 (d, *J* = 9.9 Hz, 1H), 3.89 (s, 4H), 2.80 (t, *J* = 11.8 Hz, 1H), 2.64 – 2.31 (m, 2H), 1.96 – 1.85 (m, 1H), 1.76 (ddp, *J* = 10.0, 6.7, 3.2 Hz, 1H), 1.59 (dd, *J* = 13.8, 8.3 Hz, 2H), 1.29 – 1.05 (m, 4H), 0.76 – 0.63 (m, 2H).

**^13^C NMR** (101 MHz, CDCl_3_) *δ* 172.38, 166.68, 155.02, 142.44, 130.68, 125.38, 119.23, 79.59, 51.97, 38.92, 35.45, 31.44, 28.45, 24.80, 14.11.

**HRMS** (ESI^+^) calcd for C_23_H_32_N_2_O_5_ [M+H] ^+^ 417.2384, found 417.2385.

**1-((4-Oxocyclohexyl) methyl)-*N*-phenylcyclopropane-1-carboxamide (6i)**

The title compound was prepared according to General Procedure B with 4CzTPN (4.0 mg, 5.0 µmol, 0.01 equiv.), NaOAc (82.03 mg, 1.0 mmol, 2.0 equiv.), 4-chloro-2-methylene-N-phenylbutanamide (104.83 mg, 0.5 mmol, 1.0 equiv.), NHC-1 (256.91 mg, 0.65 mmol, 1.3 equiv.), pyridine (63.28 mg, 0.8 mmol, 1.6 equiv.), 4-hydroxycyclohexan-1-one (74.19 mg, 0.65 mmol, 1.3 equiv.).

Purification by flash column chromatography (15% EtOAc/pentane) gave the title compound (122.11 mg, 0.45 mmol, 90%) as a white sticky oil.

**TLC:** R_f_ = 0.50 (30% EtOAc/pentane, KMnO_4_ stain).

**^1^H NMR** (500 MHz, CDCl_3_) *δ* 7.67 (s, 1H), 7.53 – 7.48 (m, 2H), 7.34 – 7.27 (m, 2H), 7.12 – 7.07 (m, 1H), 2.36 – 2.28 (m, 4H), 2.18 (dp, *J* = 13.3, 3.1 Hz, 2H), 2.08 (dtd, *J* = 14.8, 7.3, 3.6 Hz, 1H), 1.68 (d, *J* = 7.2 Hz, 2H), 1.39 (qd, *J* = 11.9, 5.8 Hz, 2H), 1.25 – 1.18 (m, 2H), 0.72 – 0.66 (m, 2H).

**^13^C NMR** (126 MHz, CDCl_3_) *δ* 212.07, 172.08, 137.95, 128.98, 124.33, 120.16, 41.37, 40.79, 35.50, 33.02, 24.75, 13.93.

**HRMS** (ESI^+^) calcd for C_17_H_21_NO_2_ [M+H] ^+^ 272.1645, found 272.1647.

**4-((1-(Phenylcarbamoyl) cyclopropyl) methyl) cyclohexane-1-carboxylic acid (6j)**

The title compound was prepared according to General Procedure B with 4CzTPN (4.0 mg, 5.0 µmol, 0.01 equiv.), NaOAc (82.03 mg, 1.0 mmol, 2.0 equiv.), 4-chloro-2-methylene-N-phenylbutanamide (104.83 mg, 0.5 mmol, 1.0 equiv.), NHC-1 (256.91 mg, 0.65 mmol, 1.3 equiv.), pyridine (63.28 mg, 0.8 mmol, 1.6 equiv.), 4-hydroxycyclohexane-1-carboxylic acid (93.71 mg, 0.65 mmol, 1.3 equiv.).

Purification by flash column chromatography (20% EtOAc/pentane + 0.5% acetic acid) gave the title compound (130.2 mg, 0.435 mmol, 87%) as a yellow oil.

**TLC:** R_f_ = 0.50 (40% EtOAc/pentane + 0.5% acetic acid, KMnO_4_ stain).

**^1^H NMR** (400 MHz, Chloroform-d) *δ* 10.34 (s, 1H), 7.56 (s, 1H), 7.50 – 7.44 (m, 2H), 7.29 (t, *J* = 7.7 Hz, 2H), 7.08 (td, *J* = 7.3, 1.4 Hz, 1H), 1.98 (ddd, *J* = 21.7, 15.9, 7.0 Hz, 3H), 1.77 – 1.59 (m, 2H), 1.59 – 1.48 (m, 3H), 1.41 (qd, *J* = 14.3, 13.7, 3.8 Hz, 1H), 1.31 – 1.24 (m, 1H), 1.23 – 1.16 (m, 2H), 1.06 – 0.89 (m, 1H), 0.65 (td, *J* = 4.9, 2.4 Hz, 2H).

**^13^C NMR** (101 MHz, CDCl_3_) *δ* 182.07, 181.61, 177.32, 172.69, 172.66, 137.93, 137.88, 129.00, 124.42, 124.36, 120.33, 120.29, 43.07, 42.63, 41.34, 39.87, 36.44, 35.42, 32.51, 29.77, 28.63, 26.17, 24.53, 24.35, 20.91, 14.30, 14.14.

**HRMS** (ESI^+^) calcd for C_18_H_23_NO_3_ [M+H] ^+^ 302.1751, found 302.1754.

***N*-Phenyl-1-((tetrahydro-2*H*-pyran-4-yl) methyl) cyclopropane-1-carboxamide (6k)**

The title compound was prepared according to General Procedure B with 4CzTPN (4.0 mg, 5.0 µmol, 0.01 equiv.), NaOAc (82.03 mg, 1.0 mmol, 2.0 equiv.), 4-chloro-2-methylene-N-phenylbutanamide (104.83 mg, 0.5 mmol, 1.0 equiv.), NHC-1 (256.91 mg, 0.65 mmol, 1.3 equiv.), pyridine (63.28 mg, 0.8 mmol, 1.6 equiv.), tetrahydro-2H-pyran-4-ol (66.38 mg, 0.65 mmol, 1.3 equiv.).

Purification by flash column chromatography (10% EtOAc/pentane) gave the title compound (112.82 mg, 0.435 mmol, 87%) as a brown oil.

**TLC:** R_f_ = 0.50 (30% EtOAc/pentane, KMnO_4_ stain).

**^1^H NMR** (400 MHz, CDCl_3_) *δ* 7.52 – 7.47 (m, 2H), 7.44 (s, 1H), 7.34 – 7.28 (m, 2H), 7.13 – 7.07 (m, 1H), 3.93 (ddd, *J* = 11.6, 4.5, 1.7 Hz, 2H), 3.35 (td, *J* = 11.8, 2.0 Hz, 2H), 1.85 (dtt, *J* = 14.8, 7.4, 3.5 Hz, 1H), 1.72 (ddd, *J* = 13.1, 4.0, 2.0 Hz, 2H), 1.61 (d, *J* = 7.1 Hz, 2H), 1.31 (dtd, *J* = 13.4, 11.9, 4.5 Hz, 2H), 1.23 – 1.18 (m, 2H), 0.73 – 0.64 (m, 2H).

**^13^C NMR** (126 MHz, CDCl_3_) *δ* 172.04, 137.94, 129.03, 124.27, 119.94, 67.91, 42.70, 34.55, 33.48, 24.11, 14.02.

**HRMS** (ESI^+^) calcd for C_16_H_21_NO_2_ [M+H] ^+^ 260.1645, found 260.1648.

***N*-Phenyl-1-((2-phenyl-1,3-dioxan-5-yl) methyl) cyclopropane-1-carboxamide (6l)**

The title compound was prepared according to General Procedure B with 4CzTPN (4.0 mg, 5.0 µmol, 0.01 equiv.), NaOAc (82.03 mg, 1.0 mmol, 2.0 equiv.), 4-chloro-2-methylene-N-phenylbutanamide (104.83 mg, 0.5 mmol, 1.0 equiv.), NHC-1 (256.91 mg, 0.65 mmol, 1.3 equiv.), pyridine (63.28 mg, 0.8 mmol, 1.6 equiv.), 2-phenyl-1,3-dioxan-5-ol (117.13 mg, 0.65 mmol, 1.3 equiv.).

Purification by flash column chromatography (5% EtOAc/pentane) gave the title compound (118.1 mg, 0.35 mmol, 70%) as a light brown oil.

**TLC:** R_f_ = 0.50 (20% EtOAc/pentane, KMnO_4_ stain).

**^1^H NMR** (400 MHz, CDCl_3_) *δ* 8.40 (s, 1H), 7.52 (dq, *J* = 5.4, 3.0 Hz, 2H), 7.38 (dd, *J* = 5.1, 2.0 Hz, 3H), 7.35 – 7.31 (m, 2H), 7.14 (dd, *J* = 8.6, 7.2 Hz, 2H), 7.01 – 6.95 (m, 1H), 5.60 (s, 1H), 4.15 (d, *J* = 2.0 Hz, 4H), 2.23 (d, *J* = 8.3 Hz, 2H), 1.67 (td, *J* = 8.2, 4.1 Hz, 1H), 1.39 (q, *J* = 3.9 Hz, 2H), 0.76 (q, *J* = 3.9 Hz, 2H).

**^13^C NMR** (126 MHz, CDCl_3_) *δ* 172.72, 138.47, 138.19, 129.18, 128.69, 128.40, 126.07, 123.78, 119.88, 102.13, 70.36, 33.50, 33.24, 23.66, 16.54.

**HRMS** (ESI^+^) calcd for C_21_H_23_NO_3_ [M+H] ^+^ 338.1751, found 338.1753.

**Benzyl 3-((1-(phenylcarbamoyl) cyclopropyl) methyl) azetidine-1-carboxylate (6m)**

The title compound was prepared according to General Procedure B with 4CzTPN (4.0 mg, 5.0 µmol, 0.01 equiv.), NaOAc (82.03 mg, 1.0 mmol, 2.0 equiv.), 4-chloro-2-methylene-N-phenylbutanamide (104.83 mg, 0.5 mmol, 1.0 equiv.), NHC-1 (256.91 mg, 0.65 mmol, 1.3 equiv.), pyridine (63.28 mg, 0.8 mmol, 1.6 equiv.), benzyl 3-hydroxyazetidine-1-carboxylate (134.70 mg, 0.65 mmol, 1.3 equiv.).

Purification by flash column chromatography (15% EtOAc/pentane) gave the title compound (109.34 mg, 0.30 mmol, 60%) as a brown oil.

**TLC:** R_f_ = 0.50 (30% EtOAc/pentane, KMnO_4_ stain).

**^1^H NMR** (400 MHz, CDCl_3_) *δ* 7.54 (s, 1H), 7.48 – 7.43 (m, 2H), 7.36 – 7.25 (m, 7H), 7.11 – 7.05 (m, 1H), 5.06 (s, 2H), 4.09 (t, *J* = 8.5 Hz, 2H), 3.68 (dd, *J* = 8.6, 5.8 Hz, 2H), 2.79 (tt, *J* = 7.8, 5.8 Hz, 1H), 1.95 (s, 2H), 1.17 – 1.07 (m, 2H), 0.71 – 0.64 (m, 2H).

**^13^C NMR** (126 MHz, CDCl_3_) *δ* 171.67, 156.37, 137.82, 136.64, 128.96, 128.51, 128.08, 127.92, 124.43, 120.33, 66.61, 39.16, 28.09, 25.09, 12.79.

**HRMS** (ESI^+^) calcd for C_22_H_24_N_2_O_3_ [M+H] ^+^ 365.1860, found 365.1862.

**Benzyl 3-((1-(phenylcarbamoyl) cyclopropyl) methyl) pyrrolidine-1-carboxylate (6n)**

The title compound was prepared according to General Procedure B with 4CzTPN (4.0 mg, 5.0 µmol, 0.01 equiv.), NaOAc (82.03 mg, 1.0 mmol, 2.0 equiv.), 4-chloro-2-methylene-N-phenylbutanamide (104.83 mg, 0.5 mmol, 1.0 equiv.), NHC-1 (256.91 mg, 0.65 mmol, 1.3 equiv.), pyridine (63.28 mg, 0.8 mmol, 1.6 equiv.), benzyl 3-hydroxypyrrolidine-1-carboxylate (143.82 mg, 0.65 mmol, 1.3 equiv.).

Purification by flash column chromatography (20% EtOAc/pentane) gave the title compound (113.54 mg, 0.30 mmol, 60%) as a yellow oil.

**TLC:** R_f_ = 0.50 (40% EtOAc/pentane, KMnO_4_ stain).

**^1^H NMR** (400 MHz, CDCl_3_) *δ* 7.58 (d, *J* = 6.7 Hz, 1H), 7.48 (dd, *J* = 8.0, 4.8 Hz, 2H), 7.36 – 7.25 (m, 7H), 7.08 (t, *J* = 7.4 Hz, 1H), 5.13 – 5.06 (m, 2H), 3.67 (ddd, *J* = 20.3, 10.6, 7.3 Hz, 1H), 3.51 (tdd, *J* = 11.1, 8.2, 2.6 Hz, 1H), 3.28 (tt, *J* = 10.3, 6.5 Hz, 1H), 3.04 – 2.90 (m, 2H), 2.41 (q, *J* = 9.9, 8.7 Hz, 1H), 1.73 (dd, *J* = 7.2, 4.3 Hz, 1H), 1.66 – 1.44 (m, 2H), 1.21 – 1.14 (m, 2H), 0.72 – 0.62 (m, 2H).

**^13^C NMR** (101 MHz, CDCl_3_) *δ* 171.76, 154.87, 137.90, 136.99, 129.01, 128.50, 127.96, 124.40, 120.19, 66.73, 51.86, 51.34, 45.91, 45.60, 38.36, 37.51, 32.09, 25.40.

**HRMS** (ESI^+^) calcd for C_23_H_26_N_2_O_3_ [M+H] ^+^ 379.2016, found 379.2015.

***tert*-butyl 3-((1-(phenylcarbamoyl) cyclopropyl) methyl)-1-oxa-8-azaspiro [4.5] decane-8-carboxylate (6o)**

The title compound was prepared according to General Procedure B with 4CzTPN (4.0 mg, 5.0 µmol, 0.01 equiv.), NaOAc (82.03 mg, 1.0 mmol, 2.0 equiv.), 4-chloro-2-methylene-N-phenylbutanamide (104.83 mg, 0.5 mmol, 1.0 equiv.), NHC-1 (256.91 mg, 0.65 mmol, 1.3 equiv.), pyridine (63.28 mg, 0.8 mmol, 1.6 equiv.), tert-butyl 3-hydroxy-1-oxa-8-azaspiro [4.5] decane-8-carboxylate (120.2 mg, 0.65 mmol, 1.3 equiv.).

Purification by flash column chromatography (20% EtOAc/pentane) gave the title compound (124.32 mg, 0.30 mmol, 60%) as a yellow oil.

**TLC:** R_f_ = 0.50 (40% EtOAc/pentane, KMnO_4_ stain).

**^1^H NMR** (400 MHz, CDCl_3_) *δ* 7.56 – 7.46 (m, 3H), 7.31 (dd, *J* = 8.5, 7.3 Hz, 2H), 7.10 (t, *J* = 7.4 Hz, 1H), 4.01 (dd, *J* = 8.6, 6.8 Hz, 1H), 3.55 (s, 2H), 3.48 (t, *J* = 8.6 Hz, 1H), 3.35 – 3.23 (m, 2H), 2.53 (dq, *J* = 15.4, 7.7 Hz, 1H), 2.02 (dd, *J* = 12.3, 7.6 Hz, 1H), 1.83 – 1.69 (m, 2H), 1.58 (dd, *J* = 7.6, 4.9 Hz, 3H), 1.45 (s, 10H), 1.35 (dd, *J* = 12.4, 9.3 Hz, 1H), 1.25 (t, *J* = 12.7 Hz, 1H), 1.19 (q, *J* = 2.9 Hz, 2H), 0.77 – 0.62 (m, 2H).

**^13^C NMR** (101 MHz, CDCl_3_) *δ* 171.86, 154.87, 137.88, 129.04, 124.39, 120.11, 80.20, 79.45, 71.82, 44.06, 38.45, 38.33, 28.50, 25.62, 13.98, 13.83.

**HRMS** (ESI^+^) calcd for C_24_H_34_N_2_O_4_ [M+H] ^+^ 415.2591, found 415.2593.

***tert*-Butyl 6-((1-(phenylcarbamoyl) cyclopropyl) methyl)-2-azaspiro [3.4] octane-2-carboxylate (6p)**

The title compound was prepared according to General Procedure B with 4CzTPN (4.0 mg, 5.0 µmol, 0.01 equiv.), NaOAc (82.03 mg, 1.0 mmol, 2.0 equiv.), 4-chloro-2-methylene-N-phenylbutanamide (104.83 mg, 0.5 mmol, 1.0 equiv.), NHC-1 (256.91 mg, 0.65 mmol, 1.3 equiv.), pyridine (63.28 mg, 0.8 mmol, 1.6 equiv.), tert-butyl 6-hydroxy-2-azaspiro [3,4] octane-2-carboxylate (147.75 mg, 0.65 mmol, 1.3 equiv.).

Purification by flash column chromatography (10% EtOAc/pentane) gave the title compound (142.30 mg, 0.34 mmol, 68%) as a red oil.

**TLC:** R_f_ = 0.50 (30% EtOAc/pentane, KMnO_4_ stain).

**^1^H NMR** (400 MHz, CDCl_3_) *δ* 7.64 (s, 1H), 7.50 (d, *J* = 8.0 Hz, 2H), 7.29 (t, *J* = 7.7 Hz, 2H), 7.11 – 7.05 (m, 1H), 3.75 (s, 2H), 3.71 – 3.65 (m, 2H), 2.10 (td, *J* = 7.6, 4.9 Hz, 2H), 1.90 (ddd, *J* = 12.3, 8.0, 4.4 Hz, 1H), 1.84 (dd, *J* = 10.2, 5.8 Hz, 1H), 1.81 – 1.77 (m, 1H), 1.76 – 1.71 (m, 1H), 1.71 – 1.65 (m, 1H), 1.44 (s, 10H), 1.37 (dd, *J* = 11.4, 3.9 Hz, 1H), 1.28 – 1.22 (m, 1H), 1.20 – 1.13 (m, 2H), 0.66 (d, *J* = 2.8 Hz, 2H).

**^13^C NMR** (101 MHz, CDCl_3_) *δ* 172.21, 156.41, 138.11, 128.95, 124.19, 120.10, 79.27, 45.47, 41.23, 40.61, 38.35, 37.68, 31.95, 28.46, 25.80, 13.76, 13.67.

**HRMS** (ESI^+^) calcd for C_23_H_32_N_2_O_3_ [M+H] ^+^ 385.2485, found 385.2484.

**1-Isobutyl-*N*-phenylcyclopropane-1-carboxamide (6q)**

The title compound was prepared according to General Procedure B with 4CzTPN (4.0 mg, 5.0 µmol, 0.01 equiv.), NaOAc (82.03 mg, 1.0 mmol, 2.0 equiv.), 4-chloro-2-methylene-N-phenylbutanamide (104.83 mg, 0.5 mmol, 1.0 equiv.), NHC-1 (256.91 mg, 0.65 mmol, 1.3 equiv.), pyridine (63.28 mg, 0.8 mmol, 1.6 equiv.), propan-2-ol (39.07 mg, 0.65 mmol, 1.3 equiv.).

Purification by flash column chromatography (1% EtOAc/pentane) gave the title compound (103.22 mg, 0.475 mmol, 95%) as a light brown oil.

**TLC:** R_f_ = 0.50 (5% EtOAc/pentane, KMnO_4_ stain).

^1^H NMR (400 MHz, CDCl_3_) *δ* 7.53 – 7.47 (m, 2H), 7.40 (s, 1H), 7.34 – 7.28 (m, 2H), 7.08 (td, *J* = 7.4, 1.2 Hz, 1H), 1.93 (dq, *J* = 13.5, 6.8 Hz, 1H), 1.56 (d, *J* = 7.3 Hz, 2H), 1.24 – 1.20 (m, 2H), 0.98 (d, *J* = 6.7 Hz, 6H), 0.70 – 0.64 (m, 2H).

**^13^C NMR** (126 MHz, CDCl_3_) *δ* 172.31, 138.06, 128.99, 124.12, 119.86, 44.26, 27.76, 24.74, 23.11, 14.39.

**HRMS** (ESI^+^) calcd for C_14_H_19_NO [M+H] ^+^ 218.1540, found 218.1541.

**1-(4-Hydroxy-2,4-dimethylpentyl)-*N*-phenylcyclopropane-1-carboxamide (6r)**

The title compound was prepared according to General Procedure B with 4CzTPN (4.0 mg, 5.0 µmol, 0.01 equiv.), NaOAc (82.03 mg, 1.0 mmol, 2.0 equiv.), 4-chloro-2-methylene-N-phenylbutanamide (104.83 mg, 0.5 mmol, 1.0 equiv.), NHC-1 (256.91 mg, 0.65 mmol, 1.3 equiv.), pyridine (63.28 mg, 0.8 mmol, 1.6 equiv.), 2-methylpentane-2,4-diol (76.81 mg, 0.65 mmol, 1.3 equiv.).

Purification by flash column chromatography (5% EtOAc/pentane) gave the title compound (102.9 mg, 0.375 mmol, 75%) as a colorless oil.

**TLC:** R_f_ = 0.50 (15% EtOAc/pentane, KMnO_4_ stain).

**^1^H NMR** (400 MHz, CDCl_3_) *δ* 8.76 (s, 1H), 7.64 – 7.58 (m, 2H), 7.30 – 7.24 (m, 2H), 7.04 (td, *J* = 7.3, 1.2 Hz, 1H), 2.72 (dt, *J* = 15.3, 2.1 Hz, 1H), 2.22 – 2.11 (m, 1H), 1.87 (s, 1H), 1.53 (ddd, *J* = 22.4, 11.9, 6.3 Hz, 2H), 1.31 (s, 3H), 1.30 – 1.26 (m, 1H), 1.25 (s, 3H), 1.04 (d, *J* = 6.7 Hz, 3H), 0.98 (ddd, *J* = 9.9, 6.6, 3.6 Hz, 1H), 0.81 – 0.74 (m, 2H), 0.52 (ddd, *J* = 9.6, 6.6, 3.5 Hz, 1H).

**^13^C NMR** (126 MHz, CDCl_3_) *δ* 173.08, 138.89, 128.73, 123.70, 120.26, 72.65, 50.42, 43.11, 32.58, 28.67, 28.50, 24.18, 23.06, 16.23, 14.64.

**HRMS** (ESI^+^) calcd. for C_17_H_25_NO_2_ [M+H] ^+^276.1958, found 276.1965.

**Methyl 4-(1-(2-methyl-4-phenylbutyl) cyclopropane-1-carboxamido) benzoate (6s)**

The title compound was prepared according to General Procedure B with 4CzTPN (4.0 mg, 5.0 µmol, 0.01 equiv.), NaOAc (82.03 mg, 1.0 mmol, 2.0 equiv.), Methyl 4-(4-chloro-2-methylenebutanamido) benzoate (133.86 mg, 0.5 mmol, 1.0 equiv.), NHC-1 (256.91 mg, 0.65 mmol, 1.3 equiv.), pyridine (63.28 mg, 0.8 mmol, 1.6 equiv.), 4-phenylbutan-2-ol (97.64 mg, 0.65 mmol, 1.3 equiv.).

Purification by flash column chromatography (10% EtOAc/pentane) gave the title compound (160.6 mg, 0.44 mmol, 88%) as a colorless oil.

**TLC:** R_f_ = 0.50 (30% EtOAc/pentane, KMnO_4_ stain).

**^1^H NMR** (400 MHz, CDCl_3_) *δ* 7.98 – 7.92 (m, 2H), 7.63 (s, 1H), 7.51 – 7.43 (m, 2H), 7.27 – 7.19 (m, 2H), 7.14 (dt, *J* = 5.9, 1.4 Hz, 3H), 3.87 (s, 3H), 2.76 – 2.49 (m, 2H), 1.89 (dd, *J* = 14.8, 5.5 Hz, 1H), 1.84 – 1.67 (m, 2H), 1.51 (dddd, *J* = 13.6, 9.5, 7.6, 5.8 Hz, 1H), 1.42 – 1.23 (m, 2H), 1.16 (ddd, *J* = 10.2, 6.0, 3.8 Hz, 1H), 1.03 (d, *J* = 6.5 Hz, 3H), 0.75 – 0.59 (m, 2H).

**^13^C NMR** (101 MHz, CDCl_3_) *δ* 172.60, 166.74, 142.29, 142.24, 130.77, 128.47, 128.34, 125.86, 125.42, 119.08, 52.03, 42.28, 38.94, 33.15, 31.81, 24.73, 19.90, 15.04, 14.56.

**HRMS** (ESI^+^) calcd for C_23_H_27_NO_3_ [M+H] ^+^ 366.2063, found 366.2062.

**Methyl 4-(1-(((1S,4R)-bicyclo [2,2,1] heptan-2-yl) methyl) cyclopropane-1-carboxamido) benzoate (6t)**

The title compound was prepared according to General Procedure B with 4CzTPN (4.0 mg, 5.0 µmol, 0.01 equiv.), NaOAc (82.03 mg, 1.0 mmol, 2.0 equiv.), Methyl 4-(4-chloro-2-methylenebutanamido) benzoate (133.86 mg, 0.5 mmol, 1.0 equiv.), NHC-1 (256.91 mg, 0.65 mmol, 1.3 equiv.), pyridine (63.28 mg, 0.8 mmol, 1.6 equiv.), (1S,4R)-bicyclo [2,2,1] heptan-2-ol (72.91 mg, 0.65 mmol, 1.3 equiv.).

Purification by flash column chromatography (5% EtOAc/pentane) gave the title compound (135.06 mg, 0.4125 mmol, 83%) as a colorless oil.

**TLC:** R_f_ = 0.50 (10% EtOAc/pentane, KMnO_4_ stain).

**^1^H NMR** (400 MHz, CDCl_3_) *δ* 7.98 (d, *J* = 8.7 Hz, 2H), 7.77 (s, 1H), 7.64 – 7.58 (m, 2H), 3.89 (s, 3H), 2.20 (d, *J* = 4.2 Hz, 1H), 2.12 (d, *J* = 3.7 Hz, 1H), 1.67 – 1.57 (m, 3H), 1.54 – 1.38 (m, 3H), 1.30 – 1.22 (m, 2H), 1.18 – 1.04 (m, 5H), 0.75 – 0.66 (m, 2H).

**^13^C NMR** (101 MHz, CDCl_3_) *δ* 172.88, 166.69, 142.40, 130.81, 125.39, 118.96, 52.00, 41.98, 41.09, 40.99, 38.82, 36.68, 35.35, 35.29, 29.99, 28.70, 25.72, 14.45, 13.94.

**HRMS** (ESI^+^) calcd for C_20_H_25_NO_3_ [M+H] ^+^ 328.1907, found 328.1911.

**Methyl 4-(1-(((1r,3r,5r,7r)-adamantan-2-yl) methyl) cyclopropane-1-carboxamido) benzoate (6u)**

The title compound was prepared according to General Procedure B with 4CzTPN (4.0 mg, 5.0 µmol, 0.01 equiv.), NaOAc (82.03 mg, 1.0 mmol, 2.0 equiv.), Methyl 4-(4-chloro-2-methylenebutanamido) benzoate (133.86 mg, 0.5 mmol, 1.0 equiv.), NHC-1 (256.91 mg, 0.65 mmol, 1.3 equiv.), pyridine (63.28 mg, 0.8 mmol, 1.6 equiv.), (1r,3r,5r,7r)-adamantan-2-ol (98.96 mg, 0.65 mmol, 1.3 equiv.).

Purification by flash column chromatography (1% EtOAc/pentane) gave the title compound (114.84 mg, 0.3125 mmol, 63%) as a yellow oil.

**TLC:** R_f_ = 0.50 (5% EtOAc/pentane, KMnO_4_ stain).

**^1^H NMR** (400 MHz, CDCl_3_) *δ* 8.02 – 7.97 (m, 2H), 7.64 (d, *J* = 3.5 Hz, 1H), 7.61 – 7.55 (m, 2H), 3.89 (s, 3H), 2.01 – 1.94 (m, 1H), 1.82 (d, *J* = 19.7 Hz, 10H), 1.77 – 1.69 (m, 4H), 1.62 – 1.51 (m, 2H), 1.22 (q, *J* = 4.2 Hz, 2H), 0.75 – 0.70 (m, 2H).

**^13^C NMR** (101 MHz, CDCl_3_) *δ* 172.76, 166.65, 142.31, 130.86, 125.42, 118.84, 52.01, 43.74, 39.12, 38.30, 38.17, 32.64, 31.78, 27.97, 27.94, 25.56, 14.26.

**HRMS** (ESI^+^) calcd for C_23_H_29_NO_3_ [M+H] ^+^ 368.2220, found 368.2218.

***tert*-Butyl 3-methyl-3-((1-(phenylcarbamoyl) cyclopropyl) methyl) azetidine-1-carboxylate (6v)**

The title compound was prepared according to General Procedure C with 4CzTPN (4.0 mg, 5.0 µmol, 0.01 equiv.), NaOAc (82.03 mg, 1.0 mmol, 2.0 equiv.), 4-chloro-2-methylene-N-phenylbutanamide (104.83 mg, 0.5 mmol, 1.0 equiv.), NHC-3 (301.1 mg, 0.65 mmol, 1.3 equiv.), pyridine (63.28 mg, 0.8 mmol, 1.6 equiv.), tert-butyl 3-hydroxy-3-methylazetidine-1-carboxylate (121.71 mg, 0.65 mmol, 1.3 equiv.).

Purification by flash column chromatography (10% EtOAc/pentane) gave the title compound (167.06 mg, 0.485 mmol, 97%) as a brown oil.

**TLC:** R_f_ = 0.50 (30% EtOAc/pentane, KMnO_4_ stain).

**^1^H NMR** (400 MHz, CDCl_3_) *δ* 7.62 (s, 1H), 7.48 – 7.44 (m, 2H), 7.34 – 7.26 (m, 2H), 7.12 – 7.06 (m, 1H), 3.72 (d, *J* = 8.3 Hz, 2H), 3.54 (d, *J* = 8.3 Hz, 2H), 1.95 (s, 2H), 1.39 (d, *J* = 5.1 Hz, 12H), 1.15 – 1.09 (m, 2H), 0.73 – 0.67 (m, 2H).

**^13^C NMR** (101 MHz, CDCl_3_) *δ* 171.74, 156.68, 137.80, 129.00, 124.41, 120.23, 79.42, 45.01, 34.70, 28.42, 24.82, 24.78, 12.86.

**HRMS** (ESI^+^) calcd for C_20_H_28_N_2_O_3_ [M+H] ^+^ 345.2172, found 345.2168.

***tert-*Butyl 4-methyl-4-((1-(phenylcarbamoyl) cyclopropyl) methyl) piperidine-1-carboxylate (6w)**

The title compound was prepared according to General Procedure C with 4CzTPN (4.0 mg, 5.0 µmol, 0.01 equiv.), NaOAc (82.03 mg, 1.0 mmol, 2.0 equiv.), 4-chloro-2-methylene-N-phenylbutanamide (104.83 mg, 0.5 mmol, 1.0 equiv.), NHC-3 (301.1 mg, 0.65 mmol, 1.3 equiv.), pyridine (63.28 mg, 0.8 mmol, 1.6 equiv.), tert-butyl 4-hydroxy-4-methylpiperidine-1-carboxylate (139.95 mg, 0.65 mmol, 1.3 equiv.).

Purification by flash column chromatography (10% EtOAc/pentane) gave the title compound (149.01 mg, 0.40 mmol, 80%) as a yellow oil.

**TLC:** R_f_ = 0.50 (30% EtOAc/pentane, KMnO_4_ stain).

**^1^H NMR** (400 MHz, CDCl_3_) *δ* 7.61 (s, 1H), 7.50 – 7.45 (m, 2H), 7.30 (s, 2H), 7.12 – 7.05 (m, 1H), 3.66 (d, *J* = 13.4 Hz, 2H), 3.04 (ddd, *J* = 13.7, 9.9, 3.7 Hz, 2H), 1.68 (s, 2H), 1.43 (s, 9H), 1.38 (td, *J* = 9.0, 4.5 Hz, 3H), 1.30 – 1.23 (m, 1H), 1.12 (t, *J* = 3.2 Hz, 2H), 1.07 (s, 3H), 0.73 – 0.65 (m, 2H).

**^13^C NMR** (101 MHz, CDCl_3_) *δ* 172.40, 154.98, 137.98, 129.01, 124.21, 119.94, 79.37, 49.02, 37.41, 33.90, 28.50, 24.06, 22.63, 13.18.

**HRMS** (ESI^+^) calcd for C_22_H_32_N_2_O_3_ [M+H] ^+^ 373.2485, found 373.2487

***tert-*Butyl (2,2-dimethyl-3-(1-(phenylcarbamoyl) cyclopropyl) propyl) carbamate (6x)**

The title compound was prepared according to General Procedure C with 4CzTPN (4.0 mg, 5.0 µmol, 0.01 equiv.), NaOAc (82.03 mg, 1.0 mmol, 2.0 equiv.), 4-chloro-2-methylene-N-phenylbutanamide (104.83 mg, 0.5 mmol, 1.0 equiv.), NHC-3 (301.1 mg, 0.65 mmol, 1.3 equiv.), pyridine (63.28 mg, 0.8 mmol, 1.6 equiv.), tert-butyl (2-hydroxy-2-methylpropyl) carbamate (123.02 mg, 0.65 mmol, 1.3 equiv.).

Purification by flash column chromatography (10% EtOAc/pentane) gave the title compound (170.26 mg, 0.49 mmol, 98%) as a brown oil.

**TLC:** R_f_ = 0.50 (30% EtOAc/pentane, KMnO_4_ stain).

**^1^H NMR** (400 MHz, CDCl_3_) *δ* 7.79 (s, 1H), 7.53 – 7.48 (m, 2H), 7.32 – 7.26 (m, 2H), 7.12 – 7.05 (m, 1H), 5.01 (t, *J* = 6.6 Hz, 1H), 2.96 (d, *J* = 6.7 Hz, 2H), 1.65 (s, 2H), 1.17 – 1.10 (m, 2H), 0.95 (s, 6H), 0.75 – 0.67 (m, 2H).

**^13^C NMR** (101 MHz, CDCl_3_) *δ* 172.67, 156.65, 137.99, 128.96, 124.25, 120.22, 79.17, 50.78, 44.35, 36.76, 28.47, 25.98, 24.17, 13.77.

**HRMS** (ESI^+^) calcd for C_20_H_30_N_2_O_3_ [M+H] ^+^ 347.2329, found 347.2331.

**Methyl 4-(1-neopentylcyclopropane-1-carboxamido) benzoate (6y)**

The title compound was prepared according to General Procedure C with 4CzTPN (4.0 mg, 5.0 µmol, 0.01 equiv.), NaOAc (82.03 mg, 1.0 mmol, 2.0 equiv.), Methyl 4-(4-chloro-2-methylenebutanamido) benzoate (133.86 mg, 0.5 mmol, 1.0 equiv.), NHC-3 (301.1 mg, 0.65 mmol, 1.3 equiv.), pyridine (63.28 mg, 0.8 mmol, 1.6 equiv.), 2-methylpropan-2-ol (48.18 mg, 0.65 mmol, 1.3 equiv.).

Purification by flash column chromatography (5% EtOAc/pentane) gave the title compound (130.22 mg, 0.45 mmol, 90%) as a yellow oil.

**TLC:** R_f_ = 0.50 (15% EtOAc/pentane, KMnO_4_ stain).

**^1^H NMR** (400 MHz, CDCl_3_) *δ* 8.00 – 7.95 (m, 2H), 7.91 (s, 1H), 7.63 – 7.58 (m, 2H), 3.89 (s, 3H), 1.66 (s, 2H), 1.20 – 1.12 (m, 2H), 0.97 (s, 9H), 0.72 – 0.66 (m, 2H).

**^13^C NMR** (101 MHz, CDCl_3_) *δ* 172.97, 166.75, 142.46, 130.81, 125.27, 118.87, 52.00, 49.44, 32.93, 30.30, 24.79, 13.42.

**HRMS** (ESI^+^) calcd for C_17_H_23_NO_3_ [M+H] ^+^ 290.1750, found 290.1748.

**Methyl 4-(1-(2,2-dimethyl-4-phenylbutyl) cyclopropane-1-carboxamido) benzoate (6z)**

The title compound was prepared according to General Procedure C with 4CzTPN (4.0 mg, 5.0 µmol, 0.01 equiv.), NaOAc (82.03 mg, 1.0 mmol, 2.0 equiv.), Methyl 4-(4-chloro-2-methylenebutanamido) benzoate (133.86 mg, 0.5 mmol, 1.0 equiv.), NHC-3 (301.1 mg, 0.65 mmol, 1.3 equiv.), pyridine (63.28 mg, 0.8 mmol, 1.6 equiv.), 2-methyl-4-phenylbutan-2-ol (106.77 mg, 0.65 mmol, 1.3 equiv.).

Purification by flash column chromatography (7.5% EtOAc/pentane) gave the title compound (142.31 mg, 0.375 mmol, 75%) as a brown oil.

**TLC:** R_f_ = 0.50 (15% EtOAc/pentane, KMnO_4_ stain).

**^1^H NMR** (400 MHz, CDCl_3_) *δ* 7.99 – 7.94 (m, 2H), 7.73 (s, 1H), 7.59 – 7.53 (m, 2H), 7.24 – 7.18 (m, 2H), 7.16 – 7.07 (m, 3H), 3.88 (s, 3H), 2.59 – 2.51 (m, 2H), 1.73 (s, 2H), 1.61 – 1.53 (m, 2H), 1.20 – 1.13 (m, 2H), 1.03 (s, 6H), 0.76 – 0.67 (m, 2H).

**^13^C NMR** (101 MHz, CDCl_3_) *δ* 172.83, 166.72, 142.90, 142.32, 130.87, 128.38, 128.27, 125.69, 125.43, 118.88, 52.03, 47.98, 45.59, 35.60, 30.65, 27.42, 24.70, 13.49.

**HRMS** (ESI^+^) calcd for C_24_H_29_NO_3_ [M+H] ^+^ 380.2215, found 380.2206.

**Methyl 4-(1-(3-methoxy-2,2-dimethylpropyl) cyclopropane-1-carboxamido) benzoate (6aa)**

The title compound was prepared according to General Procedure C with 4CzTPN (4.0 mg, 5.0 µmol, 0.01 equiv.), NaOAc (82.03 mg, 1.0 mmol, 2.0 equiv.), Methyl 4-(4-chloro-2-methylenebutanamido) benzoate (133.86 mg, 0.5 mmol, 1.0 equiv.), NHC-3 (301.1 mg, 0.65 mmol, 1.3 equiv.), pyridine (63.28 mg, 0.8 mmol, 1.6 equiv.), 1-methoxy-2-methylpropan-2-ol (67.70 mg, 0.65 mmol, 1.3 equiv.).

Purification by flash column chromatography (5% EtOAc/pentane) gave the title compound (150.23 mg, 0.47 mmol, 94%) as a colorless oil.

**TLC:** R_f_ = 0.50 (15% EtOAc/pentane, KMnO_4_ stain).

**^1^H NMR** (400 MHz, CDCl_3_) *δ* 8.64 (s, 1H), 8.06 – 7.95 (m, 2H), 7.70 – 7.58 (m, 2H), 3.89 (s, 3H), 3.29 (s, 3H), 3.12 (s, 2H), 1.75 (s, 2H), 1.29 (q, *J* = 4.0 Hz, 2H), 0.98 (s, 6H), 0.72 (q, *J* = 4.0 Hz, 2H).

**^13^C NMR** (101 MHz, CDCl_3_) *δ* 173.19, 166.74, 142.93, 130.75, 125.08, 118.83, 80.97, 59.49, 51.94, 42.24, 36.24, 26.41, 23.60, 15.82.

**HRMS** (ESI^+^) calcd for C_18_H_25_NO_4_ [M+H] ^+^ 320.1856, found 320.1854.

**Methyl 4-(1-((3-methyloxetan-3-yl) methyl) cyclopropane-1-carboxamido) benzoate (6ab)**

The title compound was prepared according to General Procedure C with 4CzTPN (4.0 mg, 5.0 µmol, 0.01 equiv.), NaOAc (82.03 mg, 1.0 mmol, 2.0 equiv.), Methyl 4-(4-chloro-2-methylenebutanamido) benzoate (133.86 mg, 0.5 mmol, 1.0 equiv.), NHC-3 (301.1 mg, 0.65 mmol, 1.3 equiv.), pyridine (63.28 mg, 0.8 mmol, 1.6 equiv.), 3-methyloxetan-3-ol (57.30 mg, 0.65 mmol, 1.3 equiv.).

Purification by flash column chromatography (30% EtOAc/pentane) gave the title compound (136.51 mg, 0.45 mmol, 90%) as a yellow sticky oil.

**TLC:** R_f_ = 0.50 (50% EtOAc/pentane, KMnO_4_ stain).

**^1^H NMR** (400 MHz, CDCl_3_) *δ* 8.04 – 7.94 (m, 2H), 7.78 – 7.67 (m, 1H), 7.62 – 7.50 (m, 2H), 4.52 (d, *J* = 5.8 Hz, 2H), 4.31 (d, *J* = 5.8 Hz, 2H), 3.89 (s, 3H), 2.04 (s, 2H), 1.48 (s, 3H), 1.20 – 1.11 (m, 2H), 0.77 – 0.69 (m, 2H).

**^13^C NMR** (101 MHz, CDCl_3_) *δ* 171.97, 166.62, 142.05, 130.86, 125.67, 118.95, 83.24, 52.05, 44.37, 40.33, 24.82, 23.77, 13.18.

**HRMS** (ESI^+^) calcd for C_17_H_21_NO_4_ [M+H] ^+^ 304.1543, found 304.1546.

**Methyl 4-(1-((1-methylcyclopentyl) methyl) cyclopropane-1-carboxamido) benzoate (6ac)**

The title compound was prepared according to General Procedure C with 4CzTPN (4.0 mg, 5.0 µmol, 0.01 equiv.), NaOAc (82.03 mg, 1.0 mmol, 2.0 equiv.), Methyl 4-(4-chloro-2-methylenebutanamido) benzoate (133.86 mg, 0.5 mmol, 1.0 equiv.), NHC-3 (301.1 mg, 0.65 mmol, 1.3 equiv.), pyridine (63.28 mg, 0.8 mmol, 1.6 equiv.), 1-methylcyclopentan-1-ol (65.11 mg, 0.65 mmol, 1.3 equiv.).

Purification by flash column chromatography (5% EtOAc/pentane) gave the title compound (134.05 mg, 0.425 mmol, 85%) as a colorless sticky oil.

**TLC:** R_f_ = 0.50 (15% EtOAc/pentane, KMnO_4_ stain).

**^1^H NMR** (400 MHz, CDCl_3_) *δ* 8.01 – 7.96 (m, 2H), 7.78 (s, 1H), 7.63 – 7.58 (m, 2H), 3.89 (s, 3H), 1.65 – 1.55 (m, 4H), 1.41 (tt, *J* = 6.1, 3.3 Hz, 4H), 1.18 – 1.13 (m, 2H), 1.01 (s, 3H), 0.75 – 0.67 (m, 2H).

**^13^C NMR** (101 MHz, CDCl_3_) *δ* 172.88, 166.69, 142.36, 130.85, 125.34, 118.76, 51.98, 47.87, 44.16, 40.05, 25.34, 25.29, 23.50, 13.48.

**HRMS** (ESI^+^) calcd for C_19_H_25_NO_3_ [M+H] ^+^ 316.1907, found 316.1906.

**(1R,3S,7R,8R,8aS)-3,7-dimethyl-8-(2-((2R,4S)-6-oxo-4-((1-(phenylcarbamoyl) cyclopropyl) methyl) tetrahydro-2H-pyran-2-yl) ethyl)-1,2,3,7,8,8a-hexahydronaphthalen-1-yl 2,2-dimethylbutanoate (7)**

The title compound was prepared according to General Procedure B with 4CzTPN (4.0 mg, 5.0 µmol, 0.01 equiv.), NaOAc (82.03 mg, 1.0 mmol, 2.0 equiv.), 4-chloro-2-methylene-N-phenylbutanamide (104.83 mg, 0.5 mmol, 1.0 equiv.), NHC-1 (256.91 mg, 0.65 mmol, 1.3 equiv.), pyridine (63.28 mg, 0.8 mmol, 1.6 equiv.), Simvastatin (272.12 mg, 0.65 mmol, 1.3 equiv.).

Purification by flash column chromatography (20% EtOAc/pentane) gave the title compound (244.71 mg, 0.425 mmol, 85%) as a yellow oil.

**TLC:** R_f_ = 0.50 (50% EtOAc/pentane, KMnO_4_ stain).

**^1^H NMR** (400 MHz, CDCl_3_) *δ* 7.61 (d, *J* = 4.7 Hz, 1H), 7.56 – 7.47 (m, 2H), 7.40 – 7.27 (m, 2H), 7.10 (td, *J* = 7.3, 1.3 Hz, 1H), 5.97 (d, *J* = 9.7 Hz, 1H), 5.76 (dd, *J* = 9.7, 6.0 Hz, 1H), 5.49 (t, *J* = 3.2 Hz, 1H), 5.37 – 5.28 (m, 1H), 4.27 (dq, *J* = 11.3, 5.1 Hz, 1H), 2.84 – 2.59 (m, 1H), 2.44 (d, *J* = 11.0 Hz, 1H), 2.39 – 2.30 (m, 2H), 2.24 – 2.06 (m, 1H), 1.96 – 1.85 (m, 3H), 1.85 – 1.73 (m, 3H), 1.73 – 1.59 (m, 2H), 1.58 – 1.41 (m, 3H), 1.37 – 1.17 (m, 5H), 1.10 (dt, *J* = 11.6, 6.2 Hz, 9H), 0.91 – 0.79 (m, 6H), 0.78 – 0.69 (m, 2H).

**^13^C NMR** (101 MHz, CDCl_3_) *δ* 178.06, 171.96, 171.57, 137.83, 132.91, 131.46, 129.70, 128.99, 128.37, 124.47, 120.24, 120.09, 119.89, 77.96, 67.96, 43.12, 41.01, 37.54, 37.45, 36.56, 36.49, 35.88, 33.08, 33.04, 32.90, 30.61, 28.33, 27.27, 25.00, 24.91, 24.83, 24.63, 24.41, 23.12, 13.92, 13.87, 13.08, 9.44.

**HRMS** (ESI^+^) calcd for C_36_H_49_NO_5_ [M+H] ^+^ 576.3683, found 576.3680.

***N*-phenyl-1-(((2S,3S,4R,5R,6R)-3,4,5-tris(benzyloxy)-6-((benzyloxy) methyl) tetrahydro-2*H*-pyran-2-yl) methyl) cyclopropane-1-carboxamide (8)**

The title compound was prepared according to General Procedure B using CPME/DMSO in place of MTBE/DMA, 4CzTPN (4.0 mg, 5.0 µmol, 0.01 equiv.), NaOAc (82.03 mg, 1.0 mmol, 2.0 equiv.), 4-chloro-2-methylene-N-phenylbutanamide (104.83 mg, 0.5 mmol, 1.0 equiv.), NHC-1 (256.91 mg, 0.65 mmol, 1.3 equiv.), pyridine (63.28 mg, 0.8 mmol, 1.6 equiv.), D-glucopyranose (351.43 mg, 0.65 mmol, 1.3 equiv.).

Purification by flash column chromatography (10% EtOAc/pentane) gave the title compound (261.71 mg, 0.375 mmol, 75%) as a yellow oil.

**TLC:** R_f_ = 0.50 (30% EtOAc/pentane, KMnO_4_ stain).

**^1^H NMR** (400 MHz, CDCl_3_) *δ* 8.75 (s, 1H), 7.47 (d, *J* = 8.0 Hz, 2H), 7.36 – 7.20 (m, 21H), 7.12 (dd, *J* = 6.6, 2.9 Hz, 2H), 7.04 (t, *J* = 7.4 Hz, 1H), 4.87 – 4.71 (m, 4H), 4.63 (d, *J* = 11.6 Hz, 1H), 4.47 (dd, *J* = 11.6, 2.1 Hz, 2H), 4.40 – 4.29 (m, 2H), 3.84 (ddd, *J* = 8.9, 4.7, 2.6 Hz, 1H), 3.69 – 3.61 (m, 3H), 3.60 – 3.53 (m, 2H), 2.37 (dd, *J* = 16.1, 8.4 Hz, 1H), 1.61 (dd, *J* = 16.1, 2.1 Hz, 1H), 1.35 (ddd, *J* = 10.2, 6.4, 3.9 Hz, 1H), 1.15 (ddd, *J* = 10.1, 6.2, 3.9 Hz, 1H), 0.73 (ddd, *J* = 10.1, 6.3, 4.0 Hz, 1H), 0.61 (ddd, *J* = 9.7, 6.5, 3.9 Hz, 1H).

**^13^C NMR** (101 MHz, CDCl_3_) *δ* 172.41, 138.66, 138.43, 138.02, 137.81, 137.78, 128.79, 128.61, 128.54, 128.50, 128.47, 128.09, 128.02, 127.97, 127.90, 127.83, 123.97, 120.54, 81.51, 79.00, 75.17, 74.88, 74.62, 73.65, 73.53, 72.92, 68.86, 31.18, 25.51, 16.72, 14.57.

**HRMS** (ESI^+^) calcd for C_45_H_47_NO_6_ [M+H] ^+^ 698.3476, found 698.3471.

***N*-phenyl-1-(((3aS,5aR,8aR,8bS)-2,2,7,7-tetramethyltetrahydro-3a*H-*bis ([1,3] dioxolo) [4,5-b:4',5'-d] pyran-3a-yl) methyl) cyclopropane-1-carboxamide (9)**

The title compound was prepared according to General Procedure A with 4CzTPN (4.0 mg, 5.0 µmol, 0.01 equiv.), NaOAc (82.03 mg, 1.0 mmol, 2.0 equiv.), 4-chloro-2-methylene-N-phenylbutanamide (104.83 mg, 0.5 mmol, 1.0 equiv.), NHC-2 (276.42 mg, 0.65 mmol, 1.3 equiv.), pyridine (63.28 mg, 0.8 mmol, 1.6 equiv.), ß-D-fructopyranose (169.19 mg, 0.65 mmol, 1.3 equiv.).

Purification by flash column chromatography (5% EtOAc/pentane) gave the title compound (145.95 mg, 0.35 mmol, 70%) as a yellow oil.

**TLC:** R_f_ = 0.50 (15% EtOAc/pentane, KMnO_4_ stain).

**^1^H NMR** (400 MHz, CDCl_3_) *δ* 8.17 (s, 1H), 7.58 – 7.53 (m, 2H), 7.28 (d, *J* = 7.8 Hz, 2H), 7.09 – 7.02 (m, 1H), 4.57 (dd, *J* = 8.0, 2.4 Hz, 1H), 4.24 (dd, *J* = 8.1, 1.7 Hz, 1H), 4.08 (d, *J* = 2.4 Hz, 1H), 3.94 – 3.78 (m, 2H), 2.17 – 2.05 (m, 2H), 1.97 (ddd, *J* = 14.4, 10.2, 5.4 Hz, 1H), 1.88 – 1.79 (m, 1H), 1.53 (s, 3H), 1.30 (dt, *J* = 5.6, 2.5 Hz, 11H), 0.67 (q, *J* = 3.6 Hz, 2H)

**^13^C NMR** (101 MHz, CDCl_3_) *δ* 172.47, 138.50, 128.77, 123.88, 120.08, 108.97, 107.76, 103.79, 74.15, 70.66, 70.43, 61.12, 38.73, 27.20, 26.38, 25.68, 25.00, 24.72, 23.83, 16.05, 15.37.

**HRMS** (ESI^+^) calcd for C_22_H_29_NO_6_ [M+H] ^+^ 404.2067, found 404.2066.

**(R)-4-((3-chloro-4-methoxybenzyl) amino)-2-(2-(2-(1-(phenylcarbamoyl) cyclopropyl) ethyl) pyrrolidin-1-yl)-*N*-(pyrimidin-2-ylmethyl) pyrimidine-5-carboxamide (10)**

The title compound was prepared according to General Procedure B using 1,4-Dioxane in place of MTBE, 4CzTPN (4.0 mg, 5.0 µmol, 0.01 equiv.), NaOAc (82.03 mg, 1.0 mmol, 2.0 equiv.), 4-chloro-2-methylene-N-phenylbutanamide (104.83 mg, 0.5 mmol, 1.0 equiv.), NHC-2 (276.42 mg, 0.65 mmol, 1.3 equiv.), pyridine (63.28 mg, 0.8 mmol, 1.6 equiv.), Avanafil (427.52 mg, 0.65 mmol, 1.3 equiv.).

Purification by flash column chromatography (70% EtOAc/pentane) gave the title compound (128.23 mg, 0.20 mmol, 40%, unassigned 1:0.3 d.r.) as a yellow sticky oil.

**TLC:** R_f_ = 0.40 (100% EtOAc, KMnO_4_ stain).

**^1^H NMR** (400 MHz, CDCl_3_) *δ* 11.19 (s, 1H), 9.28 (dt, *J* = 18.0, 5.7 Hz, 1H), 8.67 (dd, *J* = 7.9, 4.9 Hz, 2H), 8.37 (d, *J* = 12.9 Hz, 1H), 7.72 – 7.62 (m, 2H), 7.45 – 7.31 (m, 4H), 7.28 – 7.20 (m, 2H), 7.16 – 7.07 (m, 1H), 6.90 (d, *J* = 8.5 Hz, 1H), 4.75 (dd, *J* = 7.1, 4.5 Hz, 2H), 4.70 – 4.26 (m, 4H), 3.89 (s, 3H), 3.77 (dd, *J* = 10.6, 6.6 Hz, 1H), 3.63 (dd, *J* = 10.6, 4.7 Hz, 1H), 3.06 (d, *J* = 15.1 Hz, 1H), 2.27 – 1.97 (m, 3H), 1.91 – 1.75 (m, 1H), 1.65 – 1.46 (m, 1H), 1.34 – 1.08 (m, 2H), 0.98 – 0.74 (m, 2H), 0.63 – 0.47 (m, 1H) (summary of all rotamers and diastereomers).

**^13^C NMR** (101 MHz, CDCl_3_) *δ* 173.01, 172.77, 166.97, 166.90, 165.59, 161.13, 161.01, 159.85, 157.23, 155.11, 154.96, 154.24, 139.50, 139.33, 131.62, 131.34, 129.65, 129.50, 128.82, 126.96, 126.87, 124.09, 124.01, 122.45, 122.07, 121.94, 119.73, 112.17, 100.06, 99.39, 67.84, 66.44, 61.48, 60.47, 59.59, 58.47, 57.51, 56.25, 45.00, 44.96, 43.76, 43.61, 39.69, 35.39, 32.18, 31.48, 30.23, 29.75, 28.61, 22.74, 22.36, 21.12, 20.69, 17.78, 16.99, 14.99, 14.25, 14.19 (summary of all rotamers and diastereomers).

**HRMS** (ESI^+^) calcd. for C_34_H_37_ClN_8_O_3_ [M+H] ^+^ 641.2750, found 641.2750.

**1-(((2S,3S,5R)-2-((bis(4-methoxyphenyl) (phenyl) methoxy) methyl)-5-(5-methyl-2,4-dioxo-3,4-dihydropyrimidin-1(2*H*)-yl) tetrahydrofuran-3-yl) methyl)-*N*-phenylcyclopropane-1-carboxamide (11)**

The title compound was prepared according to General Procedure B with 4CzTPN (4.0 mg, 5.0 µmol, 0.01 equiv.), NaOAc (82.03 mg, 1.0 mmol, 2.0 equiv.), 4-chloro-2-methylene-N-phenylbutanamide (104.83 mg, 0.5 mmol, 1.0 equiv.), NHC-1 (256.91 mg, 0.65 mmol, 1.3 equiv.), pyridine (63.28 mg, 0.8 mmol, 1.6 equiv.), DMT-thymidine (354.3 mg, 0.65 mmol, 1.3 equiv.).

Purification by flash column chromatography (50% EtOAc/pentane) gave the title compound (259.47 mg, 0.37 mmol, 74%, unassigned 1:0.36 d.r.) as a yellow sticky oil.

**TLC:** R_f_ = 0.50 (80% EtOAc/pentane, KMnO_4_ stain).

**^1^H NMR** (400 MHz, CDCl_3_) *δ* 9.26 (d, *J* = 27.6 Hz, 1H), 7.67 (d, *J* = 1.4 Hz, 1H), 7.48 – 7.34 (m, 3H), 7.30 (tt, *J* = 11.7, 5.1 Hz, 9H), 7.24 – 7.17 (m, 1H), 7.15 – 7.07 (m, 1H), 6.96 – 6.70 (m, 4H), 6.07 (ddd, *J* = 28.4, 7.1, 2.3 Hz, 1H), 3.83 – 3.77 (m, 6H), 3.60 (dd, *J* = 10.8, 2.7 Hz, 1H), 2.70 (d, *J* = 39.7 Hz, 1H), 2.52 – 2.34 (m, 1H), 2.23 (ddd, *J* = 13.8, 10.3, 7.2 Hz, 1H), 2.16 – 2.02 (m, 1H), 2.00 – 1.89 (m, 1H), 1.52 – 1.38 (m, 3H), 1.35 – 1.13 (m, 2H), 1.11 – 0.61 (m, 2H) (summary of all rotamers and diastereomers).

**^13^C NMR** (101 MHz, CDCl_3_) *δ* 172.31, 171.32, 164.08, 158.66, 150.44, 150.37, 147.39, 144.23, 139.53, 137.64, 136.49, 135.82, 135.43, 130.19, 130.14, 129.20, 129.06, 128.29, 128.03, 127.90, 127.83, 127.19, 127.13, 124.72, 124.57, 120.41, 120.31, 113.28, 113.21, 110.49, 110.13, 86.72, 86.48, 85.41, 85.35, 85.28, 81.49, 62.72, 61.61, 60.49, 55.31, 40.60, 39.80, 37.43, 37.26, 36.95, 35.75, 29.77, 25.40, 25.25, 14.95, 14.26, 13.89, 13.81, 13.43, 12.60, 11.95 (summary of all rotamers and diastereomers).

**HRMS** (ESI^+^) calcd. for C_42_H_43_N_3_O_7_ [M+ Na] ^+^ 724.2999, found 724.2999.

***N*-(9-((4S)-5-((bis(4-methoxyphenyl) (phenyl) methoxy) methyl)-4-((1-(phenylcarbamoyl) cyclopropyl) methyl) tetrahydrofuran-2-yl)-9*H*-purin-6-yl) benzamide (12)**

The title compound was prepared according to General Procedure B with 4CzTPN (4.0 mg, 5.0 µmol, 0.01 equiv.), NaOAc (82.03 mg, 1.0 mmol, 2.0 equiv.), 4-chloro-2-methylene-N-phenylbutanamide (104.83 mg, 0.5 mmol, 1.0 equiv.), NHC-1 (256.91 mg, 0.65 mmol, 1.3 equiv.), pyridine (63.28 mg, 0.8 mmol, 1.6 equiv.), DMT-adenosine (427.52 mg, 0.65 mmol, 1.3 equiv.).

Purification by flash column chromatography (80% EtOAc/pentane) gave the title compound (244.47 mg, 0.30 mmol, 60%) as a yellow sticky oil.

**TLC:** R_f_ = 0.40 (100% EtOAc, KMnO_4_ stain).

**^1^H NMR** (400 MHz, CDCl_3_) *δ* 9.26 (s, 1H), 8.73 (s, 1H), 8.27 (s, 1H), 8.05 (d, *J* = 7.6 Hz, 2H), 7.62 (t, *J* = 7.4 Hz, 1H), 7.53 (t, *J* = 7.6 Hz, 2H), 7.42 – 7.36 (m, 2H), 7.33 – 7.18 (m, 12H), 7.11 (t, *J* = 7.2 Hz, 1H), 6.78 (d, *J* = 8.7 Hz, 4H), 6.39 (d, *J* = 6.8 Hz, 1H), 4.01 (dt, *J* = 8.5, 4.2 Hz, 1H), 3.78 (s, 6H), 3.49 – 3.33 (m, 2H), 3.00 (q, *J* = 9.6, 8.4 Hz, 2H), 2.40 (dt, *J* = 11.9, 9.3 Hz, 1H), 1.64 (dd, *J* = 14.6, 9.1 Hz, 1H), 1.27 (dt, *J* = 9.7, 5.1 Hz, 2H), 1.10 (dt, *J* = 10.3, 5.0 Hz, 1H), 0.75 (ddq, *J* = 19.7, 9.5, 5.1 Hz, 2H).

^13^C NMR (101 MHz, CDCl_3_) *δ* 171.30, 164.76, 158.56, 152.56, 150.96, 149.49, 144.36, 141.50, 137.49, 135.61, 135.56, 133.82, 132.74, 130.04, 130.00, 129.19, 129.04, 129.00, 128.84, 128.15, 127.97, 127.88, 127.83, 127.02, 124.58, 123.32, 120.33, 120.26, 113.23, 113.18, 86.64, 85.53, 85.31, 63.87, 55.28, 39.16, 38.28, 37.38, 29.76, 25.29, 14.15, 13.75.

**HRMS** (ESI^+^) calcd for C_49_H_46_N_6_O_6_ [M+H] ^+^ 815.3551, found 815.3547.

**1-(((3S,8S,9S,10R,13R,14S,17R)-10,13-dimethyl-17-((R)-6-methylheptan-2-yl)-2,3,4,7,8,9,10,11,12,13,14,15,16,17-tetradecahydro-1*H*-cyclopenta [a] phenanthren-3-yl) methyl)-*N*-phenylcyclopropane-1-carboxamide (13)**

The title compound was prepared according to General Procedure B with 4CzTPN (4.0 mg, 5.0 µmol, 0.01 equiv.), NaOAc (82.03 mg, 1.0 mmol, 2.0 equiv.), 4-chloro-2-methylene-N-phenylbutanamide (104.83 mg, 0.5 mmol, 1.0 equiv.), NHC-1 (256.91 mg, 0.65 mmol, 1.3 equiv.), pyridine (63.28 mg, 0.8 mmol, 1.6 equiv.), dihydrocholesterol (251.33 mg, 0.65 mmol, 1.3 equiv.).

Purification by flash column chromatography (2.5% EtOAc/pentane) gave the title compound (231.15 mg, 0.425 mmol, 85%, (**+**) 13: (**-**) 13 = 2:1 d.r., (**+**) 13: [α]25 D= +13.7, (**-**) 13: [α]25 D= -58.0) as a yellow solid, m.p.: 107.2-109.1℃.

**TLC:** R_f_ = 0.40 (10% EtOAc/pentane, KMnO_4_ stain).

**^1^H NMR** (400 MHz, CDCl_3_) *δ* 7.49 (d, *J* = 8.0 Hz, 2H), 7.37 (s, 1H), 7.31 (t, *J* = 7.8 Hz, 2H), 7.09 (t, *J* = 7.4 Hz, 1H), 5.29 (d, *J* = 4.6 Hz, 1H), 2.17 – 2.10 (m, 1H), 2.04 – 1.90 (m, 3H), 1.87 – 1.71 (m, 3H), 1.65 – 1.46 (m, 7H), 1.46 – 1.30 (m, 5H), 1.29 – 1.19 (m, 5H), 1.19 – 1.07 (m, 5H), 1.07 – 0.98 (m, 3H), 0.96 (s, 4H), 0.91 (d, *J* = 6.5 Hz, 4H), 0.87 – 0.83 (m, 5H), 0.67 (d, *J* = 6.1 Hz, 5H).

**^13^C NMR** (101 MHz, CDCl_3_) *δ* 172.37, 140.27, 138.15, 128.98, 124.08, 121.97, 119.86, 56.94, 56.30, 50.60, 42.37, 39.89, 39.58, 37.39, 36.79, 36.57, 36.27, 35.86, 34.17, 33.65, 31.92, 31.87, 28.29, 28.06, 27.00, 25.14, 24.31, 23.94, 22.87, 22.62, 20.79, 19.51, 18.79, 14.17, 13.78, 11.91.

**HRMS** (ESI^+^) calcd for C_38_H_57_NO [M+H] ^+^ 544.4512, found 544.4518.

**1-(((2R,4aR,4bS,6aS,7aS,8aS,8bS,8cR,8dR,9aR,9bR)-9b-hydroxy-4a,6a-dimethyl-7-oxooctadecahydro-1*H*-cyclopropa [4,5] cyclopenta [1,2-a] cyclopropa [l] phenanthren-2-yl) methyl)-*N*-phenylcyclopropane-1-carboxamide (14)**

The title compound was prepared according to General Procedure B with 4CzTPN (4.0 mg, 5.0 µmol, 0.01 equiv.), NaOAc (82.03 mg, 1.0 mmol, 2.0 equiv.), 4-chloro-2-methylene-N-phenylbutanamide (104.83 mg, 0.5 mmol, 1.0 equiv.), NHC-1 (256.91 mg, 0.65 mmol, 1.3 equiv.), pyridine (63.28 mg, 0.8 mmol, 1.6 equiv.), drospiroone (214.81 mg, 0.65 mmol, 1.3 equiv.).

Purification by flash column chromatography (25% EtOAc/pentane) gave the title compound (219.45 mg, 0.45 mmol, 90%, (**+**) 14: (**-**) 14 = 2:1 d.r., (**+**) 14: [α]25 D= +43.3, (**-**) 13: [α]25 D= -64.7) as a yellow sticky oil.

**TLC:** R_f_ = 0.40 (50% EtOAc/pentane, KMnO_4_ stain).

**^1^H NMR** (400 MHz, CDCl_3_) *δ* 7.59 – 7.48 (m, 3H), 7.37 – 7.29 (m, 2H), 7.10 (t, *J* = 7.4 Hz, 1H), 3.48 (s, 1H), 2.25 – 2.12 (m, 2H), 2.10 – 1.86 (m, 4H), 1.86 – 1.74 (m, 3H), 1.73 – 1.63 (m, 3H), 1.63 – 1.51 (m, 2H), 1.50 – 1.40 (m, 2H), 1.35 (ddd, *J* = 19.3, 11.0, 5.0 Hz, 2H), 1.30 – 1.24 (m, 2H), 1.19 (ddd, *J* = 12.5, 7.6, 3.3 Hz, 5H), 0.95 (s, 3H), 0.89 (dt, *J* = 8.9, 4.2 Hz, 2H), 0.80 (s, 3H), 0.71 (d, *J* = 2.6 Hz, 2H).

**^13^C NMR** (101 MHz, CDCl_3_) *δ* 216.48, 172.46, 138.05, 128.96, 124.21, 120.24, 73.14, 52.05, 46.31, 45.63, 43.02, 42.29, 40.14, 35.04, 33.64, 33.51, 33.00, 27.52, 26.09, 25.93, 24.39, 22.50, 20.81, 20.19, 18.86, 17.74, 14.62, 14.47, 14.40, 11.79.

**HRMS** (ESI^+^) calcd for C_32_H_41_NO_3_ [M+H] ^+^ 488.3159, found 488.3160.

***N*-(2-(2,6-dioxopiperidin-3-yl)-1-oxoisoindolin-4-yl)-1-isobutylcyclopropane-1-carboxamide (15)**

The title compound was prepared according to General Procedure B with 4CzTPN (4.0 mg, 5.0 µmol, 0.01 equiv.), NaOAc (82.03 mg, 1.0 mmol, 2.0 equiv.), 4-chloro-N-(2-(2,6-dioxopiperidin-3-yl)-1-oxoisoindolin-4-yl)-2-methylenebutanamide (187.55 mg, 0.5 mmol, 1.0 equiv.), NHC-1 (256.91 mg, 0.65 mmol, 1.3 equiv.), pyridine (63.28 mg, 0.8 mmol, 1.6 equiv.), propan-2-ol (39.1 mg, 0.65 mmol, 1.3 equiv.).

Purification by flash column chromatography (2% MeOH/CH_2_Cl_2_) gave the title compound (90 mg, 0.24 mmol, 47 %) as a white sticky oil.

**TLC:** R_f_ = 0.40 (5% MeOH/CH_2_Cl_2_, KMnO_4_ stain).

**^1^H NMR** (400 MHz, CDCl_3_) *δ* 8.88 (s, 1H), 7.93 (s, 1H), 7.71 (d, *J* = 7.4 Hz, 1H), 7.58 (d, *J* = 7.8 Hz, 1H), 7.47 (t, *J* = 7.5 Hz, 1H), 5.09 (dd, *J* = 13.4, 5.1 Hz, 1H), 4.35 (t, *J* = 6.5 Hz, 2H), 2.85 – 2.63 (m, 2H), 2.13 (dp, *J* = 10.2, 3.6, 2.9 Hz, 1H), 1.95 (p, *J* = 6.9 Hz, 2H), 1.72 – 1.52 (m, 2H), 1.29 – 1.21 (m, 2H), 1.00 (t, *J* = 6.2 Hz, 6H), 0.72 (d, *J* = 2.7 Hz, 2H).

**^13^C NMR** (101 MHz, CDCl_3_) *δ* 173.04, 171.60, 170.15, 169.08, 135.15, 132.99, 132.79, 129.03, 126.91, 121.17, 51.87, 51.71, 46.69, 46.37, 43.91, 43.47, 42.64, 41.79, 41.70, 35.50, 31.55, 31.48, 30.18, 29.74, 27.86, 26.11, 24.53, 23.17, 23.12, 23.05, 22.45, 14.76, 14.62.

**HRMS** (ESI^+^) calcd for C_21_H_25_N_3_O_4_ [M+H] ^+^ 384.1917, found 384.1913.

**2-(4-(3-(2-chloro-10H-phenothiazin-10-yl)propyl)piperazin-1-yl)ethyl 1-isobutylcyclopropane-1-carboxylate (16)**

The title compound was prepared according to General Procedure B with 4CzTPN (4.0 mg, 5.0 µmol, 0.01 equiv.), NaOAc (82.03 mg, 1.0 mmol, 2.0 equiv.), 2-(4-(3-(2-chloro-10*H*-phenothiazin-10-yl)propyl)piperazin-1-yl)ethyl 4-chloro-2-methylenebut-anoate (259.58 mg, 0.5 mmol, 1.0 equiv.), NHC-1 (256.91 mg, 0.65 mmol, 1.3 equiv.), pyridine (63.28 mg, 0.8 mmol, 1.6 equiv.), propan-2-ol (39.1 mg, 0.65 mmol, 1.3 equiv.).

Purification by flash column chromatography (2% MeOH/CH_2_Cl_2_) gave the title compound (220 mg, 0.42mmol, 84%) as a yellow oil.

**TLC:** R_f_ = 0.40 (5% MeOH/CH_2_Cl_2_, KMnO_4_ stain).

**^1^H NMR** (400 MHz, CDCl_3_) *δ* 7.22 – 7.14 (m, 2H), 7.05 (d, *J* = 8.1 Hz, 1H), 6.97 (td, *J* = 7.5, 1.2 Hz, 1H), 6.92 (dt, *J* = 8.1, 1.7 Hz, 2H), 6.88 (d, *J* = 2.1 Hz, 1H), 4.20 (t, *J* = 5.9 Hz, 2H), 3.94 (t, *J* = 6.8 Hz, 2H), 2.64 (t, *J* = 5.9 Hz, 2H), 2.58 (s, 8H), 2.05 – 1.99 (m, 2H), 1.98 – 1.92 (m, 1H), 1.47 (d, *J* = 7.2 Hz, 2H), 1.39 (d, *J* = 15.4 Hz, 1H), 1.32 (s, 1H), 1.24 – 1.20 (m, 2H), 0.92 (d, *J* = 6.6 Hz, 6H), 0.71 (q, J = 4.0 Hz, 2H).

**^13^C NMR** (101 MHz, CDCl_3_) *δ* 175.22, 146.46, 144.44, 133.27, 127.92, 127.54, 127.46, 124.92, 123.65, 122.97, 122.33, 115.91, 115.88, 61.81, 56.49, 55.41, 53.14, 45.27, 42.73, 31.51, 30.15, 27.30, 22.97, 22.29, 15.62.

**HRMS** (ESI^+^) calcd for C_29_H_38_ClN_3_O_2_S [M+H] ^+^ 528.2442, found 528.2442.

# 6. Mechanistic Studies

**Methyl 2-(acetoxy(phenyl) methyl) acrylate (24)**

Substrate **24** was prepared following a literature procedure.^10^ All recorded spectroscopic data matched those previously reported in the literature.

**TLC:** R_f_ = 0.40 (5% EtOAc/pentane, KMnO_4_ stain).

^1^H NMR (400 MHz, CDCl_3_) *δ* 7.45 – 7.31 (m, 5H), 6.72 (s, 1H), 6.44 (t, *J* = 0.9 Hz, 1H), 5.90 (dd, *J* = 1.5, 0.9 Hz, 1H), 3.74 (s, 3H), 2.14 (s, 3H).

**LCMS** (ESI^+^) calcd. for C_13_H_14_O_4_ [M+H] ^+^ 235.1, found 235.1.

**Benzyl (*E*)-4-(2-(methoxycarbonyl)-3-phenylallyl) piperidine-1-carboxylate (*E*-25)**

The title compound was prepared according to General Procedure B with 4CzTPN (4.0 mg, 5.0 µmol, 0.01 equiv.), NaOAc (82.03 mg, 1.0 mmol, 2.0 equiv.), Methyl 2-(acetoxy(phenyl) methyl) acrylate (117.25 mg, 0.5 mmol, 1.0 equiv.), NHC-1 (256.91 mg, 0.65 mmol, 1.3 equiv.), pyridine (63.28 mg, 0.8 mmol, 1.6 equiv.), benzyl 4-hydroxypiperidine-1-carboxylate (152.93 mg, 0.65 mmol, 1.3 equiv.).

Purification by flash column chromatography (3% EtOAc/pentane) gave the title compound (188.87 mg, 0.39 mmol, 96%, *E*/*Z* = 2:3) as a colorless oil.

**TLC:** R_f_ = 0.50 (10% EtOAc/pentane, KMnO_4_ stain).

**^1^H NMR** (400 MHz, CDCl_3_) *δ* 7.78 (s, 1H), 7.44 – 7.33 (m, 10H), 5.12 (s, 2H), 4.11 (d, *J* = 10.4 Hz, 2H), 3.85 (s, 3H), 2.73 (t, *J* = 17.3 Hz, 2H), 2.57 (d, *J* = 7.0 Hz, 2H), 1.74 (s, 1H), 1.65 (d, *J* = 12.0 Hz, 2H), 1.09 (q, *J* = 11.0, 9.9 Hz, 2H).

**^13^C NMR** (101 MHz, CDCl_3_) *δ* 168.96, 155.21, 140.50, 136.95, 135.73, 131.52, 129.05, 128.57, 128.46, 128.37, 127.91, 127.84, 66.95, 52.07, 44.12, 35.86, 33.40, 31.90.

**HRMS** (ESI^+^) calcd for C_24_H_27_NO_4_ [M+H] ^+^ 394.2013, found 394.2011.

**Benzyl (E)-4-(2-(methoxycarbonyl)-3-phenylallyl) piperidine-1-carboxylate (*Z*-25)**

**TLC:** R_f_ = 0.50 (10% EtOAc/pentane, KMnO_4_ stain).

**^1^H NMR** (400 MHz, CDCl_3_) *δ* 7.40 (d, *J* = 4.4 Hz, 4H), 7.37 – 7.30 (m, 4H), 7.27 – 7.23 (m, 2H), 6.65 (s, 1H), 5.16 (s, 2H), 4.23 (t, *J* = 15.5 Hz, 2H), 3.68 (s, 3H), 2.78 (d, *J* = 18.2 Hz, 2H), 2.39 (d, *J* = 7.0 Hz, 2H), 1.80 (d, *J* = 13.0 Hz, 2H), 1.65 (s, 1H), 1.19 (d, *J* = 12.7 Hz, 2H).

**^13^C NMR** (101 MHz, CDCl_3_) *δ* 170.21, 155.29, 136.97, 135.89, 135.03, 132.23, 128.48, 128.22, 128.02, 127.93, 127.89, 127.84, 66.98, 51.69, 44.13, 42.66, 35.03, 31.78.

**HRMS** (ESI^+^) calcd for C_24_H_27_NO_4_ [M+H] ^+^ 394.2013, found 394.2010.

# 7. References cited

[1] A. B. Pangborn, M. A. Giardello, R. H. Grubbs, R. K. Rosen, F. J Timmers, *Organometallics.* **1996**, *15*, 1518–1520.

[2] Z. Dong; D. W. C. MacMillan. *Nature.* **2021**, *598*, 451−456.

[3] M.-C. Fu, R. Shang, W.-M. Cheng, Y. Fu, *ACS Catal*. **2016**, *6*, 2501–2505.

[4] M. Morita, L. Drouin, R. Motoki, Y. Kimura, I. Fujimori, M. Kanai, M. Shibasaki, *J. Am. Chem. Soc.* **2009**, *131*, 11, 3858–3859.

[5] M. Lachia, S. Iriart, M. Baalouch, A. De Mesmaeker, R. Beaudegnies, *Tetrahedron Lett*. **2011**, *52*, 3219-3222.

[6] C. Shu, R. S. Mega, B. J. Andreassen, A. Noble, V. K. Aggarwal, *Angew. Chem. Int. Ed.* **2018**, *57*, 15430-15434.

[7] V. P. Ananikov, L. L. Khemchyan, I. P. Beletskaya, *Synlett*. **2009**, *15*, 2375-2381.

[8] C. H. Oh, H. H. Jung, K. S. Kim, N. Kim, *Angew. Chem. Int. Ed.* **2003**, *42*, 805-808.

[9] D. Janssen-Müller, M. Schedler, M. Fleige, C. G. Daniliuc, F. Glorius, *Angew. Chem. Int. Ed.* **2015**, *54*, 12492-12496.

[10] D. C. Braddock, R. H. Pouwer, J. W. Burton, P. Broadwith, *J. Org. Chem.* **2009**, *74*, 6042-6049.

[11] T. den Hartog, A. Rudolph, B. Maciá, A. J. Minnaard, B. L. Feringa, *J. Am. Chem. Soc.* **2010**, *132*, 14349–14351.

[12] X. Zhang, Y. Zhang, X. Li, B. Li, S. Xiao, Y. Tang, P. Xie, T.-P. Loh, *Org. Lett.* **2023**, *25*, 6863-6868.

# 8. Copies of NMR Spectra

^1^H NMR (400 MHz, CDCl_3_) of **2a**


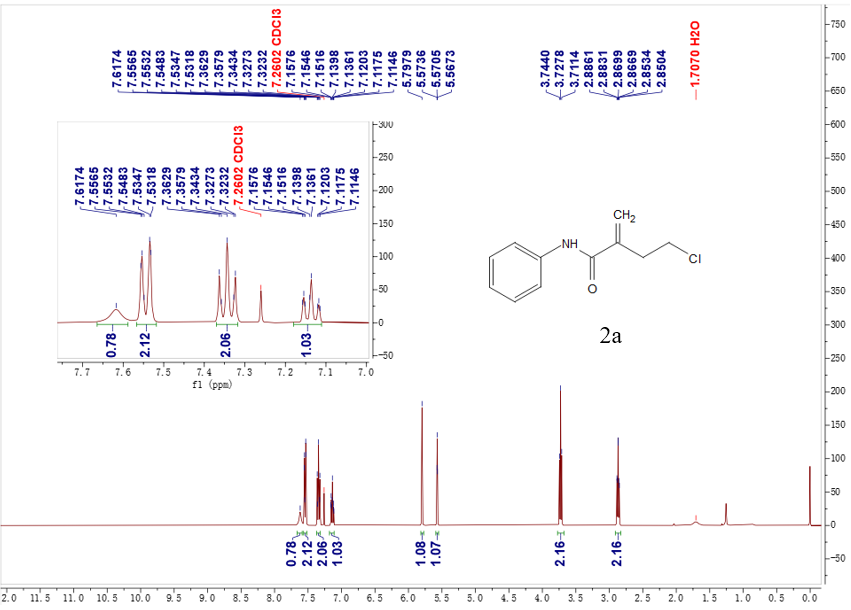


^13^C NMR (101 MHz, CDCl_3_) of **2a**


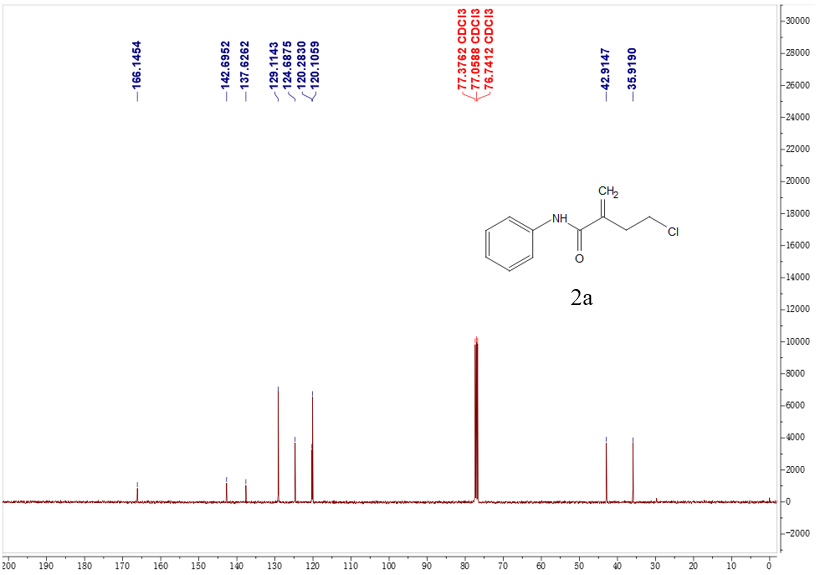


HRMS of **2a** (calcd for C_11_H_12_ClNO [M+H] ^+^ 210.0680)


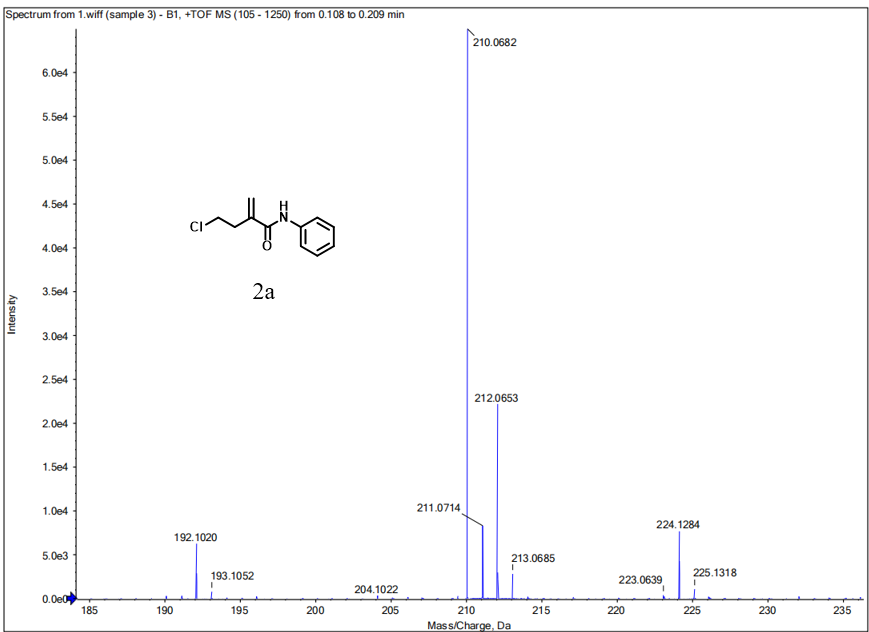


^1^H NMR (400 MHz, CDCl_3_) of **2b**


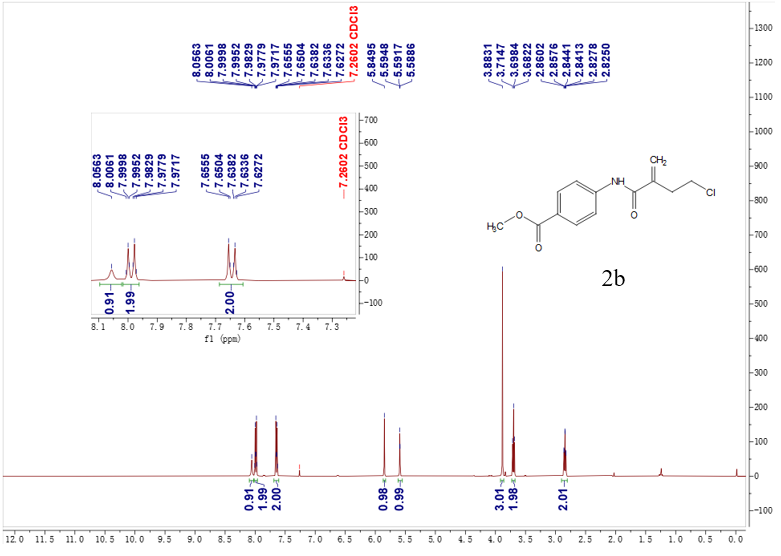


^13^C NMR (101 MHz, CDCl_3_) of **2b**


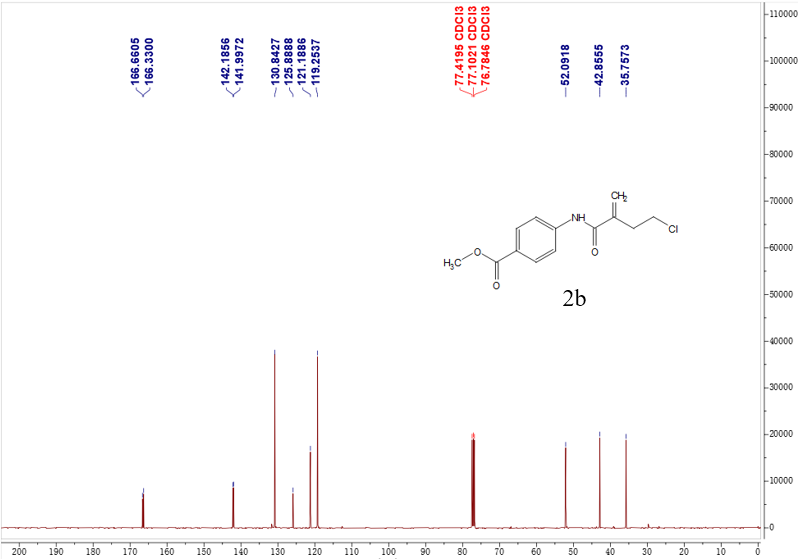


HRMS of **2b** (calcd for C_13_H_14_ClNO_3_ [M+H] ^+^ 268.0735)


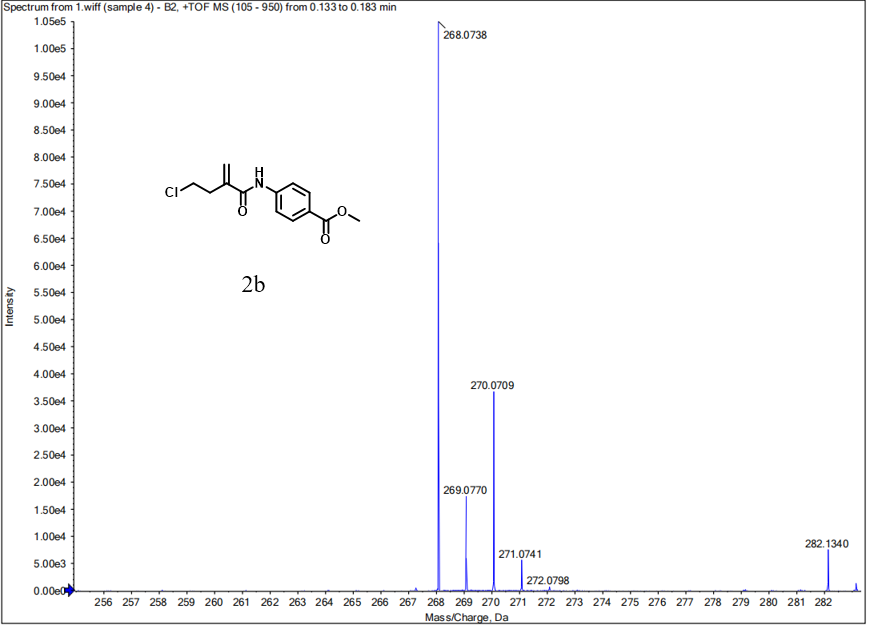


^1^H NMR (400 MHz, CDCl_3_) of **2c**


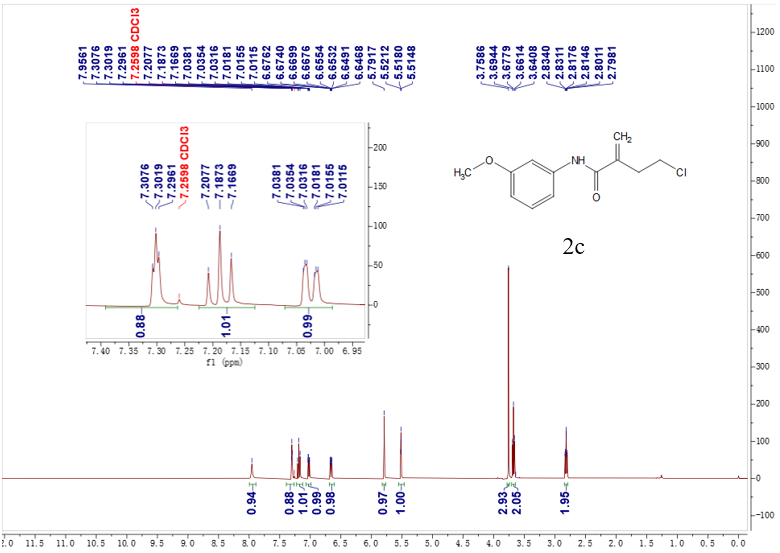


^13^C NMR (101 MHz, CDCl_3_) of **2c**


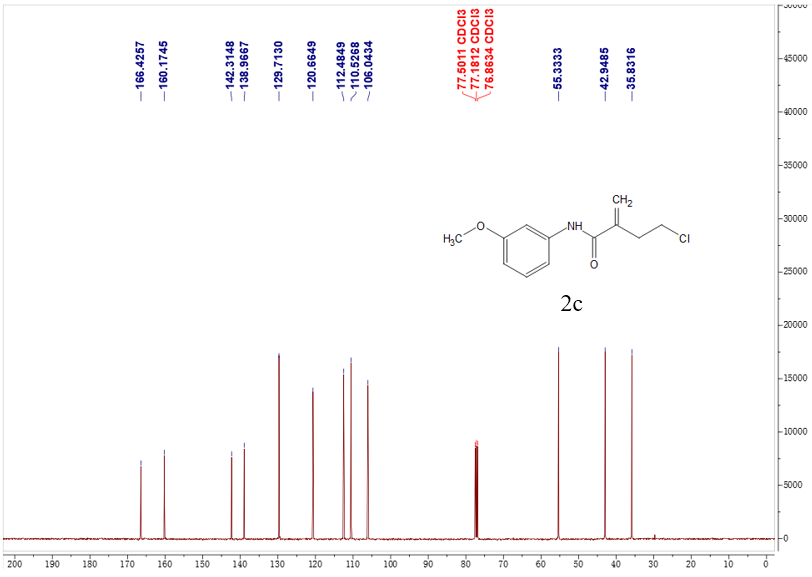


HRMS of **2c** (calcd for C_12_H_14_ClNO_2_ [M+H] ^+^ 240.0785)


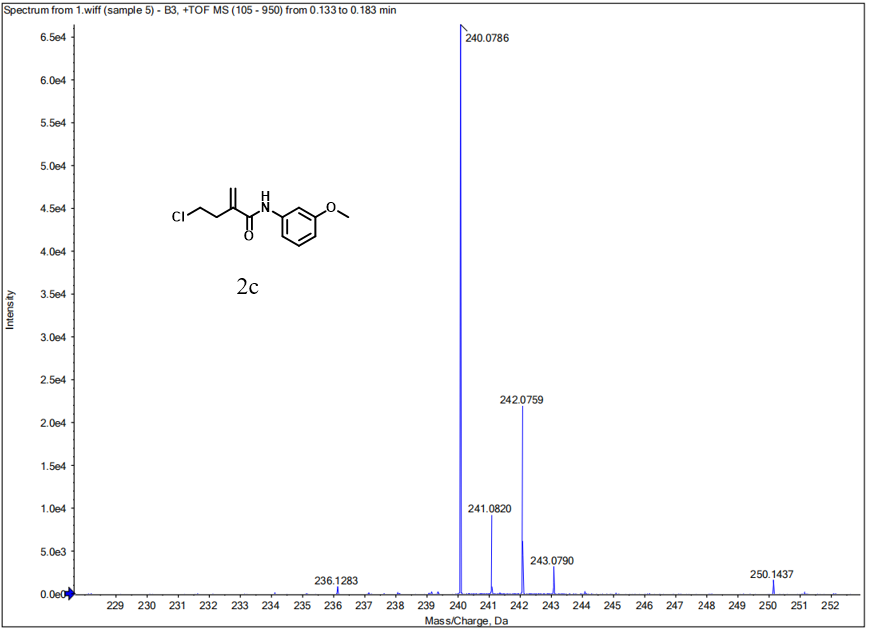


^1^H NMR (500 MHz, CDCl_3_) of **2d**

^
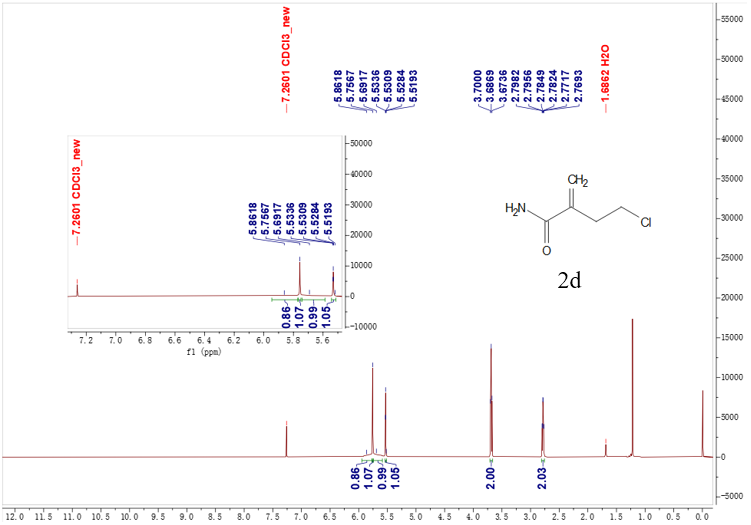
^

^1^H NMR (500 MHz, CDCl_3_) of **2e**


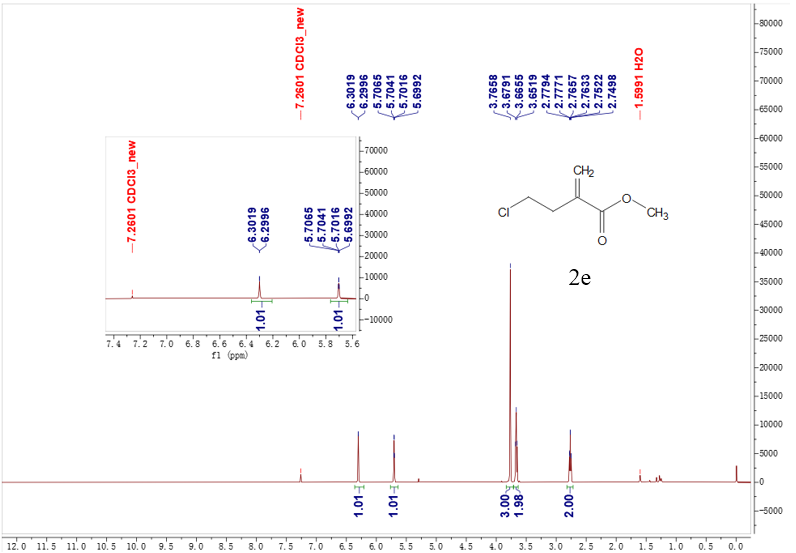


^1^H NMR (400 MHz, CDCl_3_) of **2f**


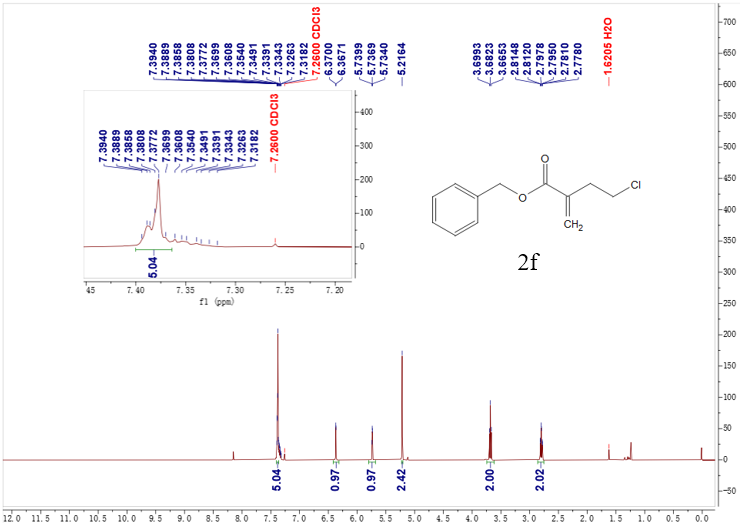


^1^H NMR (500 MHz, CDCl_3_) of **2g**


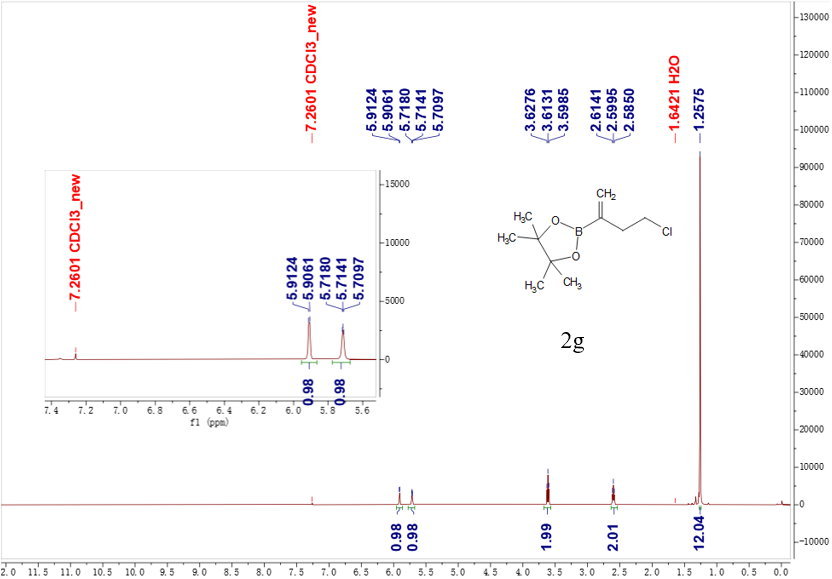


^1^H NMR (400 MHz, CDCl_3_) of **2h**


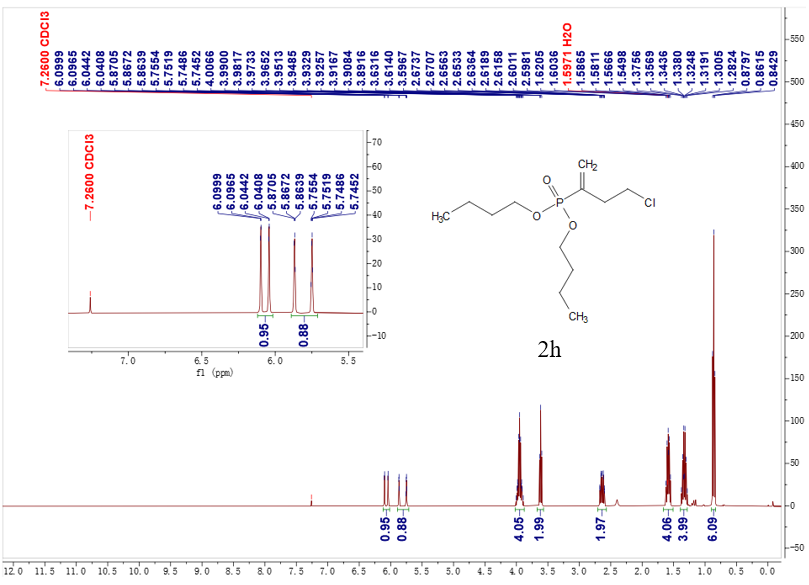


^1^H NMR (400 MHz, CDCl_3_) of **2i**


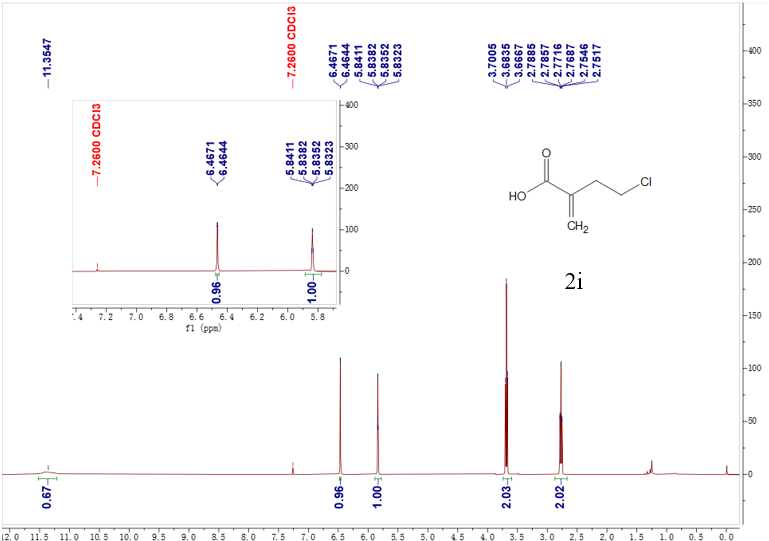


^1^H NMR (400 MHz, CDCl_3_) of **2j**


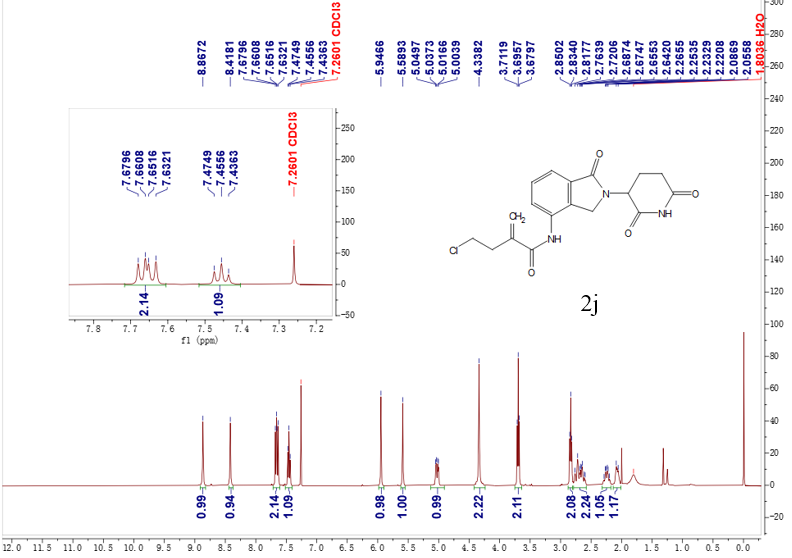


^13^C NMR (101 MHz, CDCl_3_) of **2j**


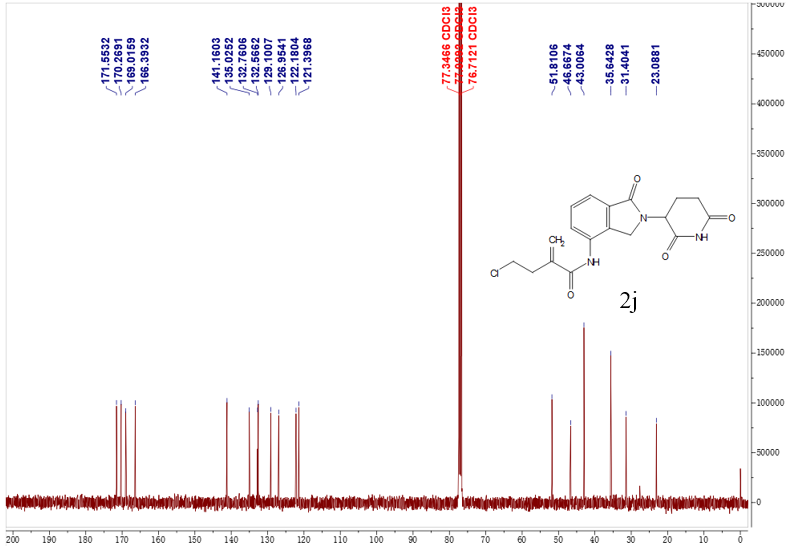


HRMS of **2j** (calcd for C_18_H_18_ClN_3_O_4_ [M+H] ^+^ 376.1058)


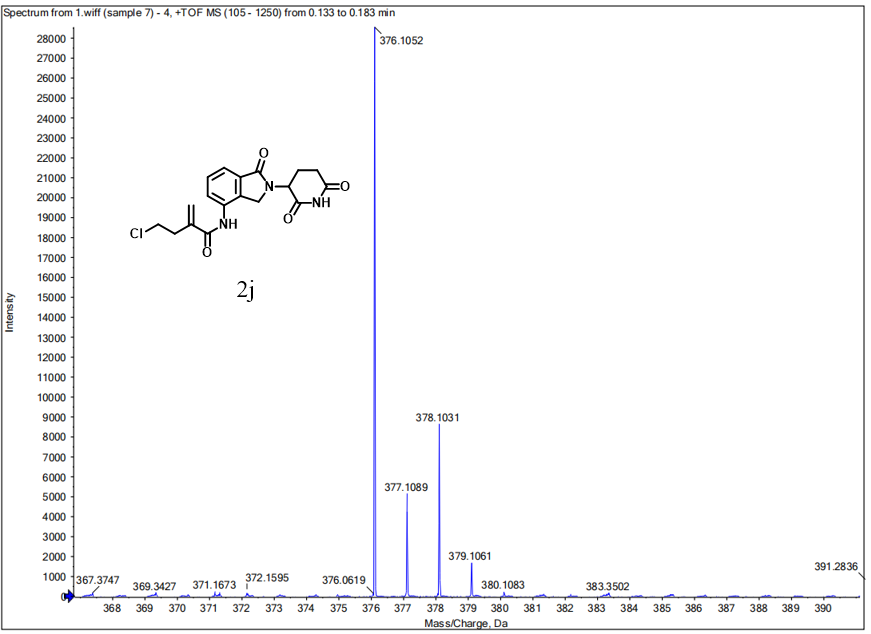


^1^H NMR (400 MHz, CDCl_3_) of **2k**


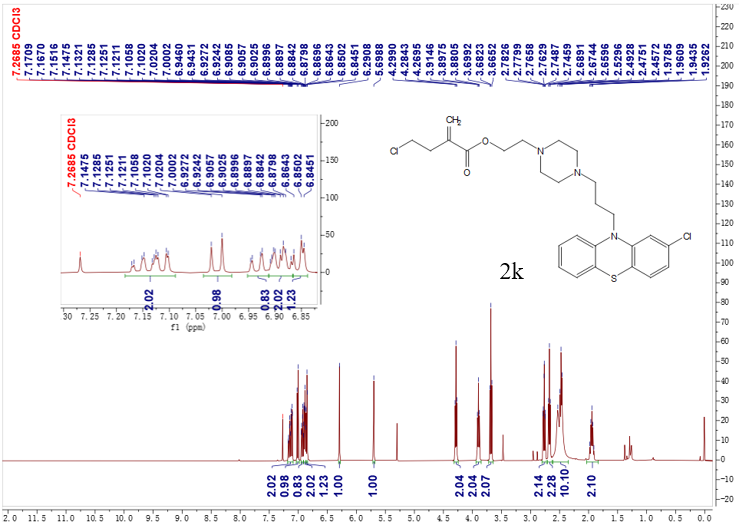


^13^C NMR (101 MHz, CDCl_3_) of **2k**


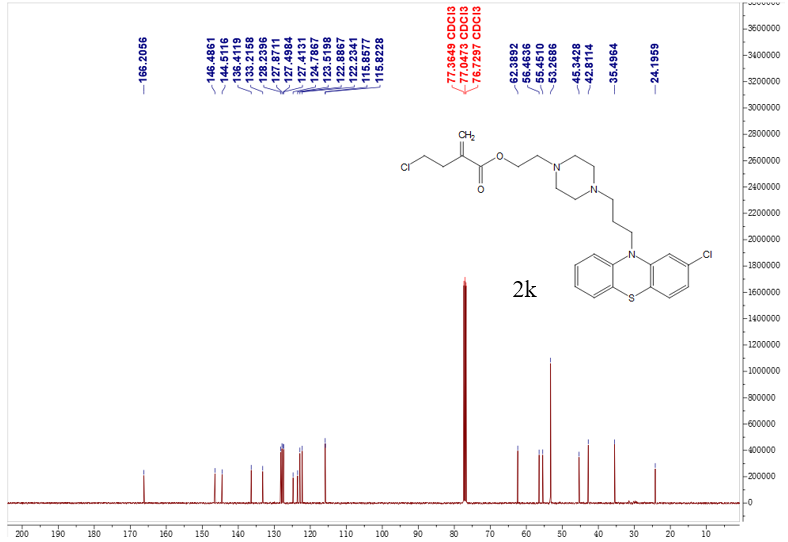


HRMS of **2k** (calcd for C_26_H_31_Cl_2_N_3_O_2_S [M+H] ^+^ 520.1586)


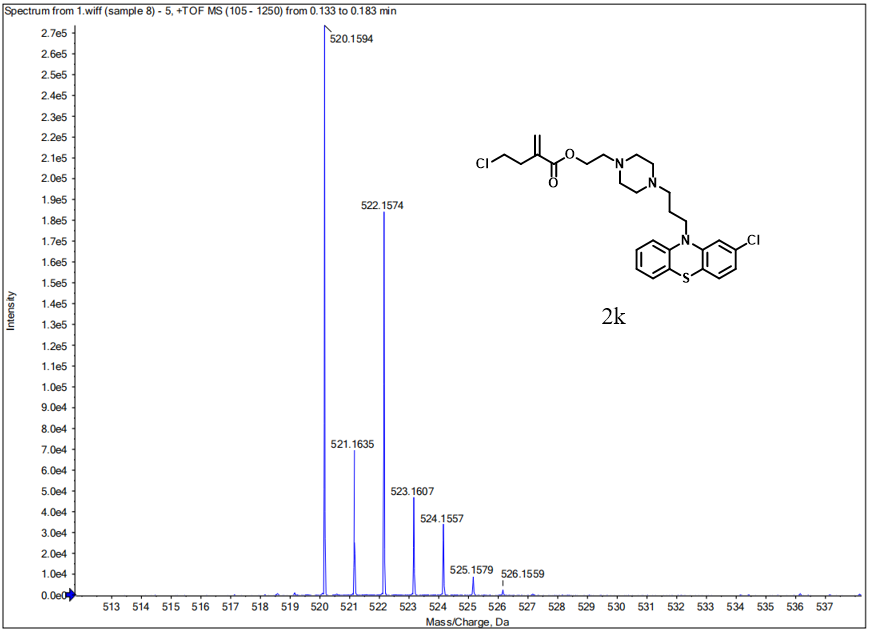


^1^H NMR (400 MHz, CDCl_3_) of **2l**


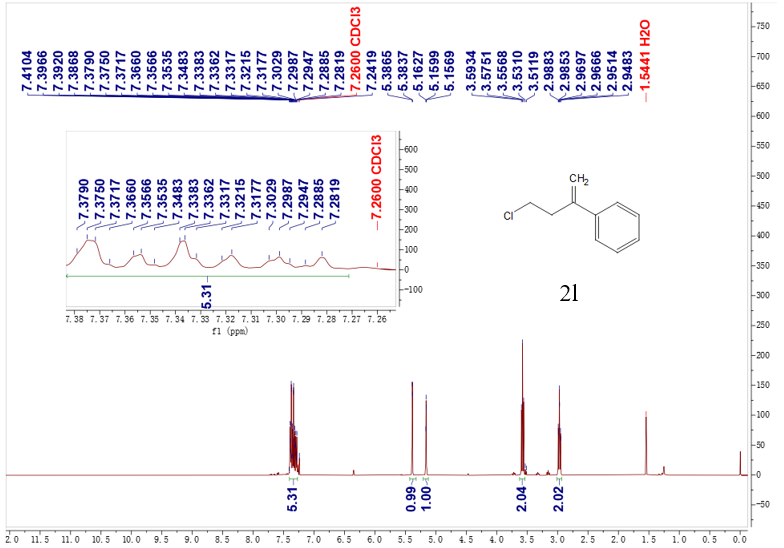


^1^H NMR (400 MHz, CDCl_3_) of **2m**


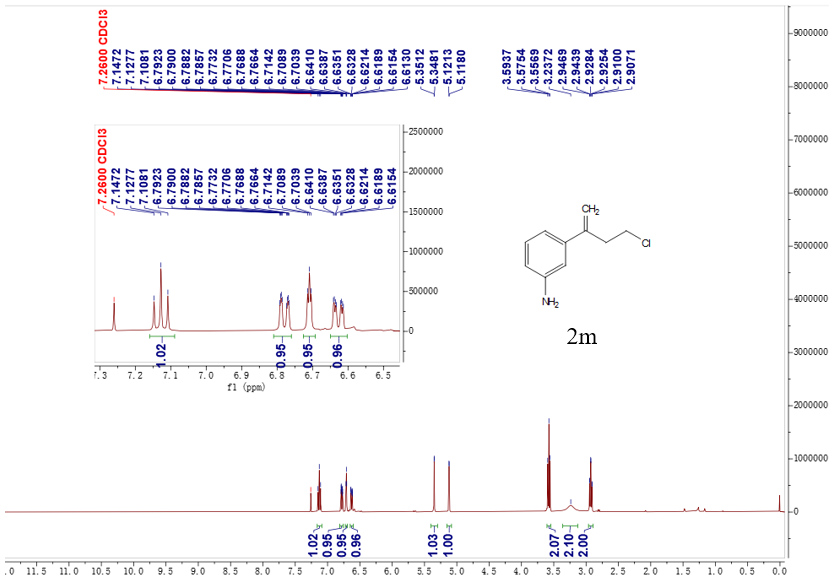


^13^C NMR (101 MHz, CDCl_3_) of **2m**


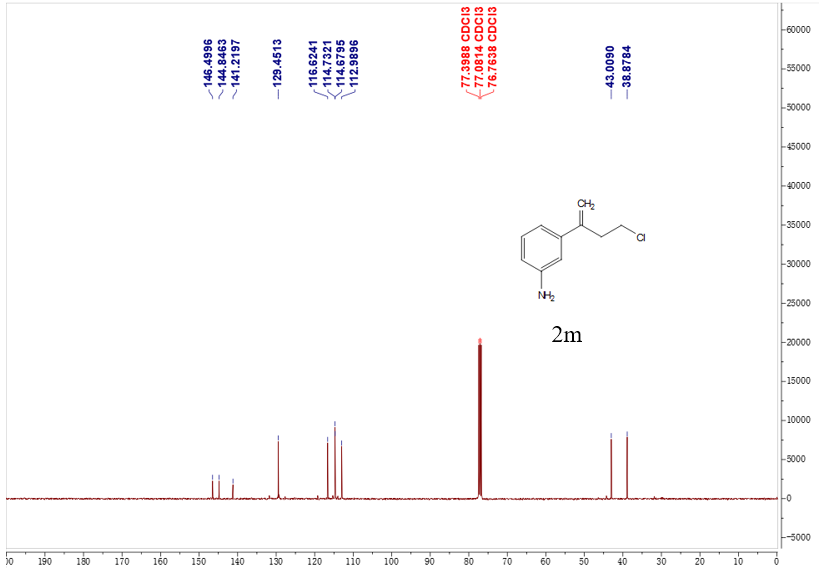


HRMS of **2m** (calcd for C_26_H_31_Cl_2_N_3_O_2_S [M+H] ^+^ 182.0731)


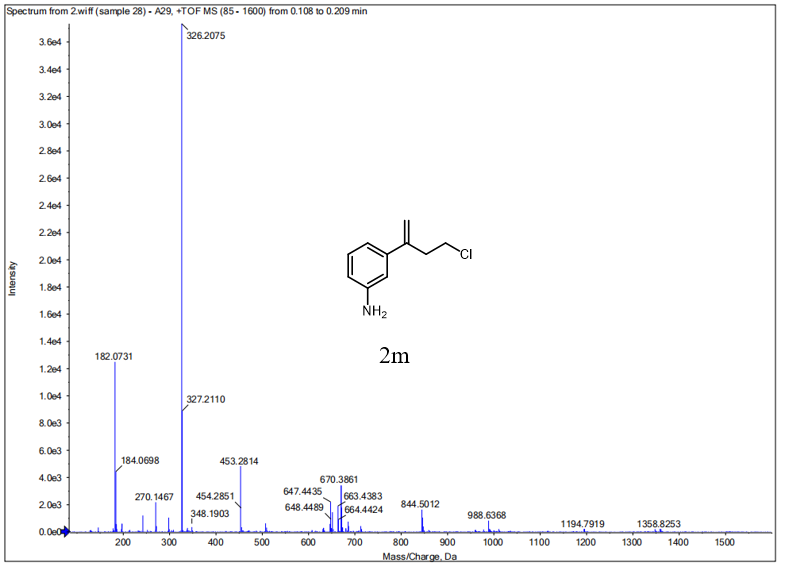


^1^H NMR (400 MHz, CDCl_3_) of **2n**


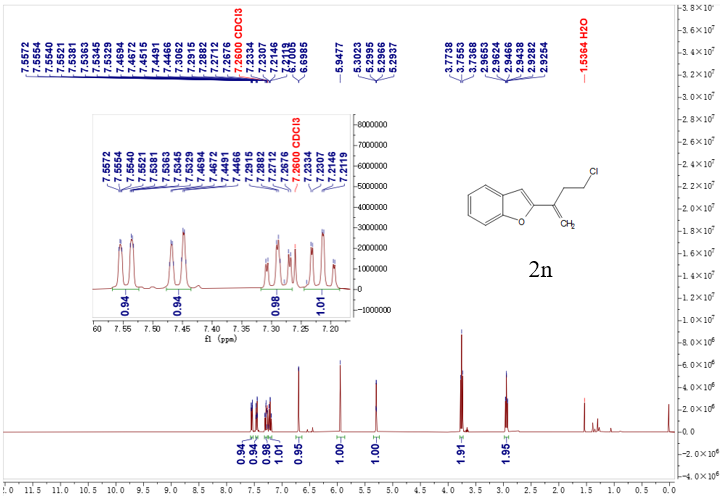


^1^H NMR (400 MHz, CDCl_3_) of **4a**

**
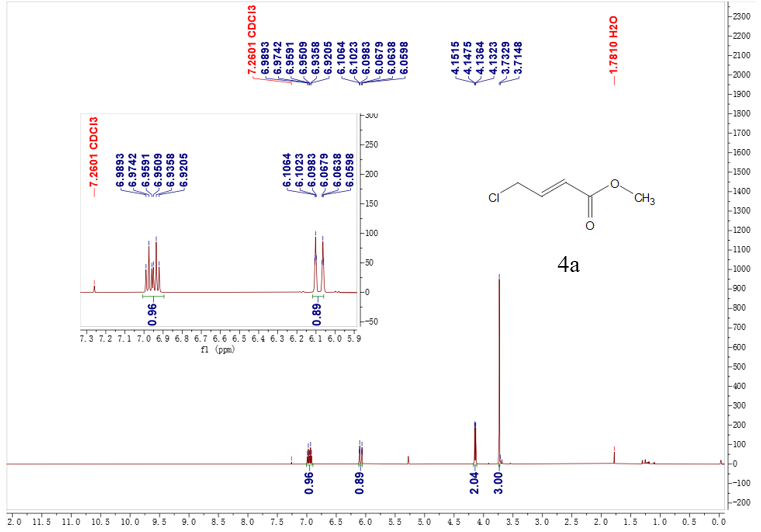
**

^1^H NMR (400 MHz, CDCl_3_) of **4b**


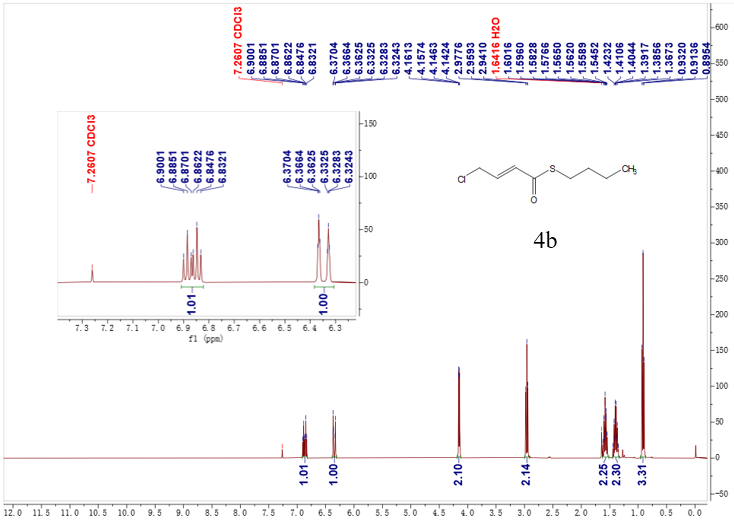


^13^C NMR (101 MHz, CDCl_3_) of **4b**


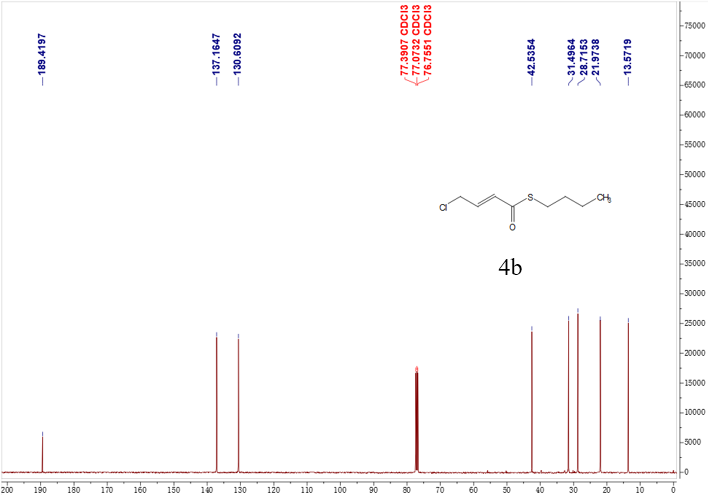


HRMS of **4b** (calcd for C_8_H_13_ClOS [M+H] ^+^ 193.0448)


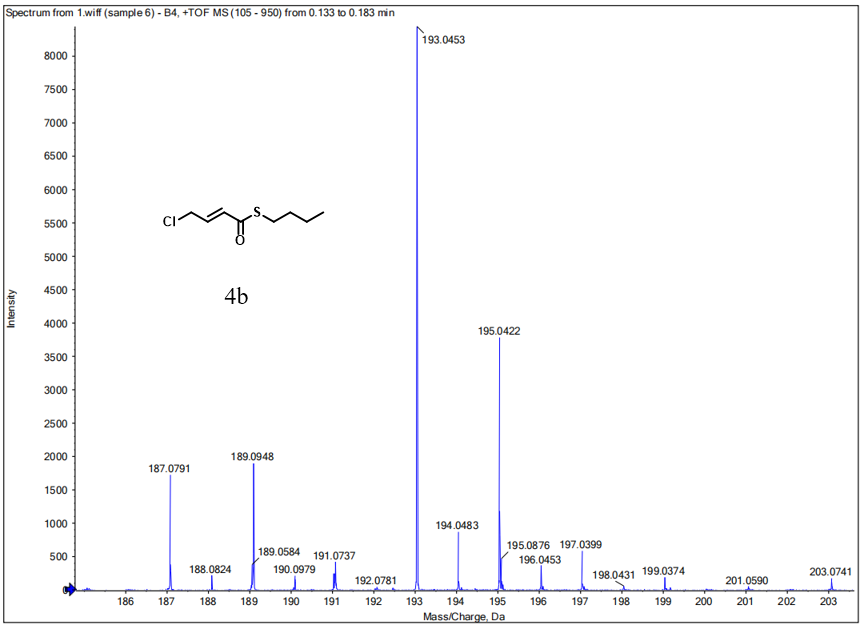


^1^H NMR (400 MHz, CDCl_3_) of **3a**


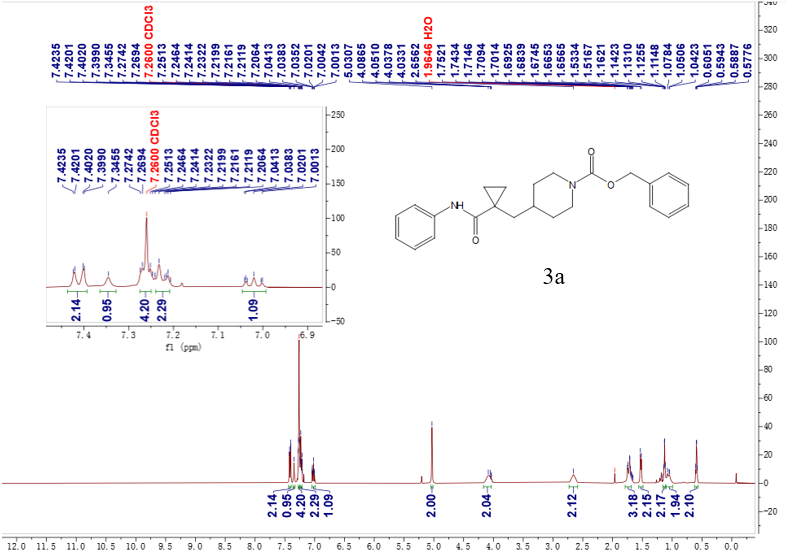


^13^C NMR (101 MHz, CDCl_3_) of **3a**


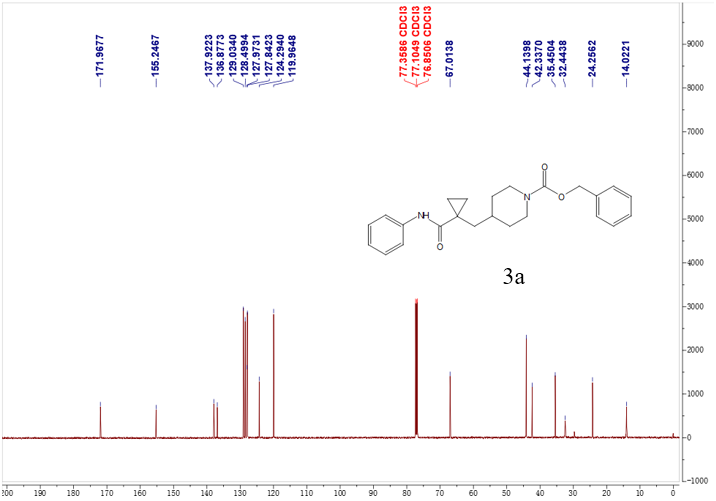


HRMS of **3a** (calcd. for C_24_H_28_N_2_O_3_ [M+H] ^+^ 393.2172)


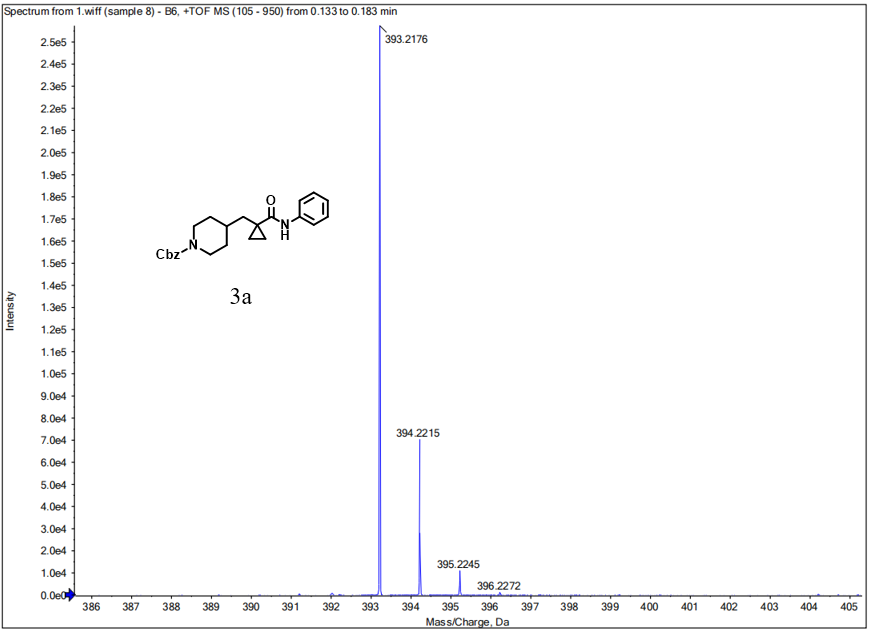


^1^H NMR (400 MHz, CDCl_3_) of **3b**


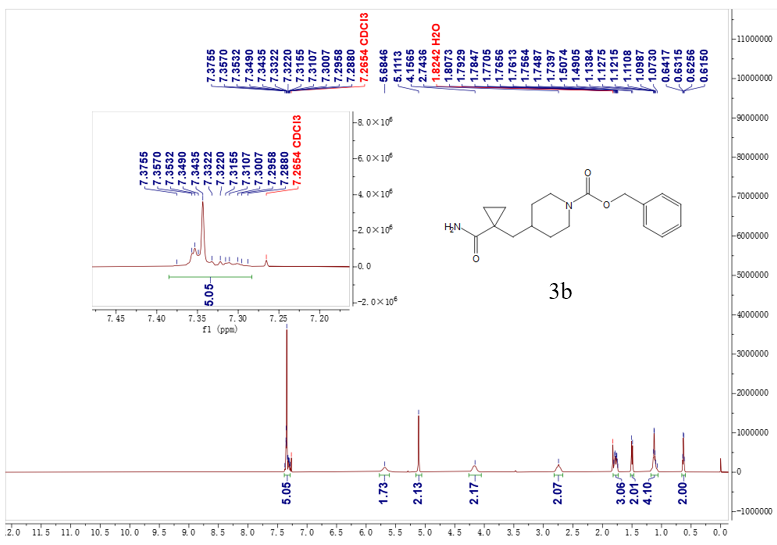


^13^C NMR (101 MHz, CDCl_3_) of **3b**


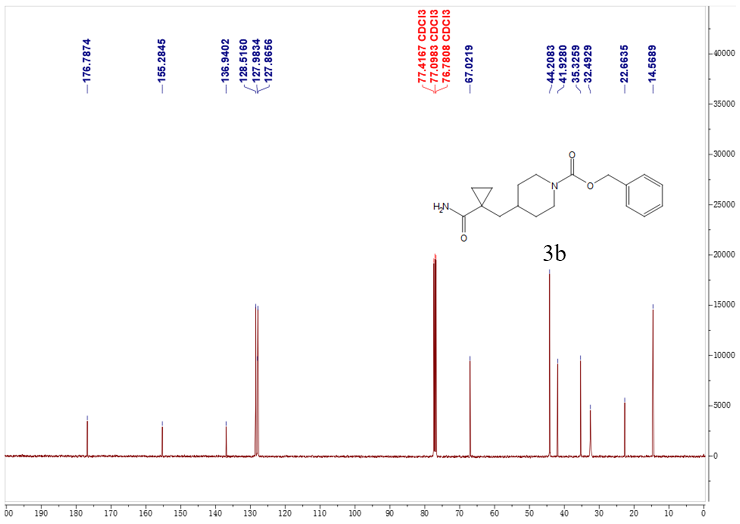


HRMS of **3b** (calcd for C_18_H_24_N_2_O_3_ [M+H] ^+^ 317.1860)


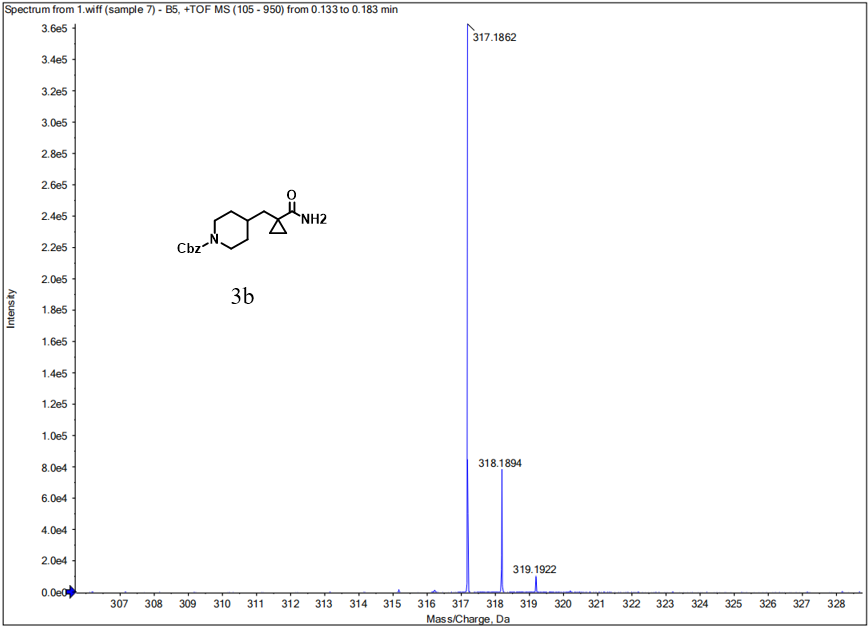


^1^H NMR (400 MHz, CDCl_3_) of **3c**


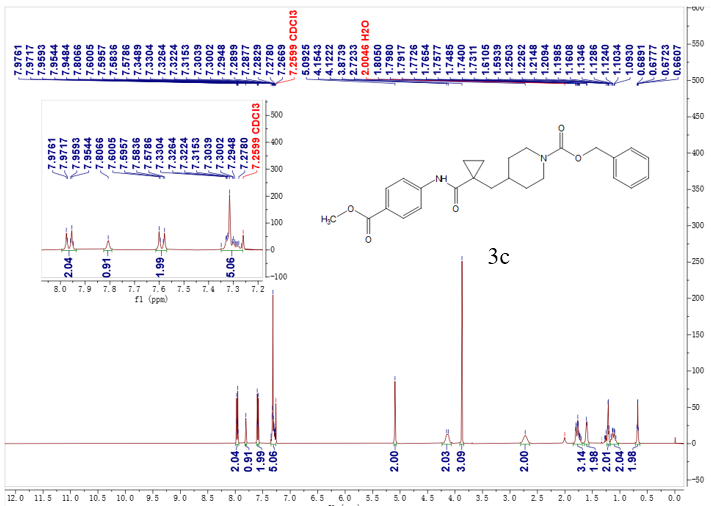


^13^C NMR (101 MHz, CDCl_3_) of **3c**


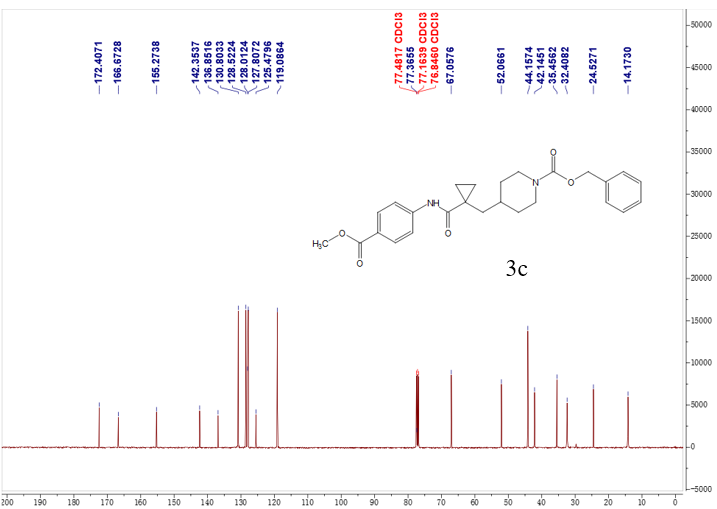


HRMS of **3c** (calcd for C_26_H_30_N_2_O_5_ [M+H] ^+^ 451.2228)


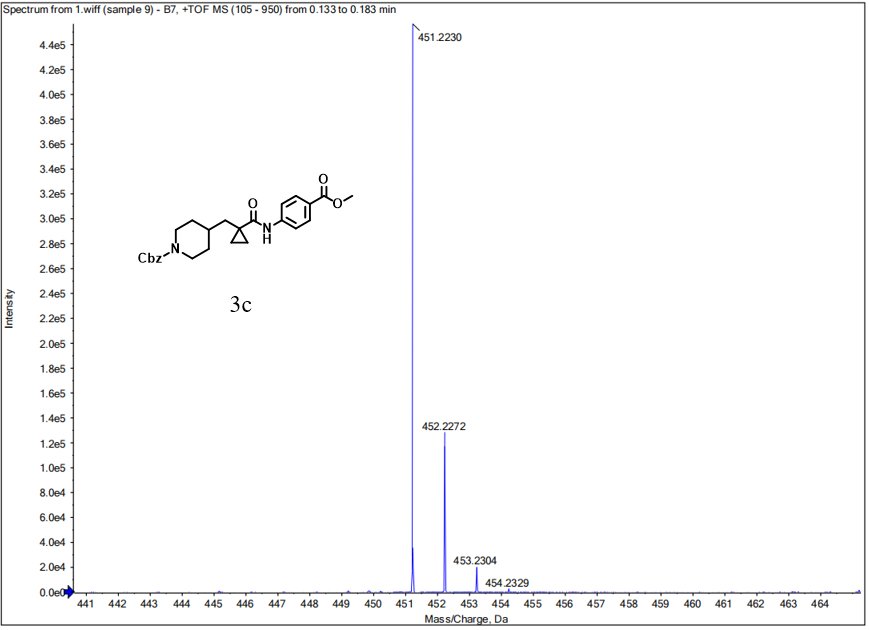


^1^H NMR (400 MHz, CDCl_3_) of **3d**


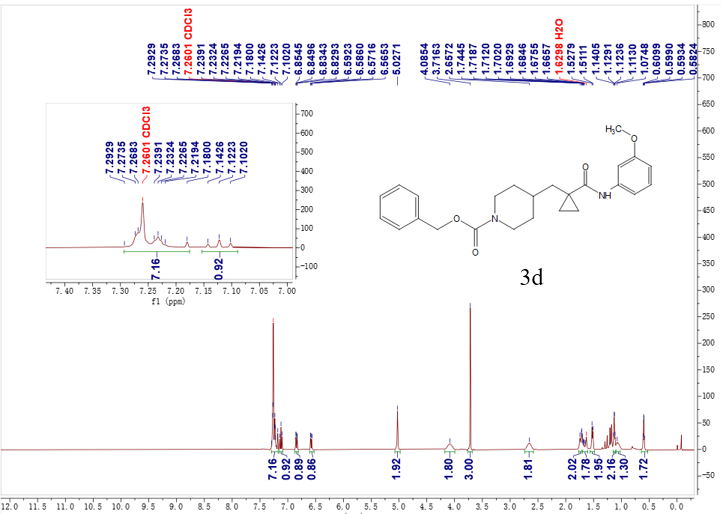


^13^C NMR (101 MHz, CDCl_3_) of **3d**


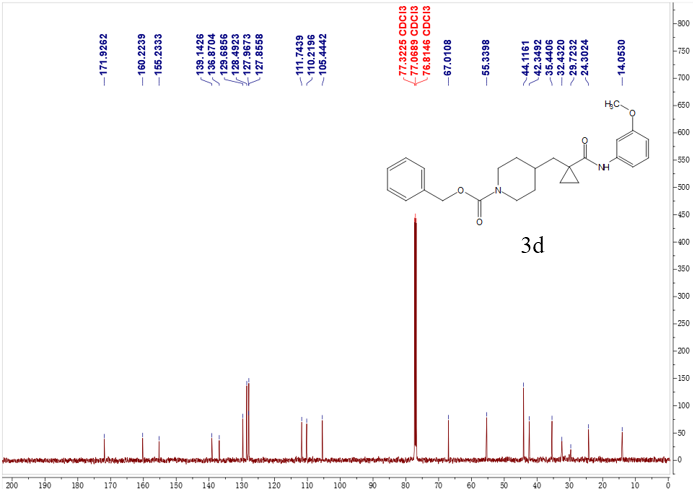


HRMS of **3d** (calcd for C_25_H_30_N_2_O_4_ [M+H] ^+^ 423.2278)


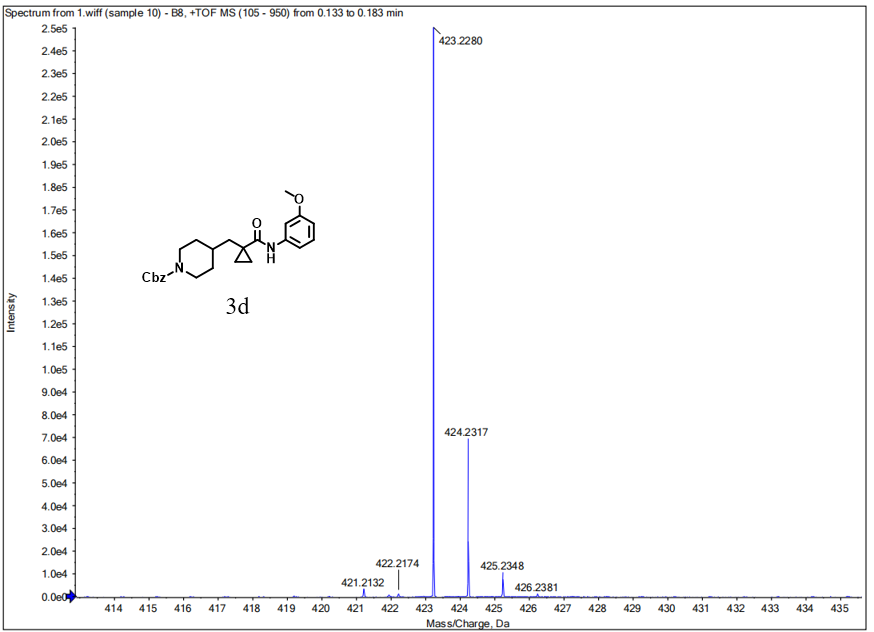


^1^H NMR (400 MHz, CDCl_3_) of **3e**


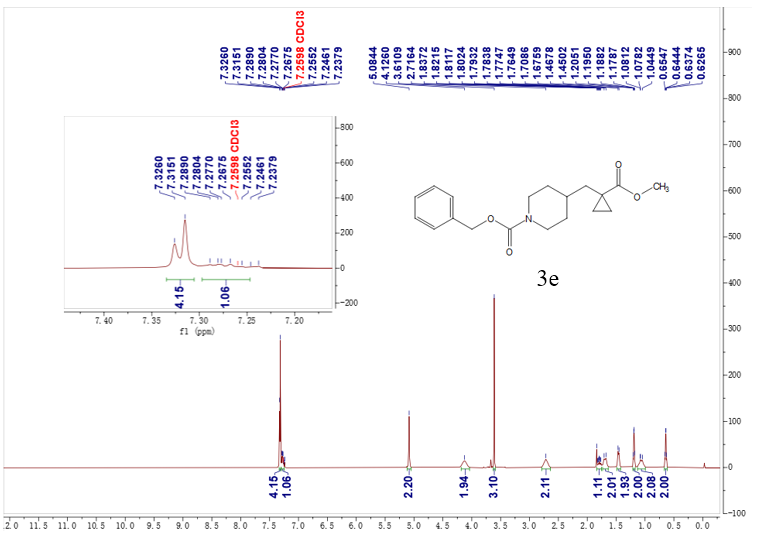


^13^C NMR (101 MHz, CDCl_3_) of **3e**


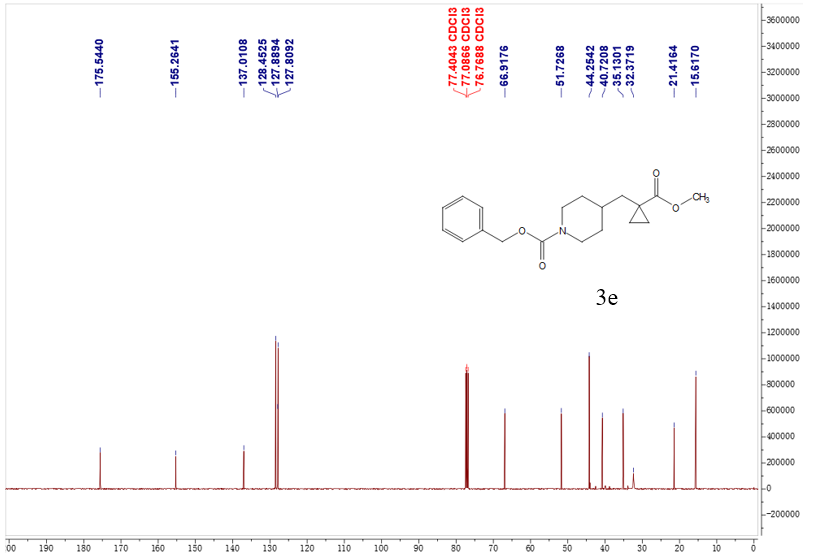


HRMS of **3e** (calcd for C_19_H_25_NO_4_ [M+H] ^+^ 332.1856)


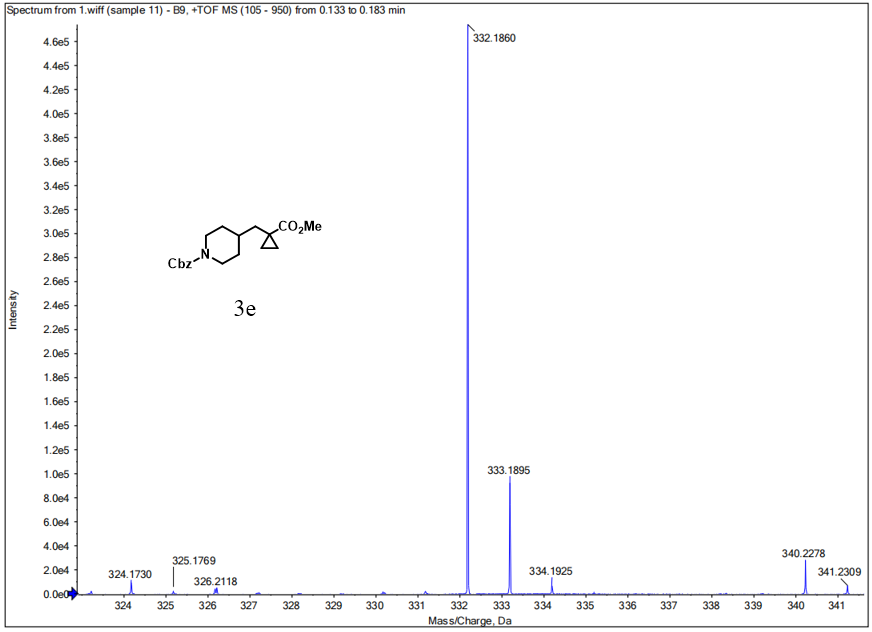


^1^H NMR (400 MHz, CDCl_3_) of **3f**


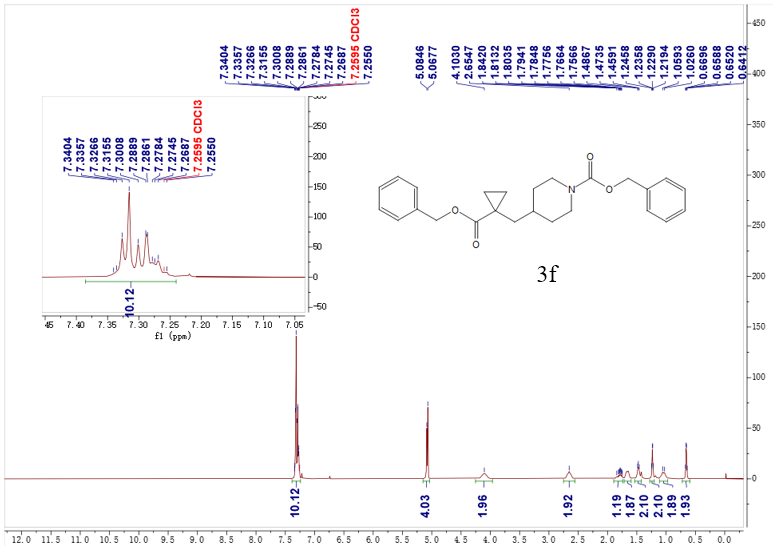


^13^C NMR (101 MHz, CDCl_3_) of **3f**


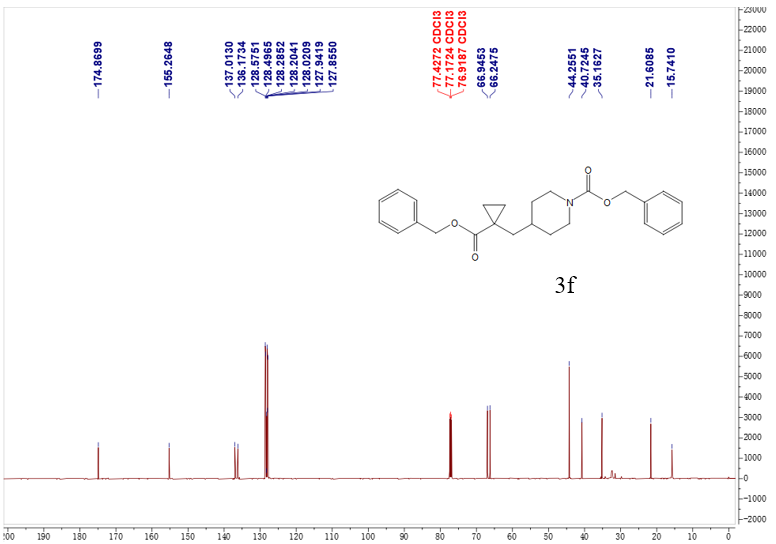


HRMS of **3f** (calcd for C_25_H_29_NO_4_ [M+H] ^+^408.2169)


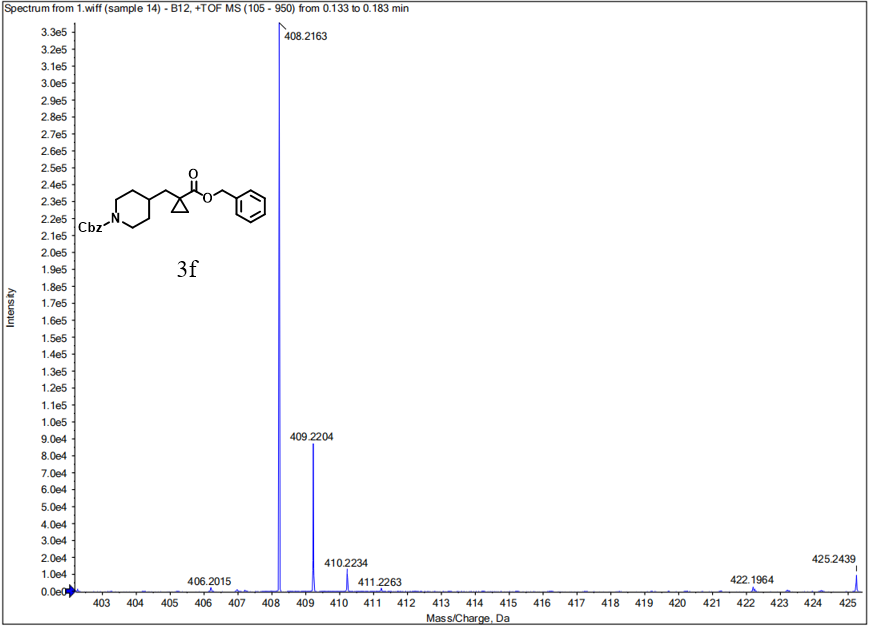


^1^H NMR (400 MHz, CDCl_3_) of **3g**


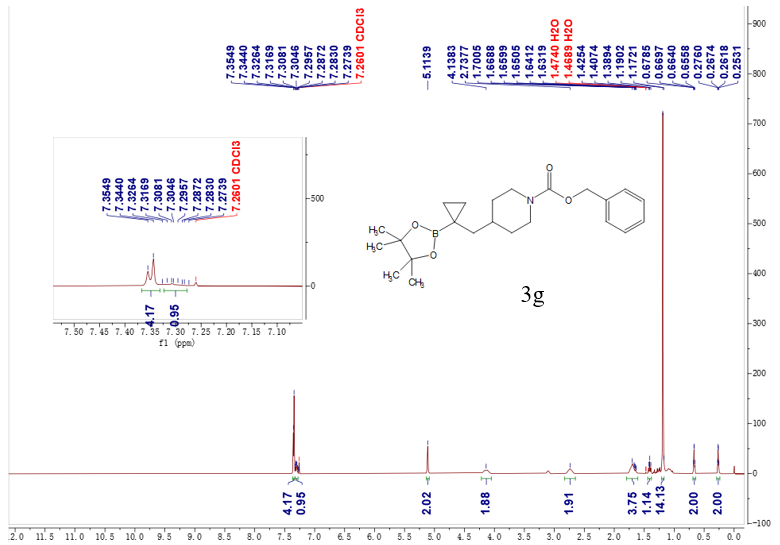


^13^C NMR (101 MHz, CDCl_3_) of **3g**


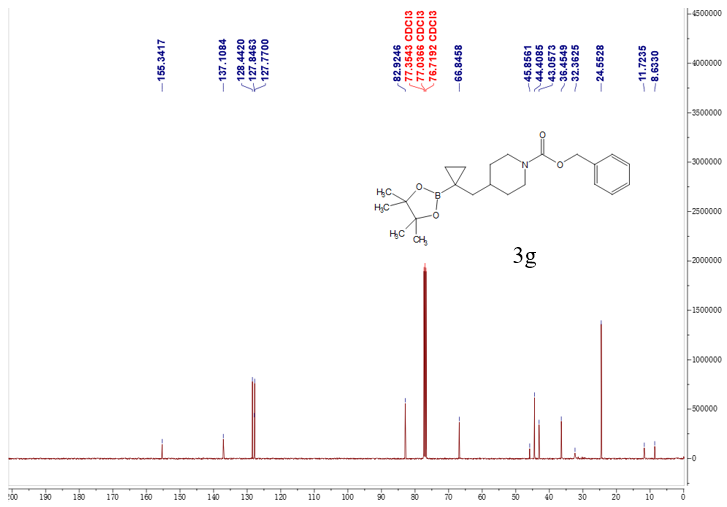


HRMS of **3g** (calcd for C_23_H_34_BNO_4_ [M+H] ^+^ 400.2654)


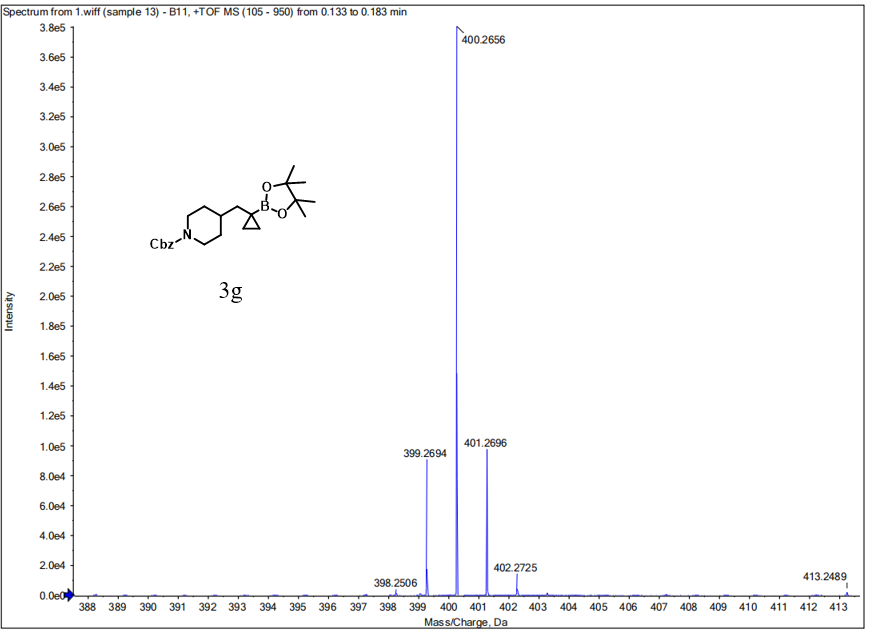


^1^H NMR (400 MHz, CDCl_3_) of **3h**


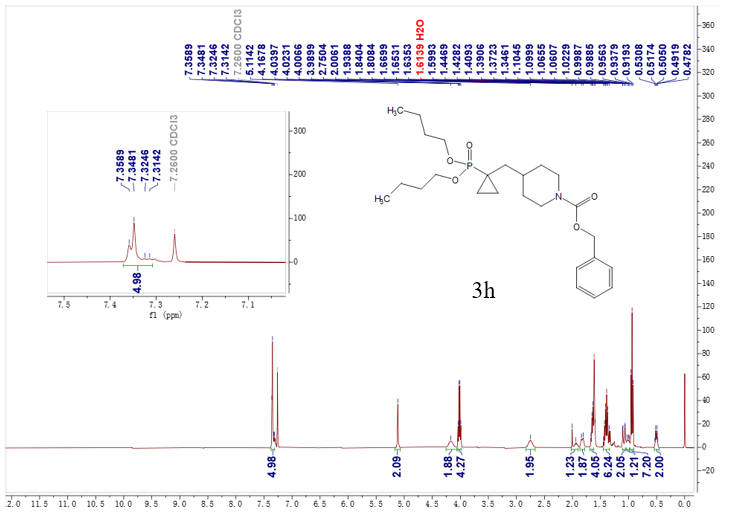


^13^C NMR (101 MHz, CDCl_3_) of **3h**


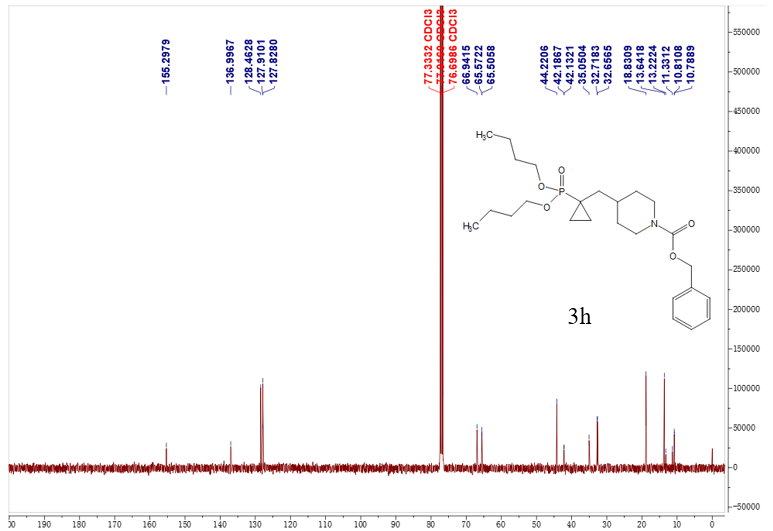


^31^P NMR (162 MHz, DMSO-d6) of **3h**


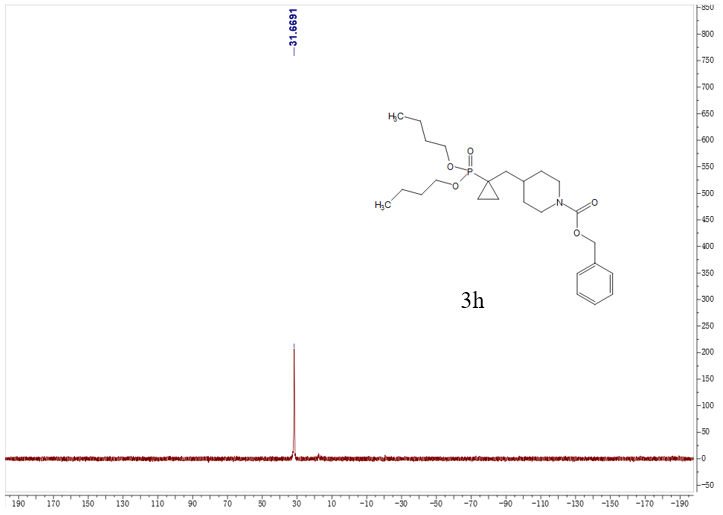


HRMS of **3h** (calcd for C_25_H_40_NO_5_P [M+H] ^+^ 466.2717)


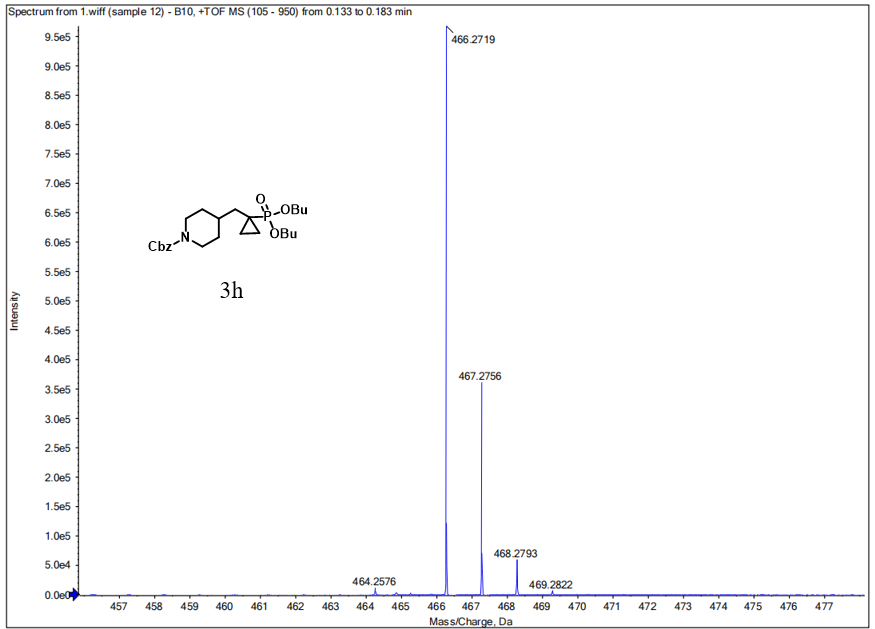


^1^H NMR (400 MHz, CDCl_3_) of **3i**


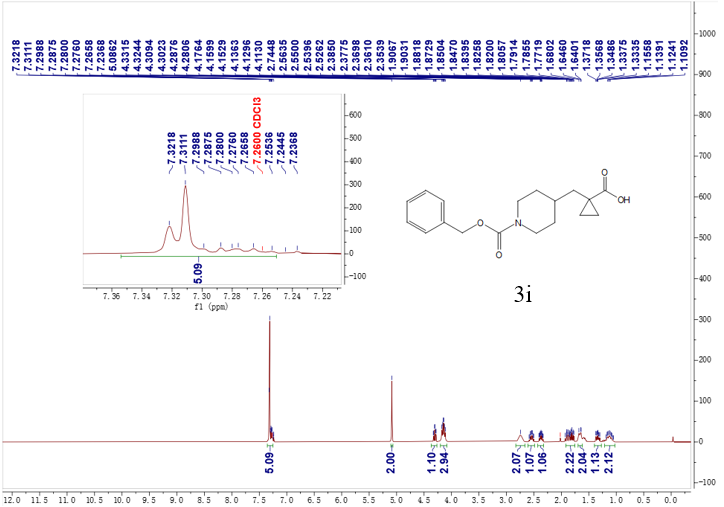


^13^C NMR (101 MHz, CDCl_3_) of **3i**


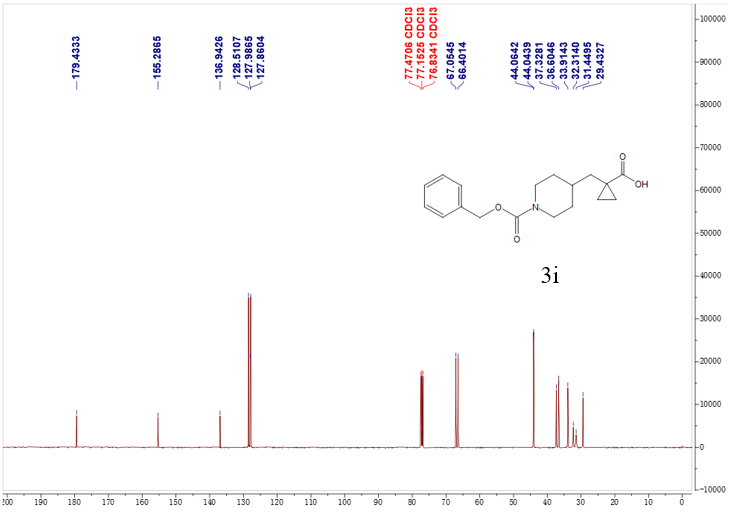


HRMS of **3i** (calcd for C_18_H_23_NO_4_ [M+H] ^+^ 318.1700)
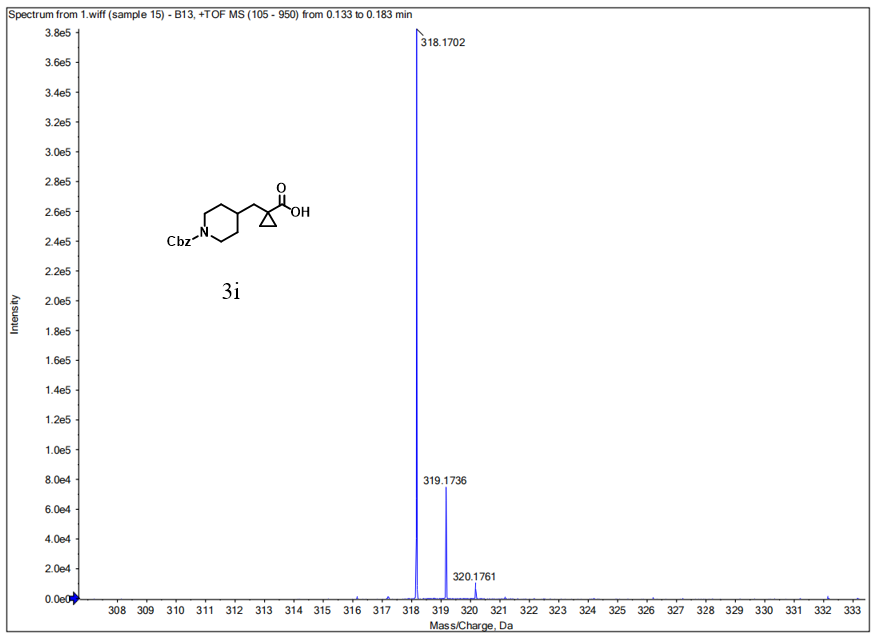


^1^H NMR (400 MHz, CDCl_3_) of **3j**


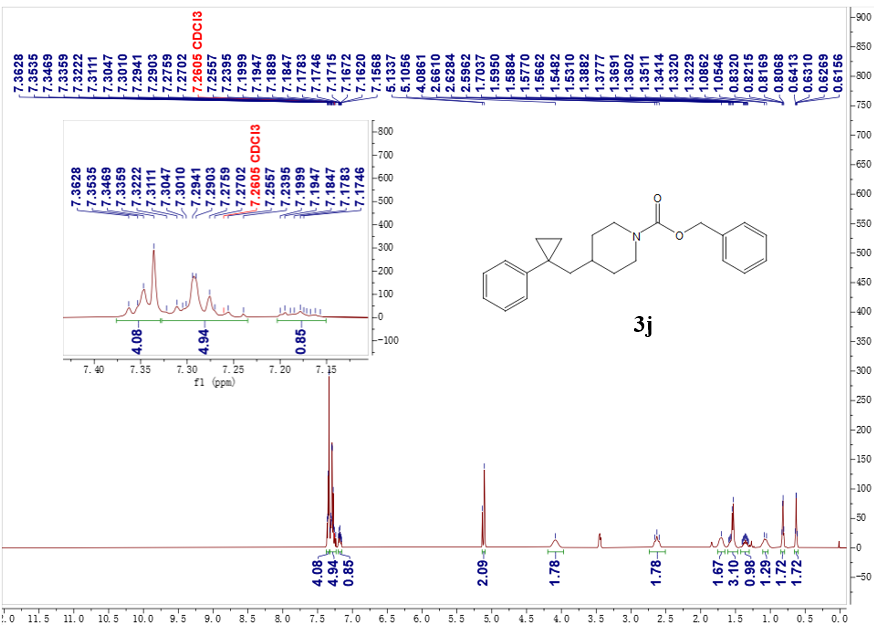


^13^C NMR (101 MHz, CDCl_3_) of **3j**


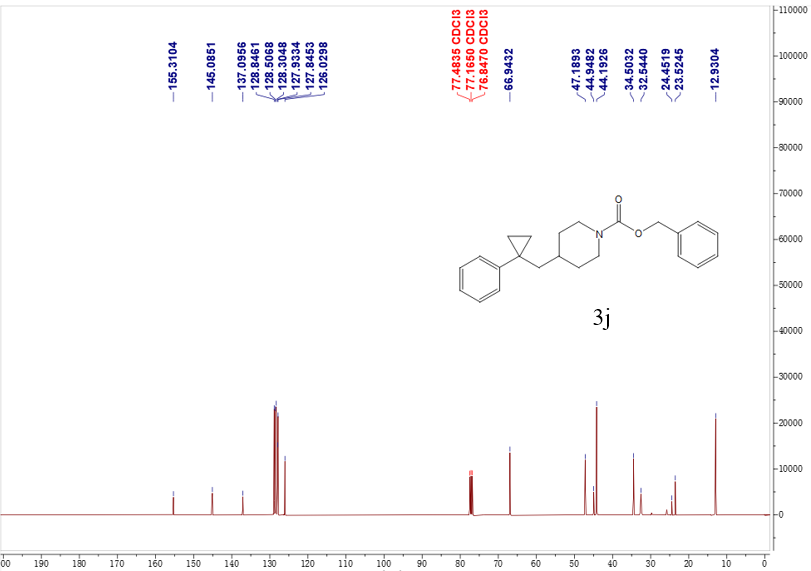


HRMS of **3j** (calcd for C_23_H_27_NO_2_ [M+H] ^+^ 350.2115)


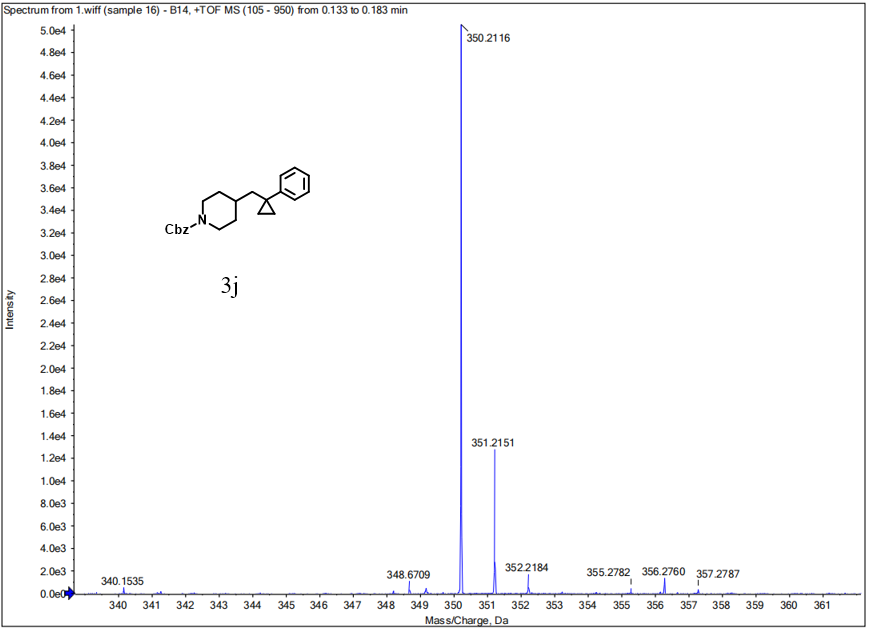


^1^H NMR (400 MHz, CDCl_3_) of **3k**


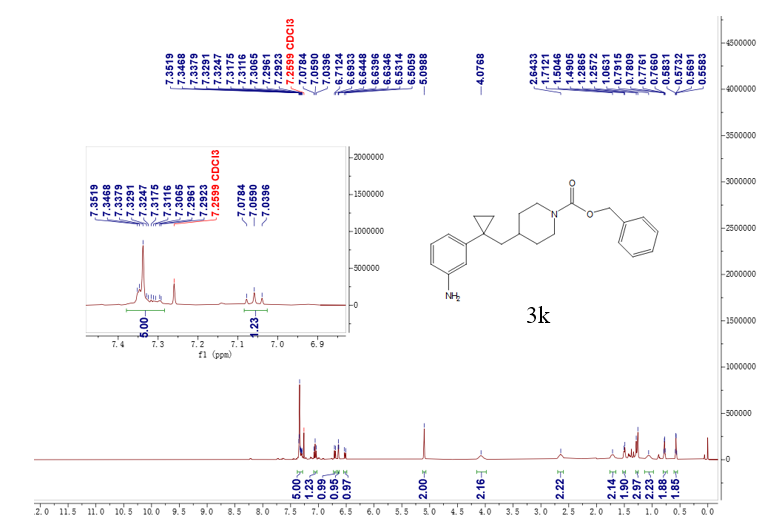


^13^C NMR (101 MHz, CDCl_3_) of **3k**


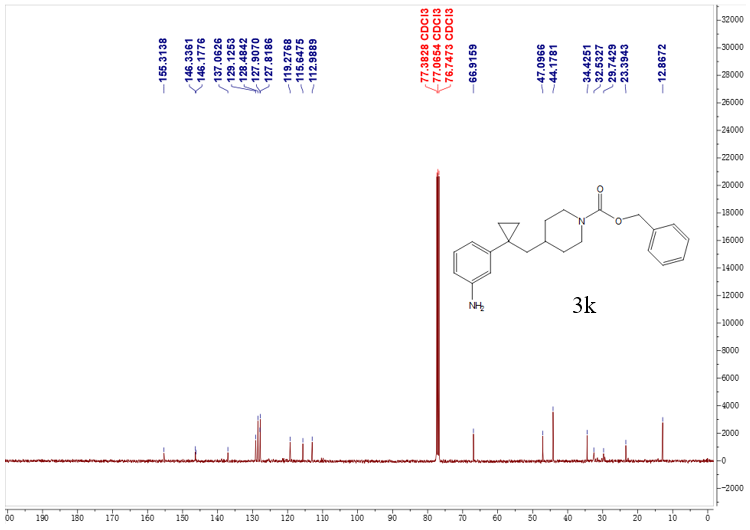


HRMS of 3k (calcd. for C_23_H_27_NO_2_ [M+H] ^+^ 365.2234)


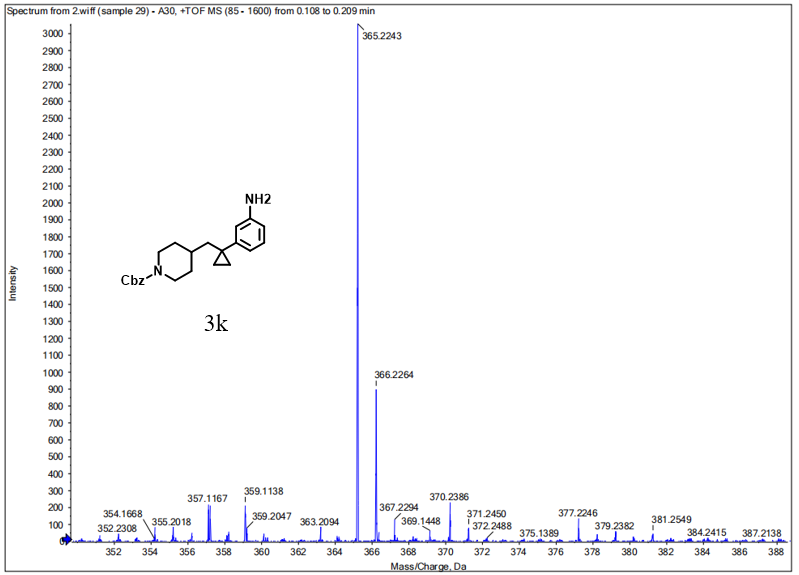


^1^H NMR (400 MHz, CDCl_3_) of **3l**

^
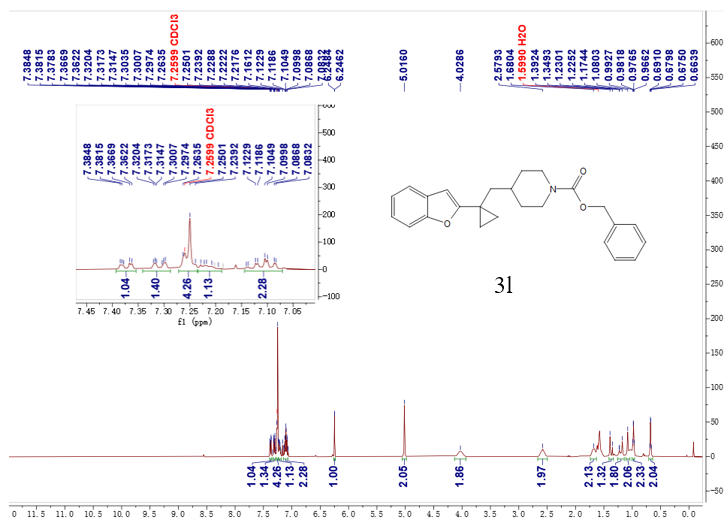
^

^13^C NMR (101 MHz, CDCl_3_) of **3n**

^
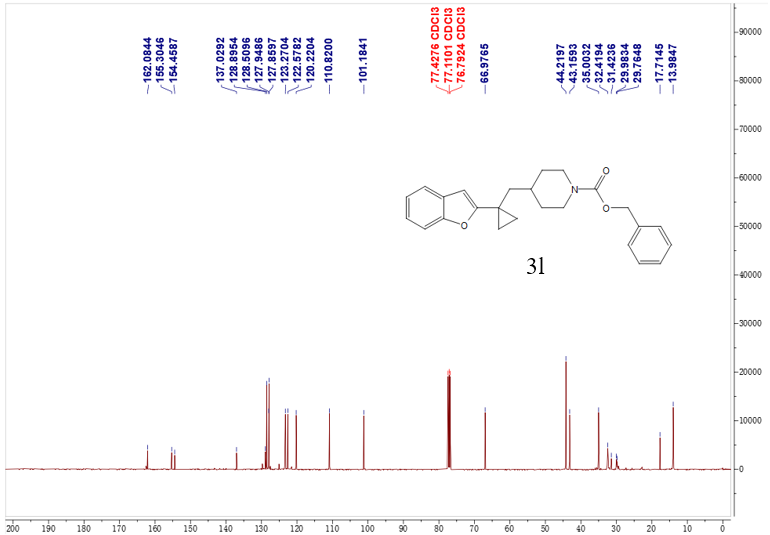
^

HRMS of **3l** (calcd. for C_23_H_27_NO_2_ [M+H] ^+^ 390.2064)


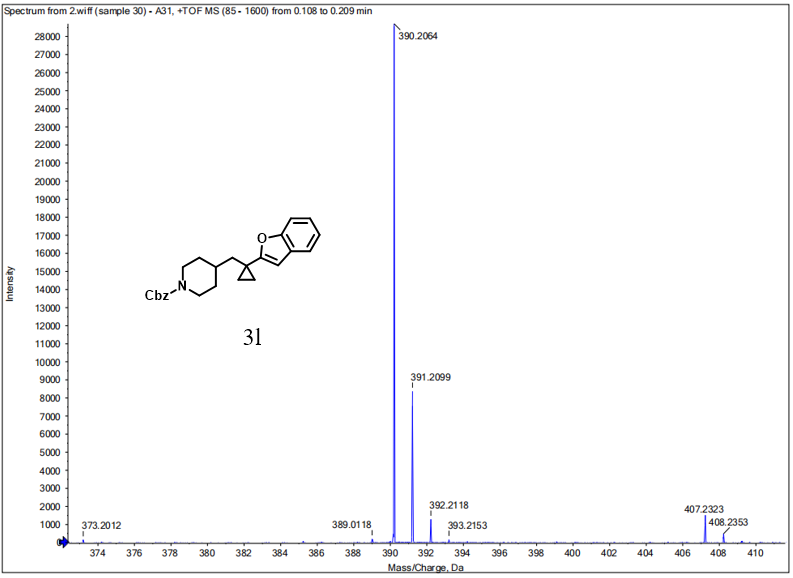


^1^H NMR (400 MHz, CDCl_3_) of **5a**


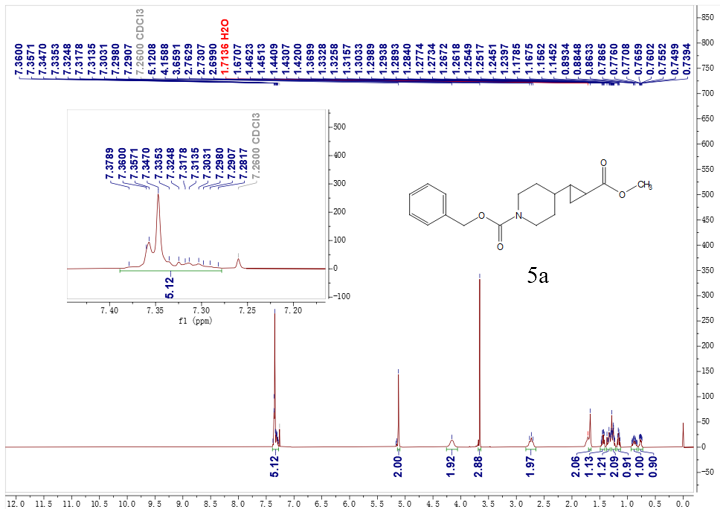


^13^C NMR (101 MHz, CDCl_3_) of **5a**


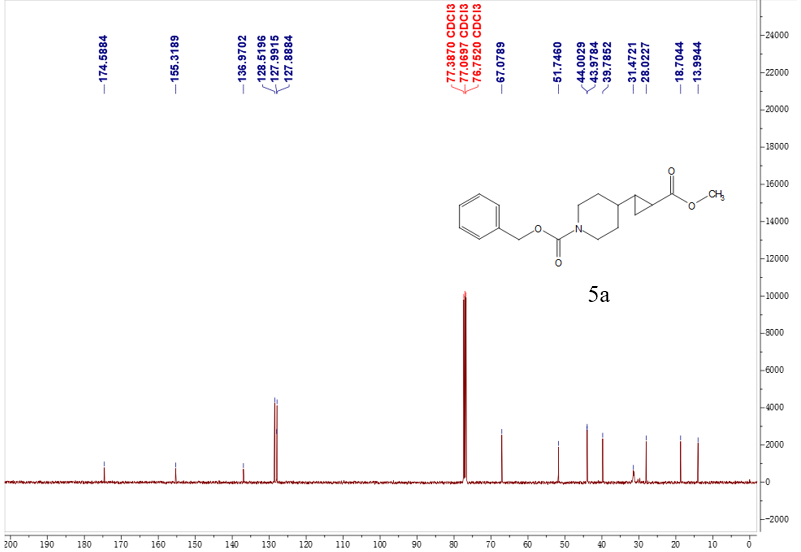


HRMS of **5a** (calcd for C_18_H_23_NO_4_ [M+H] ^+^ 318.1700)


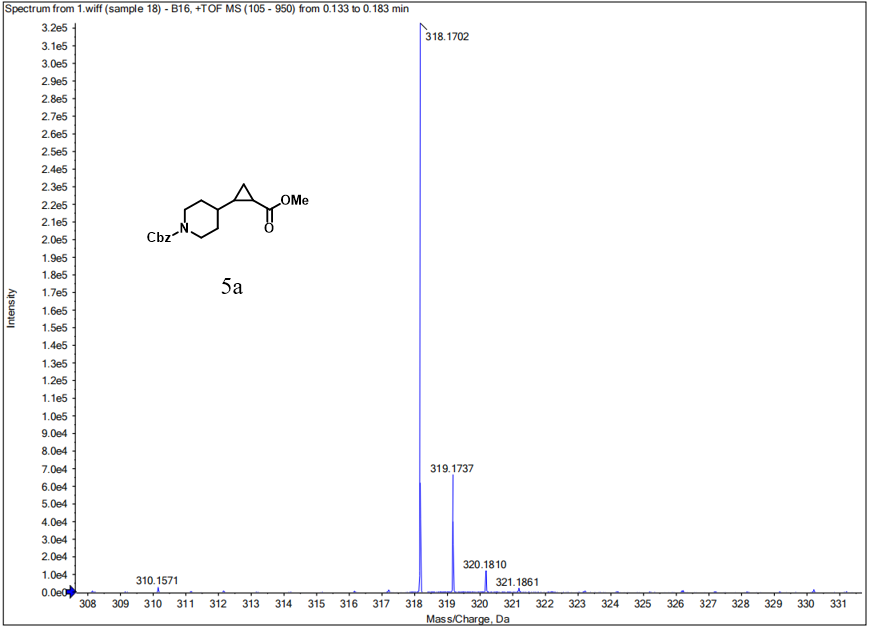


^1^H NMR (400 MHz, CDCl_3_) of **5b**


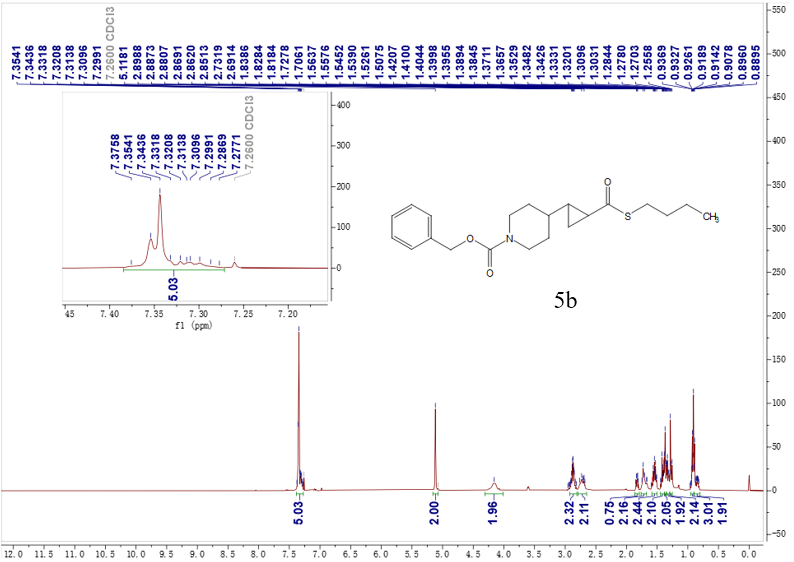


^13^C NMR (101 MHz, CDCl_3_) of **5b**


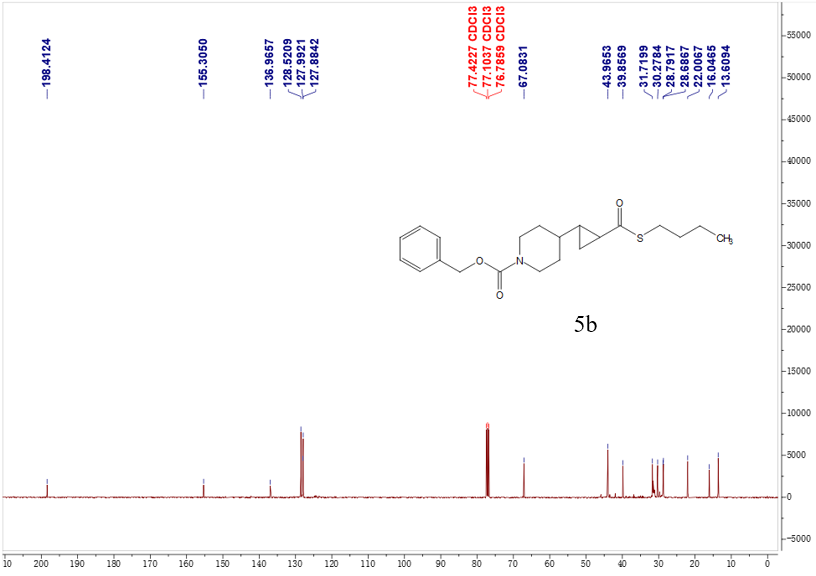


HRMS of **5b** (calcd for C_21_H_29_NO_3_S [M+H] ^+^ 376.1941)


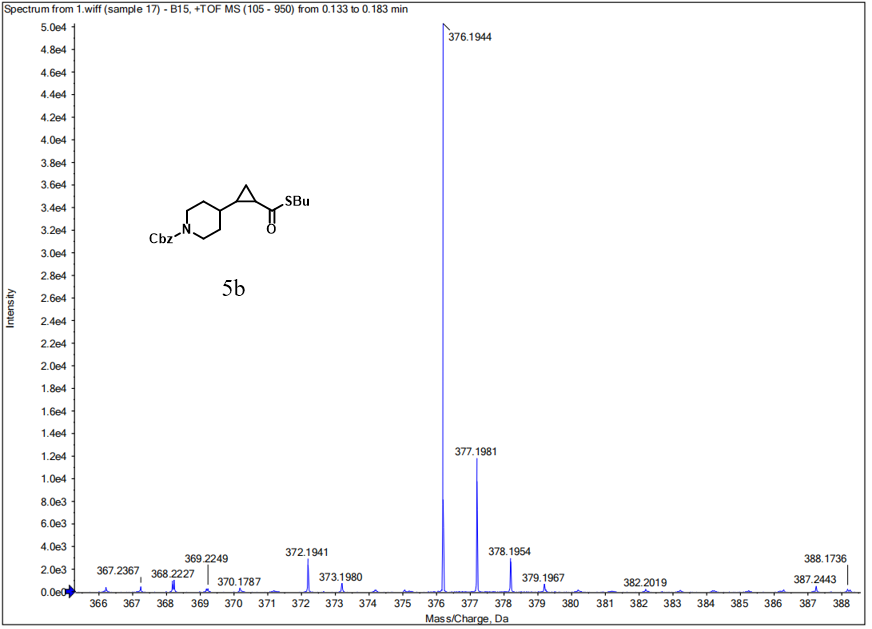


^1^H NMR (400 MHz, CDCl_3_) of **5c**


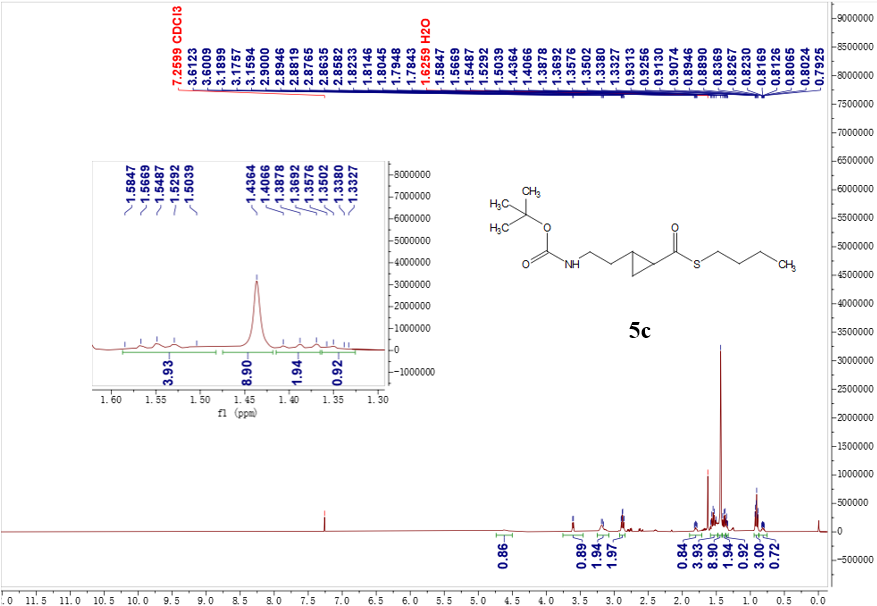


^13^C NMR (101 MHz, CDCl_3_) of **5c**


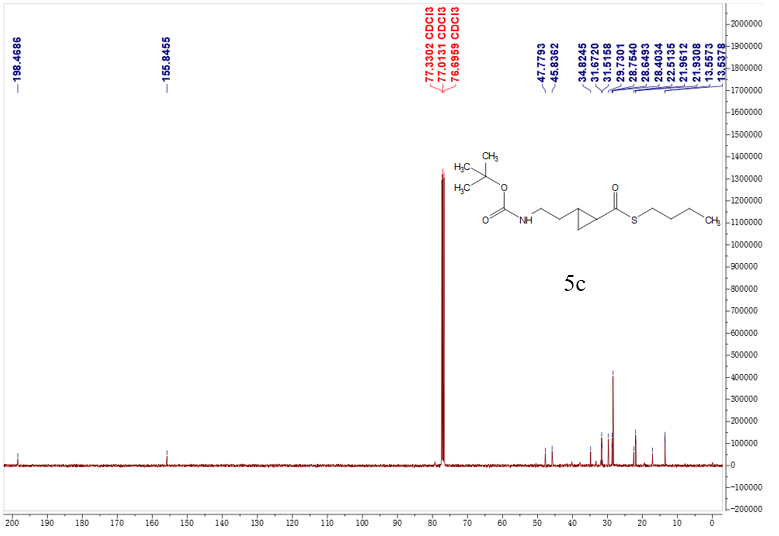


HRMS of **5c** (calcd. for C_21_H_29_NO_3_S [M+H] ^+^ 302.1785)


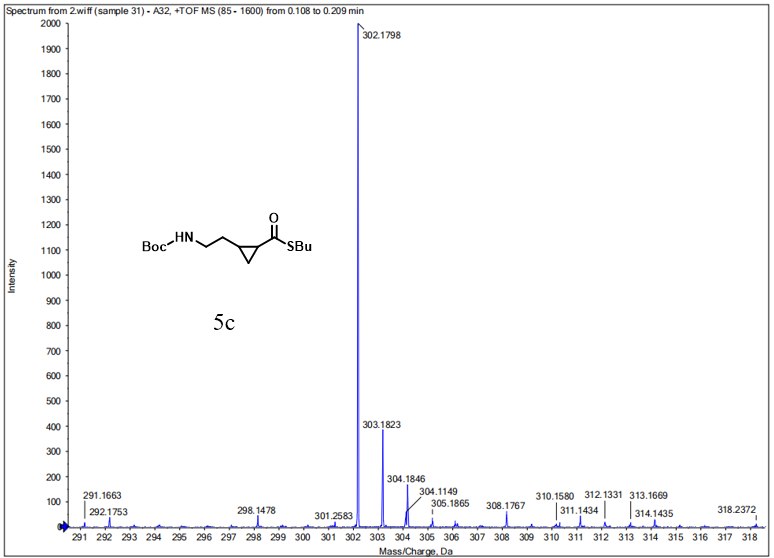


^1^H NMR (400 MHz, CDCl_3_) of **5d**


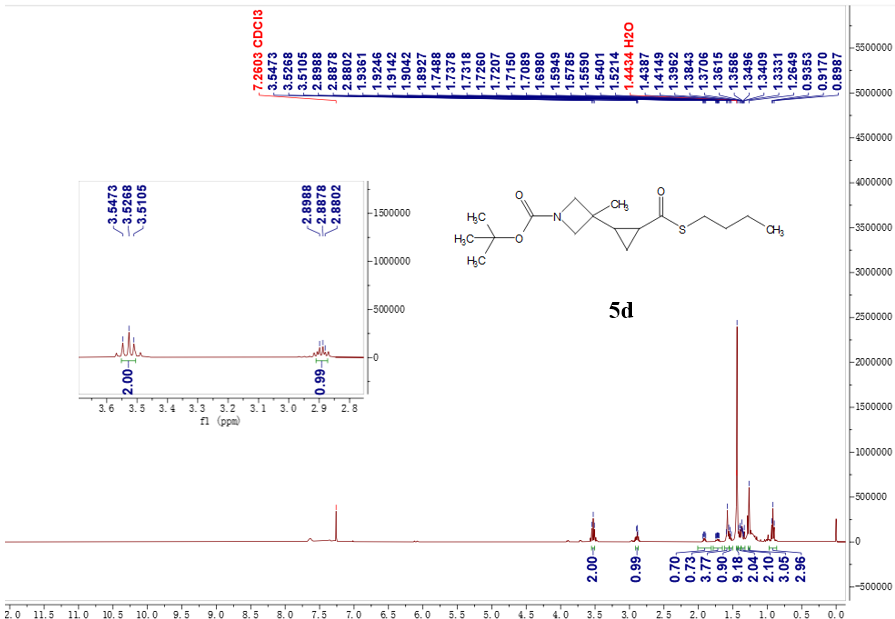


^13^C NMR (101 MHz, CDCl_3_) of **5d**


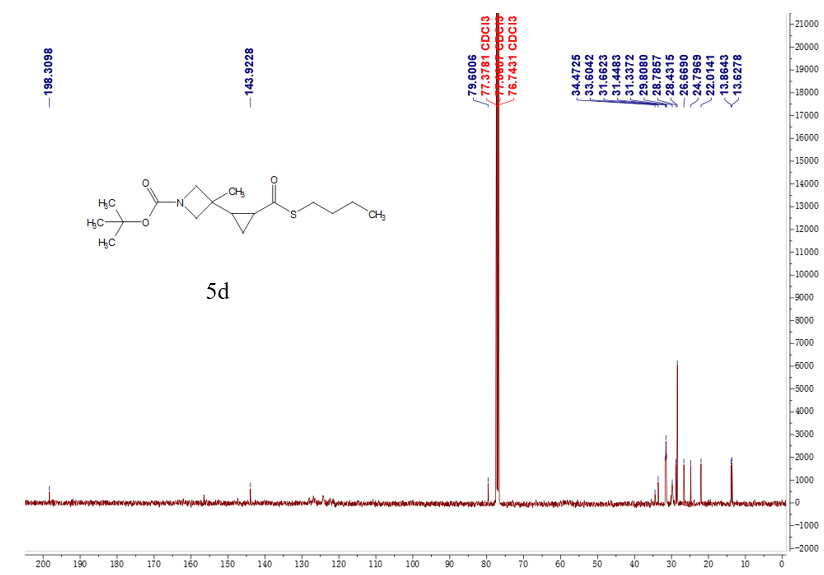


HRMS of **5d** (calcd. for C_21_H_29_NO_3_S [M+H] ^+^ 328.1470)


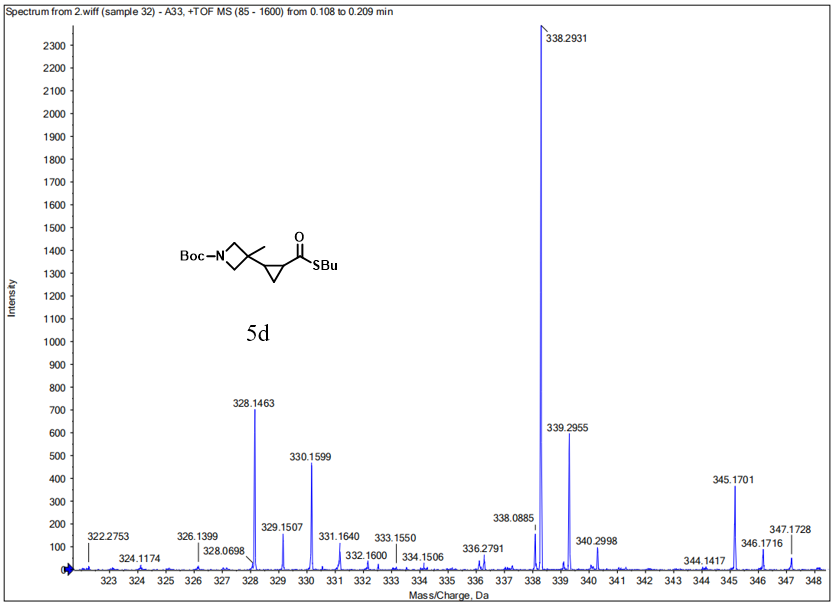


^1^H NMR (400 MHz, CDCl_3_) of **6a**


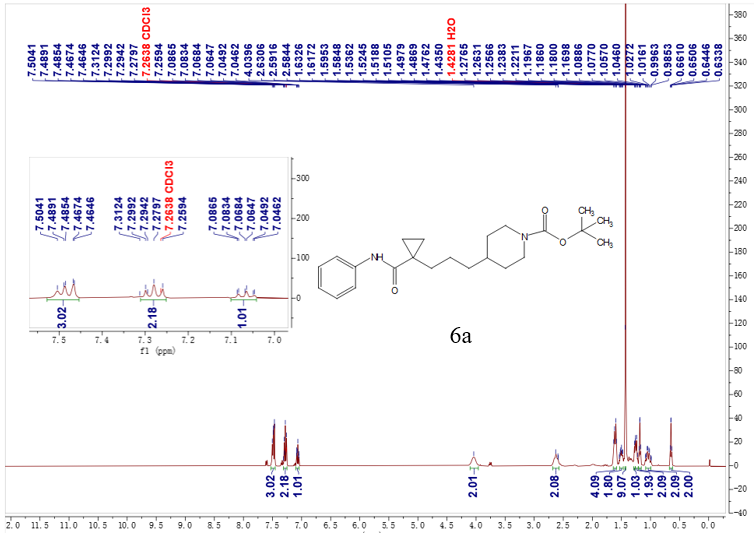


^13^C NMR (101 MHz, CDCl_3_) of **6a**


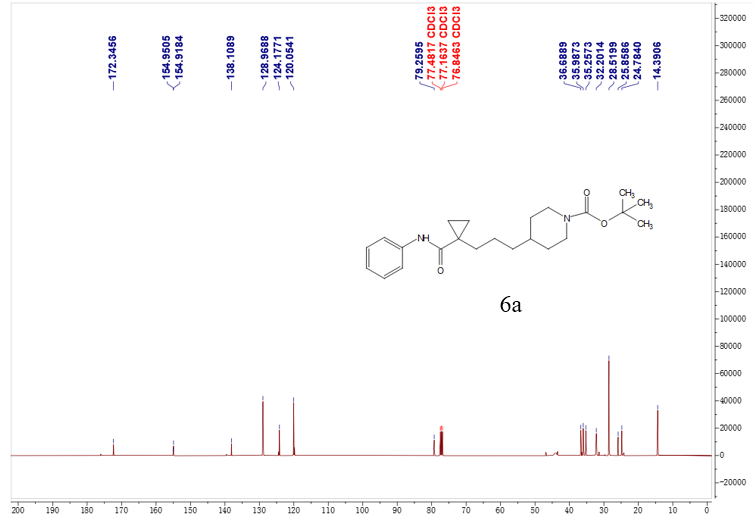


HRMS of **6a** (calcd for C_23_H_34_N_2_O_3_ [M+H] ^+^ 387.2642)


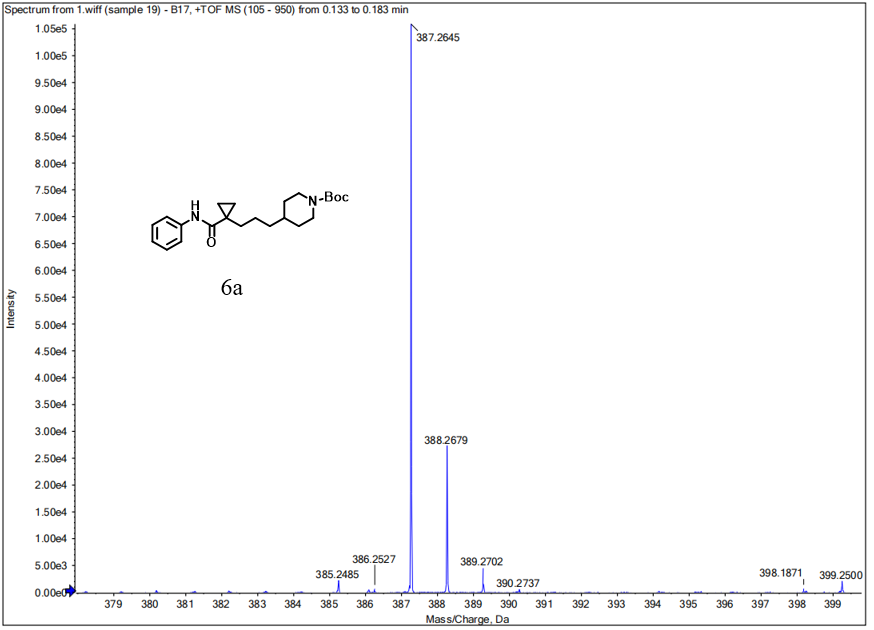


^1^H NMR (400 MHz, CDCl_3_) of **6b**


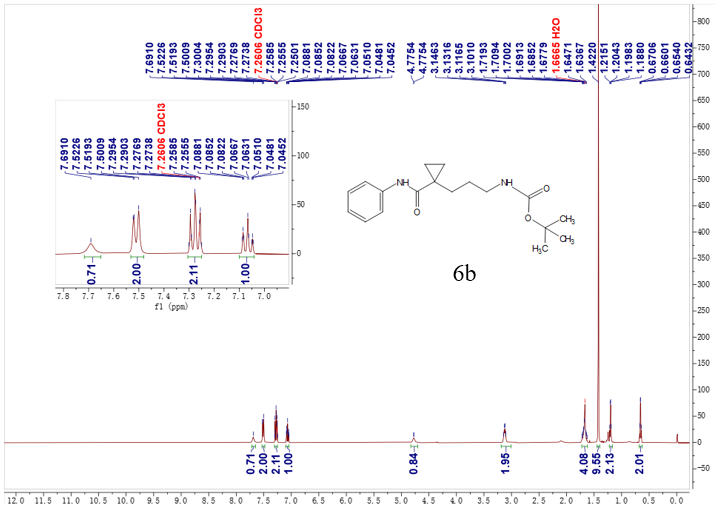


^13^C NMR (101 MHz, CDCl_3_) of **6b**


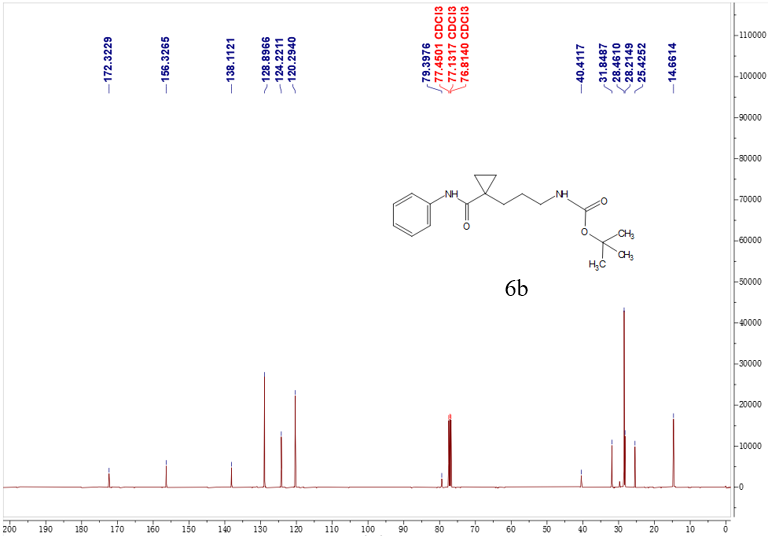


HRMS of **6b** (calcd for C_18_H_26_N_2_O_3_ [M+H] ^+^ 319.2016)


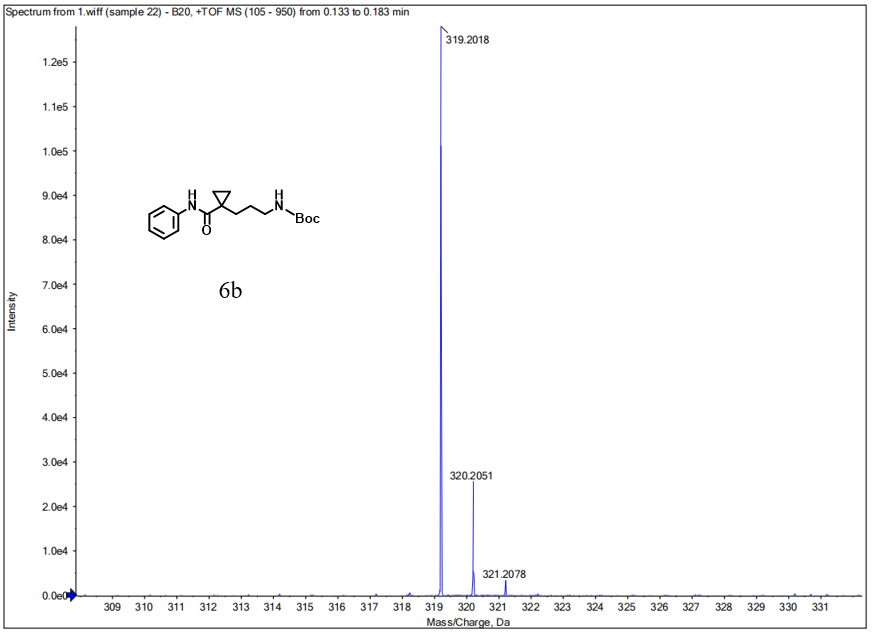


^1^H NMR (400 MHz, CDCl_3_) of **6c**


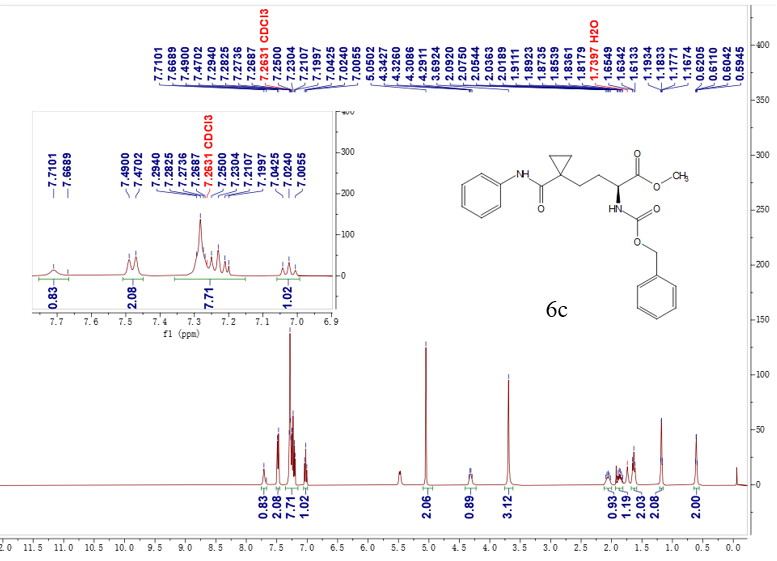


^13^C NMR (101 MHz, CDCl_3_) of **6c**


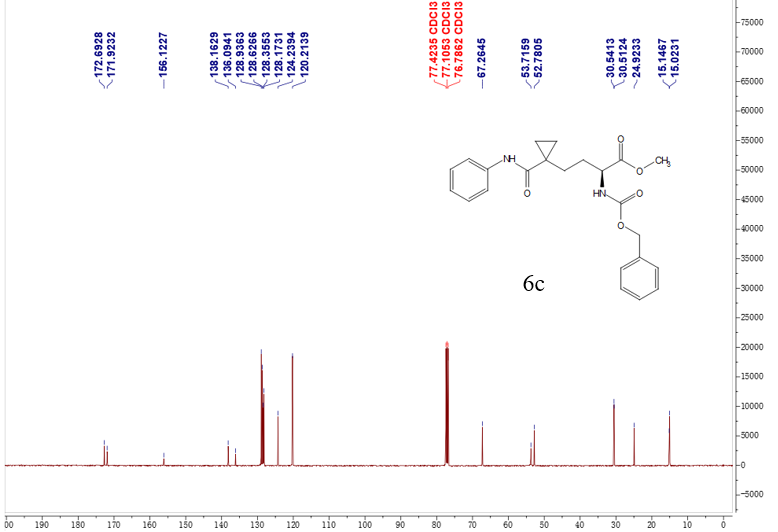


HRMS of 6c (calcd for C_23_H_26_N_2_O_5_ [M+H] ^+^ 411.1915)


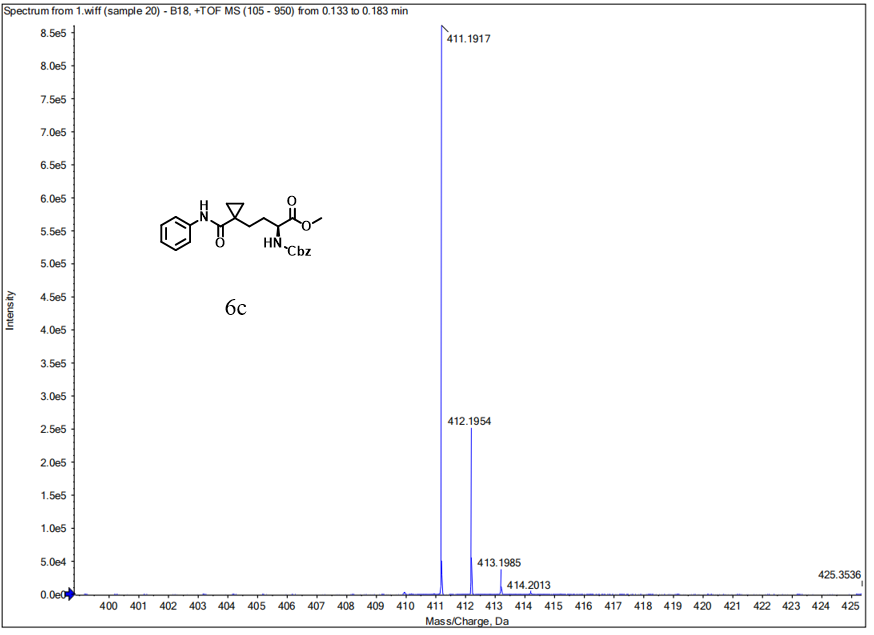


^1^H NMR (400 MHz, CDCl_3_) of **6d**


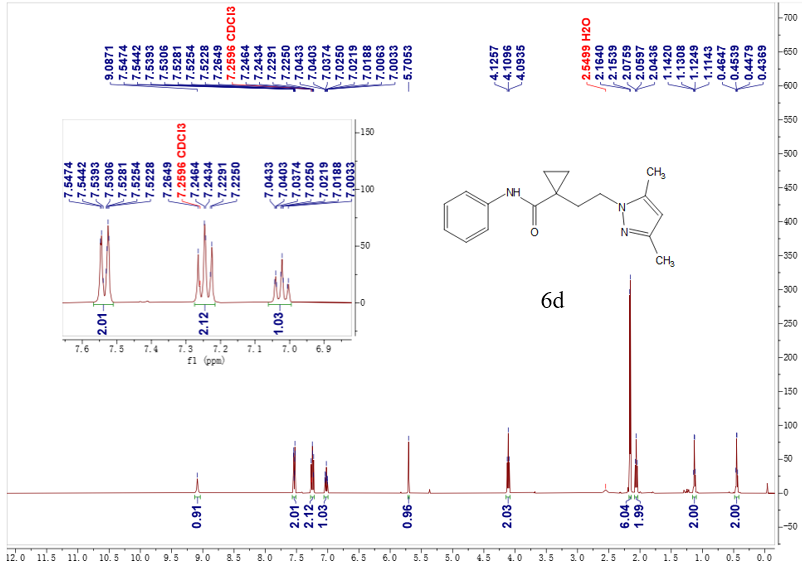


^13^C NMR (101 MHz, CDCl_3_) of **6d**


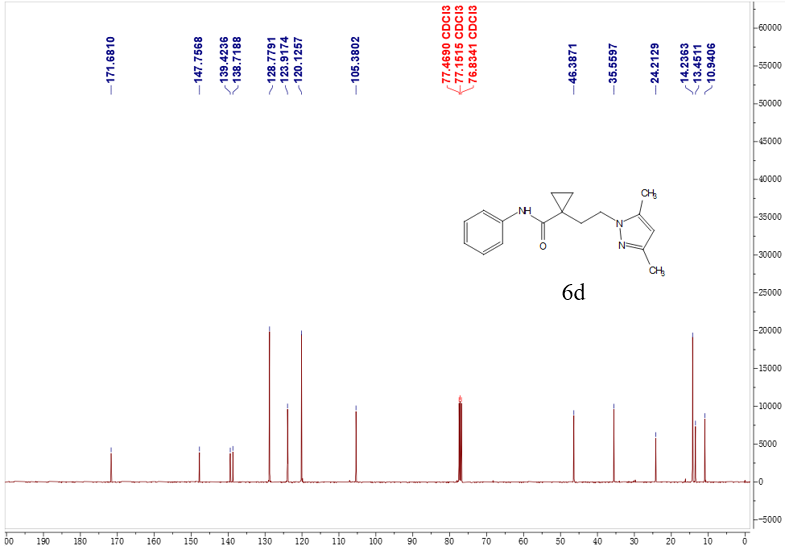


HRMS of **6d** (calcd for C_17_H_21_N_3_O [M+H] ^+^ 284.1757)


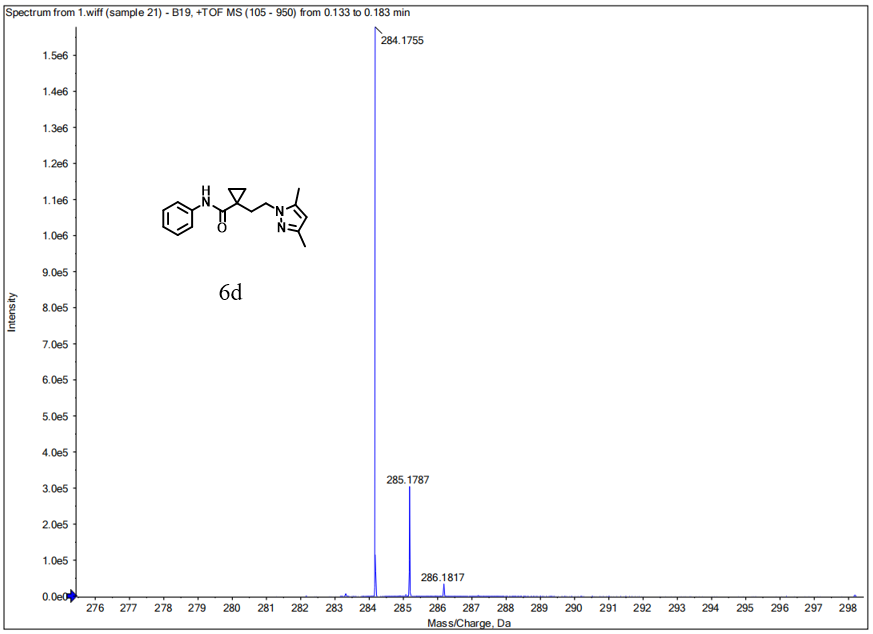


^1^H NMR (400 MHz, CDCl_3_) of **6e**


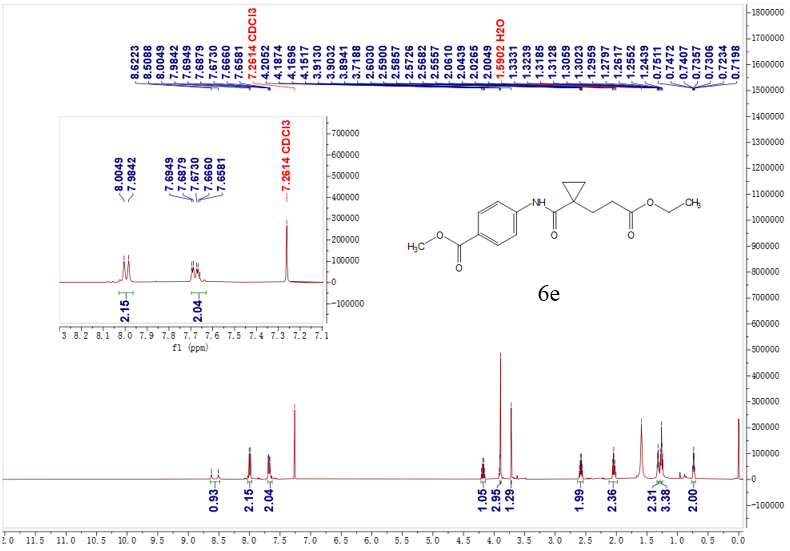


^13^C NMR (101 MHz, CDCl_3_) of **6e**


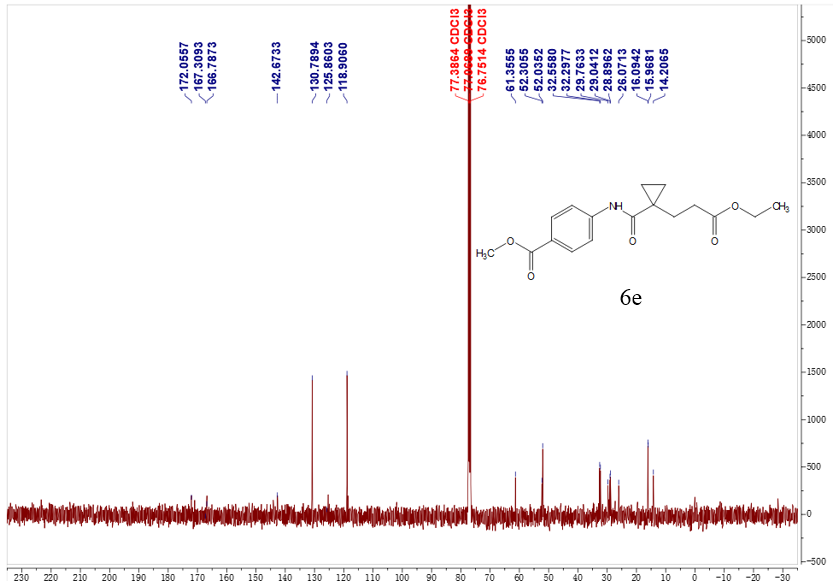


HRMS of **6e** (calcd for C_17_H_21_NO_5_ [M+H] ^+^ 320.1492)


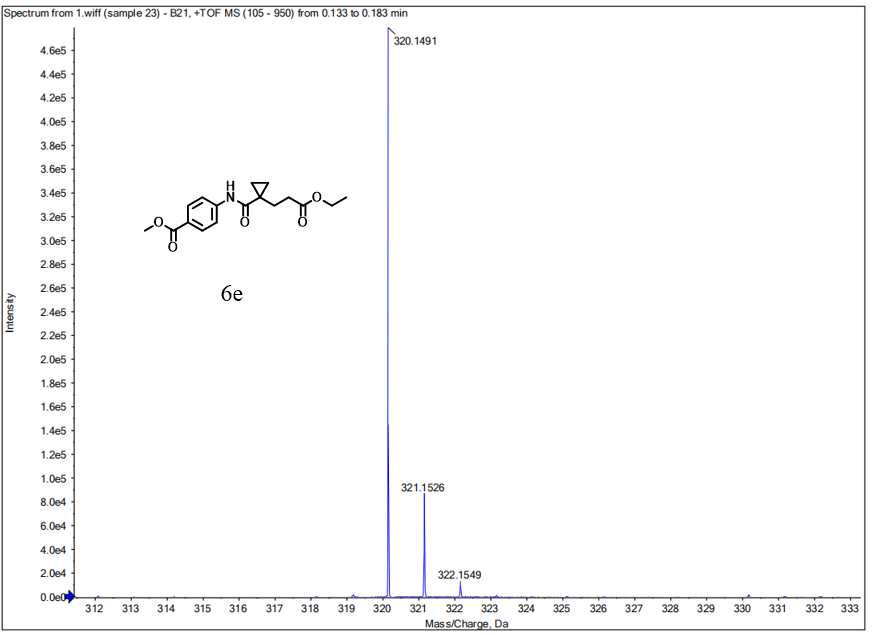


^1^H NMR (400 MHz, CDCl_3_) of **6f**


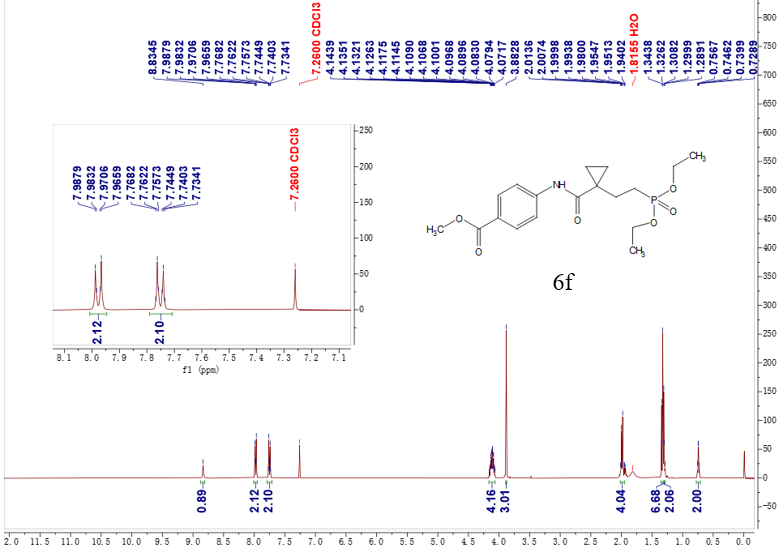


^13^C NMR (101 MHz, CDCl_3_) of **6f**


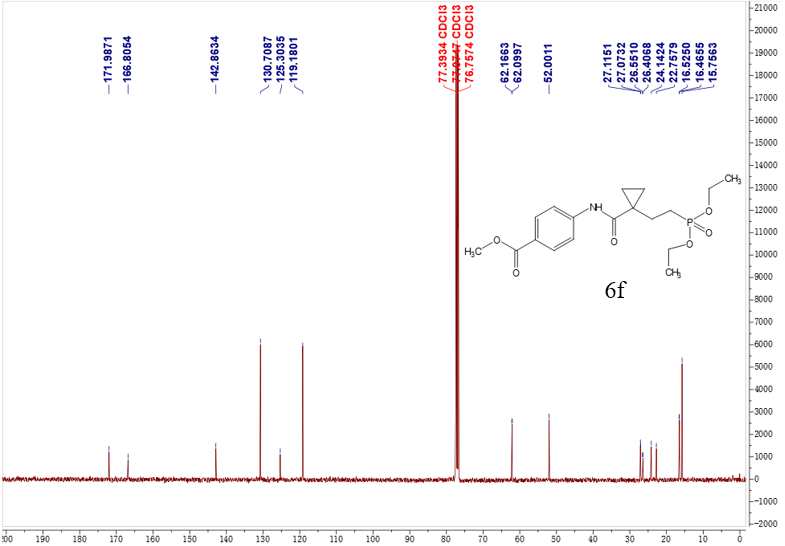


HRMS of **6f** (calcd for C_18_H_26_NO_6_P [M+H] ^+^ 384.1570)


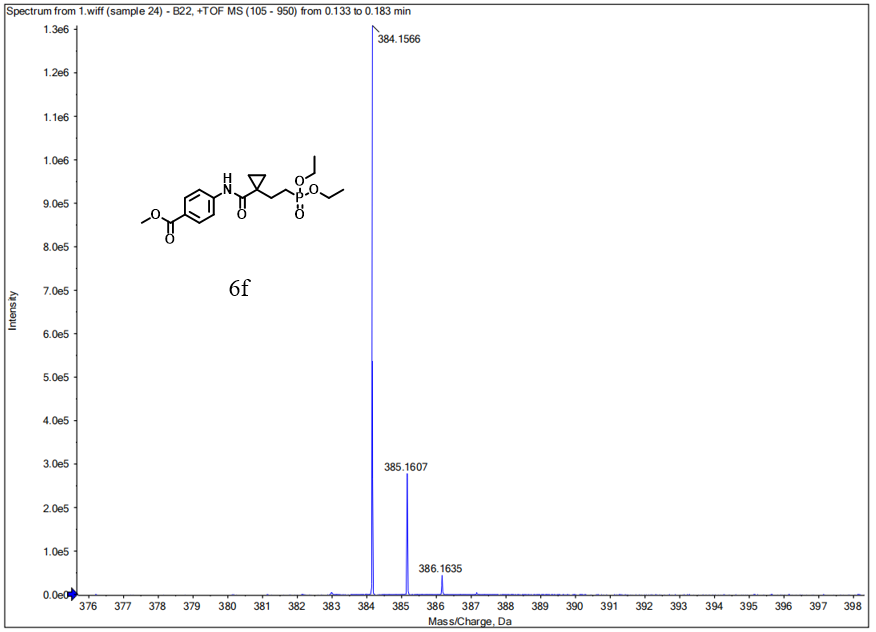


^1^H NMR (400 MHz, CDCl_3_) of **6g**


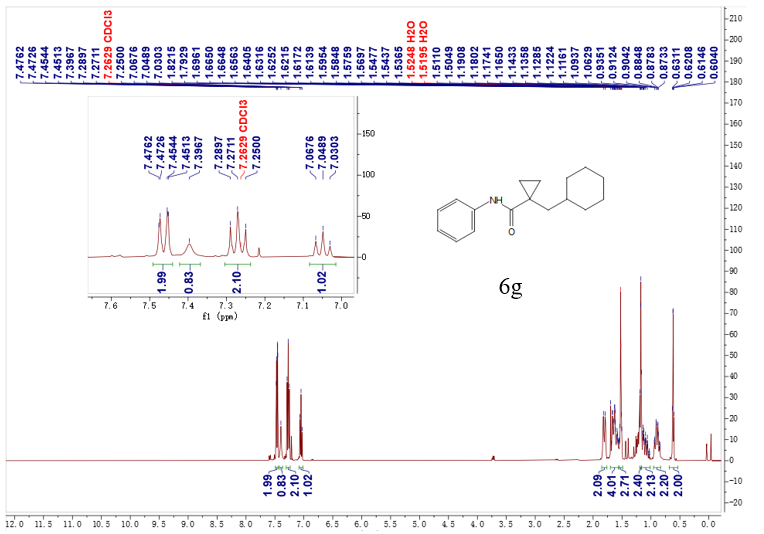


^13^C NMR (101 MHz, CDCl_3_) of **6g**


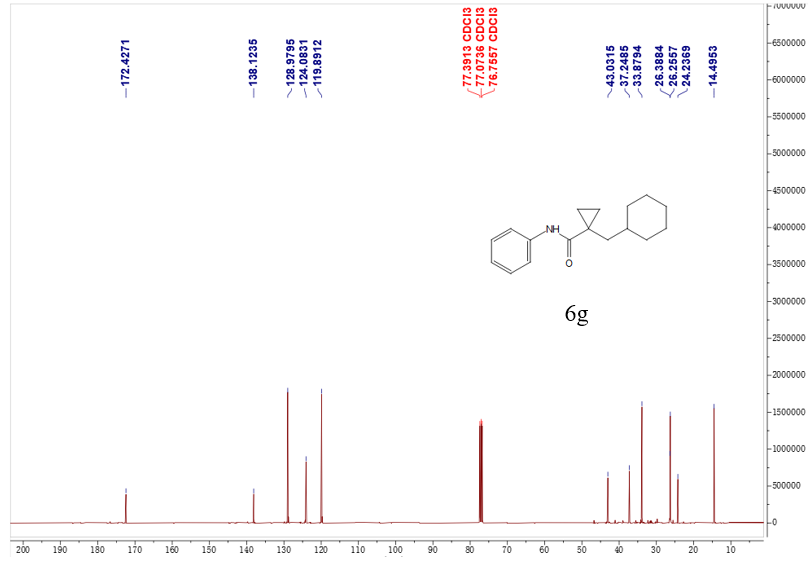


HRMS of **6g** (calcd for C_17_H_23_NO [M+H] ^+^ 258.1852)

^1^H NMR (400 MHz, CDCl_3_) of **6h**

^13^C NMR (101 MHz, CDCl_3_) of **6h**

HRMS of **6h** (calcd for C_23_H_32_N_2_O_5_ [M+H] ^+^ 417.2384)

^1^H NMR (500 MHz, CDCl_3_) of **6i**

^13^C NMR (126 MHz, CDCl_3_) of **6i**

HRMS of **6i** (calcd for C_17_H_21_NO_2_ [M+H] ^+^ 272.1645)

^1^H NMR (400 MHz, CDCl_3_) of **6j**

^13^C NMR (101 MHz, CDCl_3_) of **6j**

HRMS of **6j** (calcd for C_18_H_23_NO_3_ [M+H] ^+^ 302.1751)

^1^H NMR (400 MHz, CDCl_3_) of **6k**

^13^C NMR (126 MHz, CDCl_3_) of **6k**

HRMS of **6k** (calcd for C_16_H_21_NO_2_ [M+H] ^+^ 260.1645)

^1^H NMR (400 MHz, CDCl_3_) of **6l**

^13^C NMR (126 MHz, CDCl_3_) of **6l**

HRMS of **6l** (calcd for C_21_H_23_NO_3_ [M+H] ^+^ 338.1751)

^1^H NMR (400 MHz, CDCl_3_) of **6m**

^13^C NMR (126 MHz, CDCl_3_) of **6m**

HRMS of **6m** (calcd for C_22_H_24_N_2_O_3_ [M+H] ^+^ 365.1860)

^1^H NMR (400 MHz, CDCl_3_) of **6n**

^13^C NMR (126 MHz, CDCl_3_) of **6n**

HRMS of **6n** (calcd for C_23_H_26_N_2_O_3_ [M+H] ^+^ 379.2016)

^1^H NMR (400 MHz, CDCl_3_) of **6o**

^13^C NMR (101 MHz, CDCl_3_) of **6o**

HRMS of **6o** (calcd for C_24_H_34_N_2_O_4_ [M+H] ^+^ 415.2591)

^1^H NMR (400 MHz, CDCl_3_) of **6p**

^13^C NMR (101 MHz, CDCl_3_) of **6p**

HRMS of **6p** (calcd for C_23_H_32_N_2_O_3_ [M+H] ^+^ 385.2485)

^1^H NMR (400 MHz, CDCl_3_) of **6q**

^13^C NMR (126 MHz, CDCl_3_) of **6q**

HRMS of **6q** (calcd for C_14_H_19_NO [M+H] ^+^ 218.1540)

^1^H NMR (400 MHz, CDCl_3_) of **6r**

^13^C NMR (126 MHz, CDCl_3_) of **6r**

HRMS of **6r** (calcd. for C_17_H_25_NO_2_ [M+H] ^+^276.1958)

^1^H NMR (400 MHz, CDCl_3_) of **6s**

^13^C NMR (101 MHz, CDCl_3_) of **6s**

HRMS of **6s** (calcd for C_23_H_27_NO_3_ [M+H] ^+^ 366.2063)

^1^H NMR (400 MHz, CDCl_3_) of **6t**

^13^C NMR (101 MHz, CDCl_3_) of **6t**

HRMS of **6t** (calcd for C_20_H_25_NO_3_ [M+H] ^+^ 328.1907)

^1^H NMR (400 MHz, CDCl_3_) of **6u**

^13^C NMR (101 MHz, CDCl_3_) of **6u**

HRMS of **6u** (calcd for C_23_H_29_NO_3_ [M+H] ^+^ 368.2220)

^1^H NMR (400 MHz, CDCl_3_) of **6v**

^13^C NMR (101 MHz, CDCl_3_) of **6v**

HRMS of **6v** (calcd for C_20_H_28_N_2_O_3_ [M+H] ^+^ 345.2172)

^1^H NMR (400 MHz, CDCl_3_) of **6w**

^13^C NMR (101 MHz, CDCl_3_) of **6w**

HRMS of **6w** (calcd for C_22_H_32_N_2_O_3_ [M+H] ^+^ 373.2485)

^1^H NMR (400 MHz, CDCl_3_) of **6x**

^13^C NMR (101 MHz, CDCl_3_) of **6x**

HRMS of **6x** (calcd for C_20_H_30_N_2_O_3_ [M+H] ^+^ 347.2329)

^1^H NMR (400 MHz, CDCl_3_) of **6y**

^13^C NMR (101 MHz, CDCl_3_) of **6y**

HRMS of **6y** (calcd for C_17_H_23_NO_3_ [M+H] ^+^ 290.1750)

^1^H NMR (400 MHz, CDCl_3_) of **6z**

^13^C NMR (101 MHz, CDCl_3_) of **6z**

HRMS of **6z** (calcd for C_24_H_29_NO_3_ [M+H] ^+^ 380.2215)

^1^H NMR (400 MHz, CDCl_3_) of **6aa**

^13^C NMR (101 MHz, CDCl_3_) of **6aa**

HRMS of **6aa** (calcd for C_18_H_25_NO_4_ [M+H] ^+^ 320.1856)

^1^H NMR (400 MHz, CDCl_3_) of **6ab**

^13^C NMR (101 MHz, CDCl_3_) of **6ab**

HRMS of **6ab** (calcd for C_17_H_21_NO_4_ [M+H] ^+^ 304.1543)

^1^H NMR (400 MHz, CDCl_3_) of **6ac**

^13^C NMR (101 MHz, CDCl_3_) of **6ac**

HRMS of **6ac** (calcd for C_19_H_25_NO_3_ [M+H] ^+^ 316.1907)

^1^H NMR (400 MHz, CDCl_3_) of **7**

^13^C NMR (101 MHz, CDCl_3_) of **7**

HRMS of **7** (calcd for C_36_H_49_NO_5_ [M+H] ^+^ 576.3683)

^1^H NMR (400 MHz, CDCl_3_) of **8**

^13^C NMR (101 MHz, CDCl_3_) of **8**

HRMS of **8** (calcd for C_45_H_47_NO_6_ [M+H] ^+^ 698.3476)

^1^H NMR (400 MHz, CDCl_3_) of **9**

^13^C NMR (101 MHz, CDCl_3_) of **9**

HRMS of **9** (calcd for C_22_H_29_NO_6_ [M+H] ^+^ 404.2067)

^1^H NMR (400 MHz, CDCl_3_) of **10**

^13^C NMR (101 MHz, CDCl_3_) of **10**

HRMS of **10** (calcd. for C_34_H_37_ClN_8_O_3_ [M+H] ^+^ 641.2750)

^1^H NMR (400 MHz, CDCl_3_) of **11**

^13^C NMR (101 MHz, CDCl_3_) of **11**

HRMS of **11** (calcd. for C_42_H_43_N_3_O_7_ [M+ Na] ^+^ 724.2999)

^1^H NMR (400 MHz, CDCl_3_) of **12**

^13^C NMR (101 MHz, CDCl_3_) of **12**

HRMS of **12** (calcd for C_49_H_46_N_6_O_6_ [M+H] ^+^ 815.3551)

^1^H NMR (400 MHz, CDCl_3_) of **13**

^13^C NMR (101 MHz, CDCl_3_) of **13**

HRMS of **13** (calcd for C_38_H_57_NO [M+H] ^+^ 544.4512)

^1^H NMR (400 MHz, CDCl_3_) of **14**

^13^C NMR (101 MHz, CDCl_3_) of **14**

HRMS of **14** (calcd for C_32_H_41_NO_3_ [M+H] ^+^ 488.3159)

^1^H NMR (400 MHz, CDCl_3_) of **15**

^13^C NMR (101 MHz, CDCl_3_) of **15**

HRMS of **15** (calcd for C_21_H_25_N_3_O_4_ [M+H] ^+^ 384.1917)

^1^H NMR (400 MHz, CDCl_3_) of **16**

^13^C NMR (101 MHz, CDCl_3_) of **16**

HRMS of 16 (calcd for C_29_H_38_ClN_3_O_2_S [M+H] ^+^ 528.2442)

^1^H NMR (400 MHz, CDCl_3_) of **24**

^1^H NMR (400 MHz, CDCl_3_) of ***E*-25**

^13^C NMR (101 MHz, CDCl_3_) of ***E*-25**

HRMS of ***E*-25** (calcd for C_24_H_27_NO_4_ [M+H] ^+^ 394.2013)

^1^H NMR (400 MHz, CDCl_3_) of ***Z*-25**

^13^C NMR (101 MHz, CDCl_3_) of ***Z*-25**

HRMS of ***Z*-25** (calcd for C_24_H_27_NO_4_ [M+H] ^+^ 394.2013)
